# 1′-Acetoxychavicol Acetate Selectively Downregulates Tumor Necrosis Factor Receptor-Associated Factor 2 (TRAF2) Expression

**DOI:** 10.3390/molecules30061243

**Published:** 2025-03-10

**Authors:** Chihiro Moriwaki, Shingo Takahashi, Nhat Thi Vu, Yasunobu Miyake, Takao Kataoka

**Affiliations:** 1 Department of Applied Biology, Kyoto Institute of Technology, Matsugasaki, Sakyo-ku, Kyoto 606-8585, Japan; 2 Division of Molecular and Cellular Immunoscience, Department of Biomolecular Sciences, Faculty of Medicine, Saga University, Nabeshima, Saga 849-8501, Japan; 3 Biomedical Research Center, Kyoto Institute of Technology, Matsugasaki, Sakyo-ku, Kyoto 606-8585, Japan

**Keywords:** 1′-acetoxychavicol acetate, TRAF2, TNF-α, NF-κB, ICAM-1, proteasome

## Abstract

1′-Acetoxychavicol acetate (ACA) is a natural compound derived from rhizomes of the Zingiberaceae family that suppresses the nuclear factor κB (NF-κB) signaling pathway; however, the underlying mechanisms remain unclear. Therefore, the present study investigated the molecular mechanisms by which ACA inhibits the NF-κB signaling pathway in human lung adenocarcinoma A549 cells. The results obtained showed ACA decreased tumor necrosis factor (TNF)-α-induced intercellular adhesion molecule-1 (ICAM-1) expression in A549 cells. It also inhibited TNF-α-induced ICAM-1 mRNA expression and ICAM-1 promoter-driven and NF-κB-responsive luciferase reporter activities. Furthermore, the TNF-α-induced degradation of the inhibitor of NF-κB α protein in the NF-κB signaling pathway was suppressed by ACA. Although ACA did not affect TNF receptor 1, TNF receptor-associated death domain, or receptor-interacting protein kinase 1 protein expression, it selectively downregulated TNF receptor-associated factor 2 (TRAF2) protein expression. The proteasome inhibitor MG-132, but not inhibitors of caspases or lysosomal degradation, attenuated ACA-induced reductions in TRAF2 expression. ACA also downregulated TRAF2 protein expression in human fibrosarcoma HT-1080 cells. This is the first study to demonstrate that ACA selectively downregulates TRAF2 protein expression.

## 1. Introduction

Pro-inflammatory cytokines are secreted predominantly by macrophages at sites of inflammation [1,2]. Pro-inflammatory cytokines up-regulate the expression of adhesion molecules, such as intercellular adhesion molecule-1 (ICAM-1) in various cell types, including endothelial, epithelial, immune, and tumor cells [3]. In vascular endothelial cells, ICAM-1 plays an essential role in the recruitment of circulating leukocytes and their transmigration from the interior of blood vessels to extravascular sites of inflammation [4,5]. ICAM-1 expression is also upregulated in several cancers, such as lung and colorectal cancers, and is associated with their malignancy, metastasis, and poor prognosis [6,7]. ICAM-1 expression is mainly regulated by the transcription nuclear factor κB (NF-κB) [8,9].

Tumor necrosis factor α (TNF-α) is one of the pro-inflammatory cytokines, and interacts with two cell-surface receptors: TNF receptors 1 and 2 [10]. TNF-α has been implicated in the pathogenesis of inflammatory diseases and in the promotion and progression of cancer [11,12]. TNF receptor 1 is ubiquitously expressed, whereas TNF receptor 2 is more restrictedly expressed [13,14]. TNF receptor 1 contains a death domain (DD), and interacts with multiple adaptor proteins, including TNF receptor-associated death domain (TRADD), receptor-interacting protein kinase 1 (RIPK1), and TNF receptor-associated factor (TRAF2), leading to the formation of the TNF receptor 1 signaling complex [12,13,14]. The TNF receptor 1 signaling complex is required for the activation of the inhibitor of NF-κB (IκB) kinase complex, which converts IκBα to its phosphorylated form [15,16]. Phosphorylated IκBα is immediately ubiquitinated and degraded by the proteasome, allowing for the release of sequestered NF-κB heterodimers into the cytoplasm and their nuclear translocation [17,18]. The NF-κB heterodimers induce the transcription of many genes, such as those regulating inflammation, tumor promotion and metastasis [19,20].

**Figure 1:**
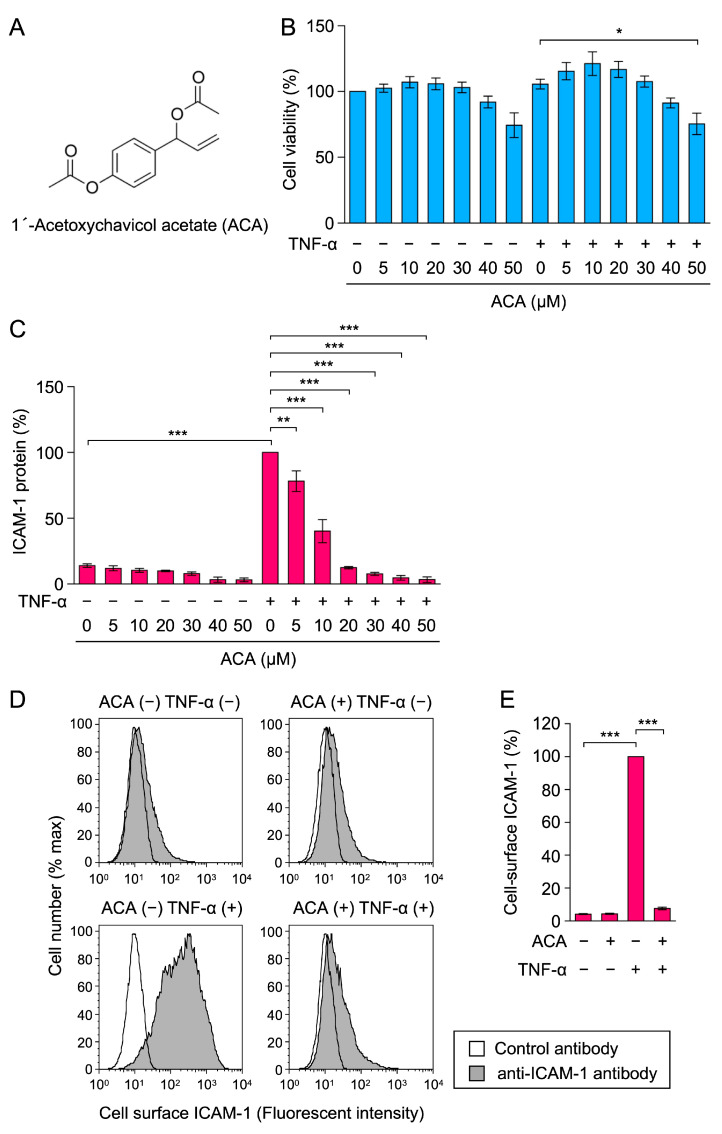
ACA inhibited TNF-α-induced ICAM-1 expression in A549 cells. (**A**) Structure of 1′-acetoxychavicol acetate (ACA). (**B**,**C**) A549 cells were preincubated with ACA for 1 h and then incubated with (+) or without (−) TNF-α (2.5 ng/mL) for 6 h in the presence or absence of ACA. Cell viability (%) is shown as the mean ± standard error of three independent experiments (**B**). Cell-ELISA was used to measure the amount of the ICAM-1 protein. ICAM-1 protein (%) is shown as the mean ± standard error of three independent experiments (**C**). (**D**,**E**) A549 cells were preincubated with (+) or without (−) ACA for 1 h, and were then incubated with (+) or without (−) TNF-α (2.5 ng/mL) in the presence (+) or absence (−) of ACA (30 µM). Cells were stained with a control antibody (white area) and an anti-ICAM-1 antibody (gray area) followed by a fluorescent secondary antibody, and were then analyzed by flow cytometry. Representative histograms are shown (**D**). Cell-surface ICAM-1 (%) is shown as the mean ± standard error of three independent experiments (**E**). * *p* < 0.05, ** *p* < 0.01, and *** *p* < 0.001.

1′-Acetoxychavicol acetate (ACA) (Figure 1A) is a natural compound derived from plants of the Zingiberaceae family (e.g., *Alpinia galanga*), which are used as a spice and in folk medicine [21,22]. ACA exerts various biological effects, including anticancer and anti-inflammatory activities [21,22]. Related to anti-inflammatory activity, ACA has been reported to inhibit the lipopolysaccharide (LPS)-induced expression of inducible nitric oxide synthase and cyclooxygenase 2 in murine macrophage cell line RAW264 cells and mouse peritoneal macrophages [23,24,25,26]. Previous studies also reported that ACA inhibited IκBα degradation and the subsequent NF-κB signaling pathway in macrophages [23,24,26,27,28]. In several human cancer cell lines, ACA prevented the activation of NF-κB, which was activated constitutively or by the addition of inflammatory and carcinogenic agents [29,30,31] and in vivo tumor models [32,33]. However, the mechanisms of action of ACA on the NF-κB signaling pathway remain unclear.

To search anti-inflammatory and anticancer agents, we established a screening system to monitor the NF-κB-dependent expression of cell-surface ICAM-1 upon stimulation with pro-inflammatory cytokines in human lung adenocarcinoma A549 cells and human vascular endothelial cells [34]. During our screening, ACA was identified as a natural compound that inhibits TNF-α-induced ICAM-1 expression in A549 cells. Therefore, further investigations were conducted to elucidate the mechanisms by which ACA inhibits the NF-κB signaling pathway. In the present study, ACA was found to selectively downregulate the expression of TRAF2 among the components of the TNF receptor 1 signaling complex.

## 2. Results

### 2.1. ACA Inhibited TNF-α-Induced ICAM-1 Protein Expression in A549 Cells

Human lung adenocarcinoma A549 cells were preincubated with ACA for 1 h, and were then incubated with or without TNF-α for 6 h. Cell viability was assessed by 3-(4,5-dimetylthiazol-2-yl)-2,5-diphenyltetrazolium bromide (MTT)-reducing activity. ACA did not markedly affect the viability of A549 cells at concentrations up to 40 µM (Figure 1B).

ICAM-1 protein expression was evaluated by a cell enzyme-linked immunosorbent assay (cell-ELISA). ACA was found to inhibit TNF-α-induced ICAM-1 protein expression in a dose-dependent manner and basal level at concentrations higher than 20 µM (Figure 1C). The results of the flow cytometry analysis confirmed that ACA at 30 µM inhibited TNF-α-induced cell-surface ICAM-1 expression to the unstimulated level (Figure 1D,E). These results show that ACA inhibited TNF-α-induced ICAM-1 expression at concentrations that did not affect cell viability.

**Figure 2:**
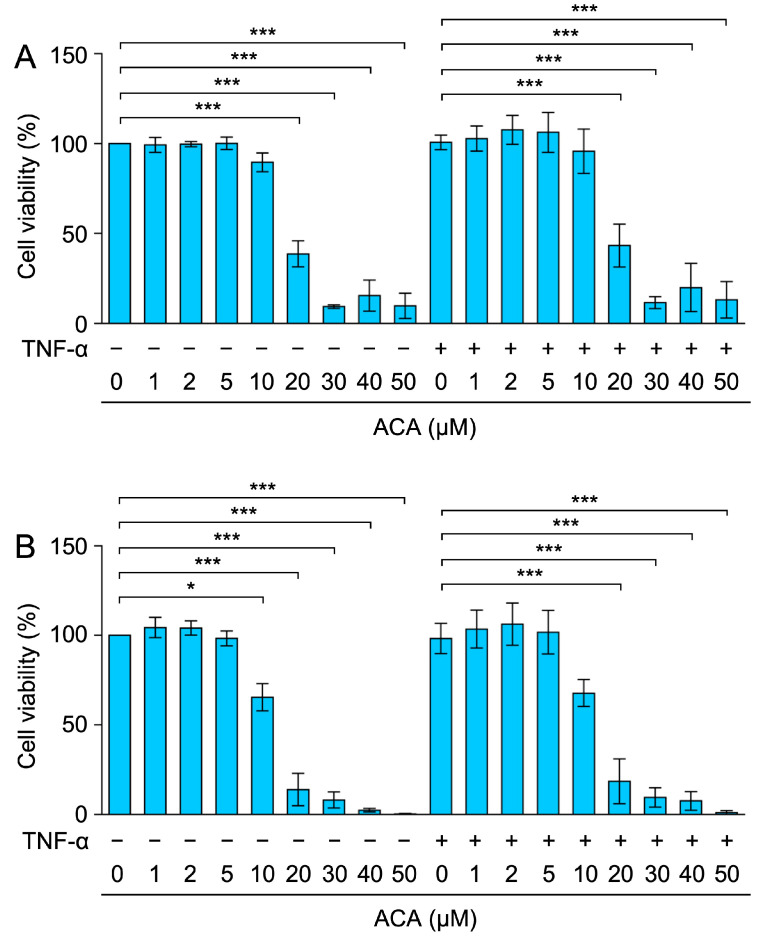
Effect of ACA on the viability of A549 cells for longer periods of time. (**A**,**B**) A549 cells were preincubated with ACA for 1 h and then incubated with (+) or without (−) TNF-α (2.5 ng/mL) for 24 h (**A**) and 48 h (**B**) in the presence or absence of ACA. Cell viability (%) is shown as the mean ± standard error of three independent experiments. * *p* < 0.05 and *** *p* < 0.001.

Following preincubation with ACA for 1 h, A549 cells were incubated with or without TNF-α for 24 and 48 h and then subjected to the MTT assay to assess cell viability. The results obtained showed that cell viability was reduced by ACA at concentrations higher than 20 µM for 24 h or 48 h (Figure 2A,B). Therefore, ACA reduced the viability of A549 cells more effectively when they were incubated for longer periods of time, which may be related to its anticancer activity.

### 2.2. Glutathione and N-Acetyl-L-Cysteine (NAC) Did Not Reverse the Inhibitory Activity of ACA on TNF-α-Induced ICAM-1 Expression

**Figure 3:**
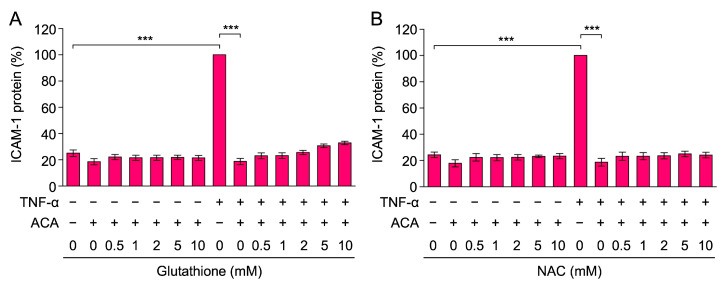
Glutathione and NAC did not reverse the inhibitory effect of ACA on TNF-α-induced ICAM-1 expression. (**A**,**B**) A549 cells were preincubated with glutathione (**A**) and NAC (**B**) for 1 h, and were then incubated with (+) or without (−) ACA (40 µM) for 1 h, followed by an incubation with (+) or without (−) TNF-α (2.5 ng/mL) for 6 h in the presence of compounds. The amount of the ICAM-1 protein was measured by cell-ELISA. ICAM-1 protein (%) is shown as the mean ± standard error of three independent experiments. *** *p* < 0.001.

Glutathione and NAC are often used as antioxidants that suppress reactive oxygen species (ROS). ACA has been shown to increase ROS generation in cancer cells, and glutathione and NAC suppressed the reduction in cell viability caused by ACA [35,36,37,38,39]. Therefore, we examined the abilities of glutathione and NAC to reverse the inhibitory effects of ACA on TNF-α-induced ICAM-1 expression. A549 cells were preincubated with glutathione and NAC for 1 h, followed by an incubation with ACA for 1 h, and were then stimulated with TNF-α for 6 h. Neither glutathione nor NAC at concentrations up to 10 mM affected the inhibitory activity of ACA on TNF-α-induced ICAM-1 expression (Figure 3A,B). Thus, these data suggested that the inhibitory activity of ACA on TNF-α-induced ICAM-1 expression was not due to ROS generation.

### 2.3. ACA Inhibited TNF-α-Induced ICAM-1 mRNA Expression in A549 Cells

**Figure 4:**
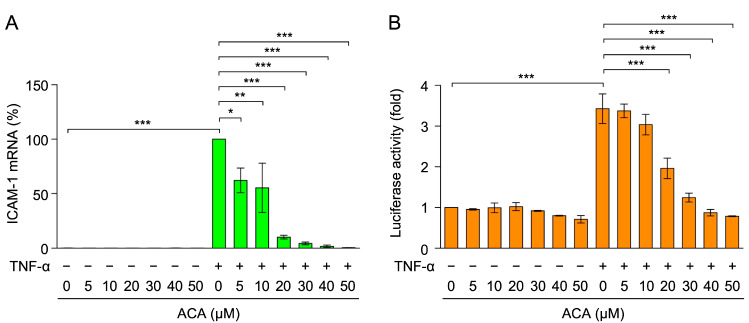
ACA inhibited TNF-α-induced ICAM-1 mRNA expression in A549 cells. (**A**) A549 cells were preincubated with ACA for 1 h, and were then incubated with (+) or without (−) TNF-α (2.5 ng/mL) for 2 h in the presence or absence of ACA. Real-time PCR was used to measure the amount of mRNA. ICAM-1 mRNA expression was normalized to β-actin mRNA expression. ICAM-1 mRNA (%) is shown as the mean ± standard error of four independent experiments. (**B**) A549 cells were transiently transfected with reporter plasmids encoding ICAM-1 promoter-driven firefly luciferase, together with a reporter plasmid encoding CMV promoter-driven *Renilla* luciferase for 24 h. Transfected A549 cells were preincubated with ACA for 1 h, and were then incubated with (+) or without (−) TNF-α (2.5 ng/mL) for 2.5 h in the presence or absence of ACA. Firefly luciferase activity was normalized to *Renilla* luciferase activity. Luciferase activity (fold) is shown as the mean ± standard error of three independent experiments. * *p* < 0.05, ** *p* < 0.01, and *** *p* < 0.001.

To investigate the effects of ACA on TNF-α-induced ICAM-1 expression at the mRNA level, total RNA was extracted and analyzed by reverse-transcriptase real-time PCR. TNF-α up-regulated the expression of ICAM-1 mRNA within 2 h (Figure 4A). ACA inhibited the TNF-α-induced expression of ICAM-1 mRNA in a dose-dependent manner and to the basal level at concentrations higher than 20 µM (Figure 4A).

The luciferase reporter driven by the ICAM-1 promoter (−1604 to +40) was used to evaluate the effects of ACA on ICAM-1 transcription. A549 cells were transfected with an expression vector encoding the ICAM-1 promoter-driven firefly luciferase reporter. TNF-α up-regulated ICAM-1 promoter-driven luciferase activity within 2.5 h (Figure 4B). ACA decreased TNF-α-induced luciferase activity at concentrations higher than 20 µM (Figure 4B). These results indicate that ACA inhibited TNF-α-induced ICAM-1 expression at the transcriptional level.

### 2.4. ACA Inhibited the TNF-α-Induced NF-κB Signaling Pathway in A549 Cells

**Figure 5:**
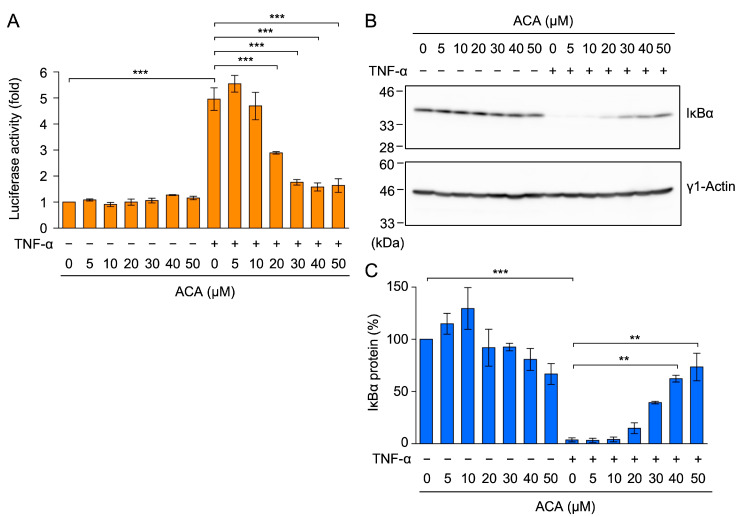
ACA inhibited the TNF-α-induced NF-κB signaling pathway in A549 cells. (**A**) A549 cells were transiently transfected with reporter plasmids encoding NF-κB-responsive firefly luciferase, together with a reporter plasmid encoding CMV promoter-driven *Renilla* luciferase for 24 h. Transfected A549 cells were preincubated with ACA for 1 h, and were then incubated with (+) or without (−) TNF-α (2.5 ng/mL) for 2.5 h in the presence or absence of ACA. Firefly luciferase activity was normalized to *Renilla* luciferase activity. Luciferase activity (fold) is shown as the mean ± standard error of three independent experiments. (**B**,**C**) A549 cells were preincubated with ACA for 1 h, and were then incubated with (+) or without (−) TNF-α (2.5 ng/mL) for 15 min in the presence or absence of ACA. Western blotting was performed to analyze cell lysates. Representative blots of IκBα and γ1-actin expression are shown (**B**). The amount of the γ1-actin protein was used to normalize the amount of the IκBα protein. IκBα protein (%) is shown as the mean ± standard error of three independent experiments (**C**). ** *p* < 0.01 and *** *p* < 0.001. Original blots are shown in Figures S1–S4.

TNF-α-induced ICAM-1 transcription is mainly up-regulated by NF-κB transcription factors [8,9]. When the NF-κB luciferase reporter containing two copies of κB sites was used, TNF-α increased NF-κB-responsive luciferase reporter activity to a level similar to that of the ICAM-1 promoter-driven luciferase reporter (Figure 5A). ACA reduced TNF-α-induced NF-κB-responsive luciferase activity at concentrations higher than 20 µM (Figure 5A). These results demonstrate that ACA inhibited TNF-α-induced NF-κB-dependent transcription.

NF-κB transcription factors are associated with the IκBα protein in the cytosol, and a TNF-α stimulation has been shown to induce the proteasome-dependent degradation of the IκBα protein [17,18]. In A549 cells, TNF-α induced the degradation of the IκBα protein within 15 min (Figure 5B,C). ACA inhibited the TNF-α-induced degradation of the IκBα protein in a dose-dependent manner (Figure 5B,C). These results show that ACA inhibited the TNF-α-induced NF-κB signaling pathway.

### 2.5. ACA Selectively Downregulated the Expression of TRAF2 in A549 Cells

**Figure 6:**
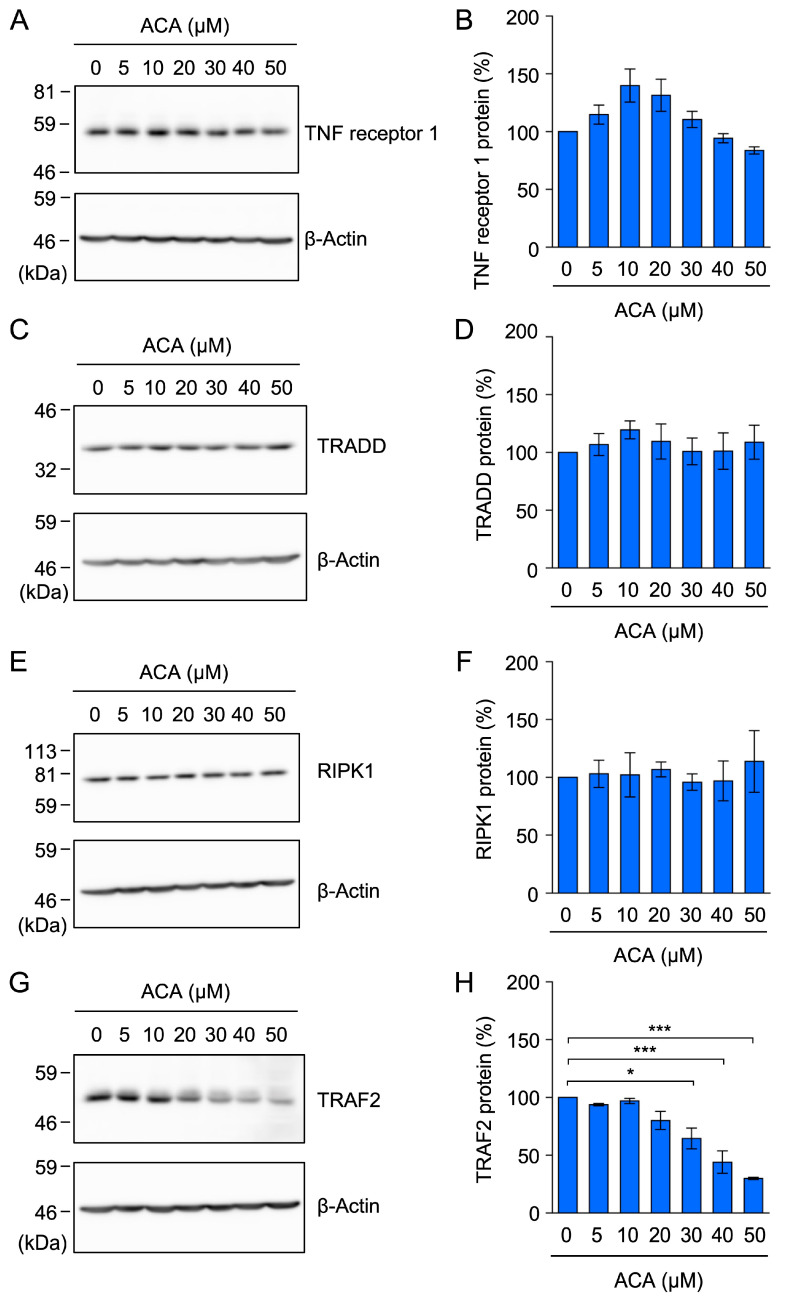
ACA did not affect the expression of the TNF receptor 1, TRADD, or RIPK1 protein, but reduced the expression of the TRAF2 protein in A549 cells. (**A**–**H**) A549 cells were incubated with the indicated concentrations of ACA for 1 h. Cytoplasmic fractions were prepared as cell lysates using 1% Triton X-100. A Western blotting analysis of cell lysates was then performed. Representative blots for the expression of TNF receptor 1, TRADD, RIPK1, TRAF2, and β-actin are shown (**A**,**C**,**E**,**G**). The amount of the β-actin protein was used to normalize the amount of each test protein. TNF receptor 1 protein (%), TRADD protein (%), RIPK1 protein (%), and TRAF2 protein (%) are shown as the mean ± standard error of three independent experiments (**B**,**D**,**F**,**H**). * *p* < 0.05 and *** *p* < 0.001. Original blots are shown in Figures S5–S20.

TNF receptor 1 is responsible for the TNF-α-induced NF-κB signaling pathway in A549 cells because these cells express TNF receptor 1, but not TNF receptor 2 [40]. When A549 cells were exposed to ACA for 1 h, the amount of the TNF receptor 1 protein in cell lysates was not markedly decreased by ACA (Figure 6A,B).

In response to a TNF-α stimulation, TNF receptor 1 has been shown to form a membrane-bound complex to trigger the NF-κB signaling pathway by recruiting TRADD, RIPK1, and TRAF2 [12,13,14]. To investigate the effects of ACA on the expression of components of the TNF receptor 1 complex, A549 cells were incubated with ACA for 1 h, cytoplasmic fractions were prepared as cell lysates using 1% Triton X-100, and these lysates were analyzed by Western blotting. ACA at concentrations up to 50 µM did not markedly affect TRADD or RIPK1 protein expression (Figure 6C–F). In contrast, ACA at 30–50 µM reduced TRAF2 protein expression (Figure 6G,H). These results showed that among the components of the TNF receptor 1 complex, ACA selectively reduced TRAF2 protein expression.

### 2.6. ACA Decreased the Amount of the TRAF2 Protein in Whole-Cell Lysates of A549 Cells

**Figure 7:**
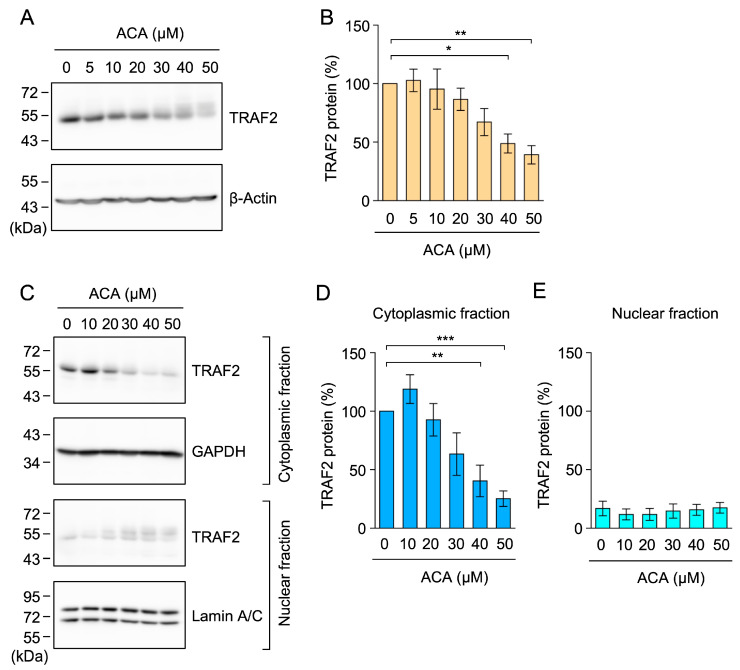
ACA decreased the amount of the TRAF2 protein in whole-cell lysates of A549 cells. (**A**,**B**) A549 cells were incubated with the indicated concentrations of ACA for 1 h. Western blotting was conducted to analyze whole-cell lysates prepared using 1% SDS. Representative blots for the expression of TRAF2 and β-actin are shown (**A**). The amount of the β-actin protein was used to normalize the amount of the TRAF2 protein. TRAF2 protein (%) is shown as the mean ± standard error of four independent experiments (**B**). (**C**–**E**) A549 cells were incubated with the indicated concentrations of ACA for 1 h. Western blotting was conducted to analyze cytoplasmic and nuclear fractions. Representative blots for the expression of TRAF2, GAPDH, and lamin A/C are shown (**C**). The amount of the GAPDH and lamin A/C proteins was used to normalize the amount of TRAF2 protein. TRAF2 protein (%) in cytoplasmic fractions (**D**) and nuclear fractions (**E**) is shown as the mean ± standard error of three independent experiments. * *p* < 0.05, ** *p* < 0.01, and *** *p* < 0.001. Original blots are shown in Figures S21–S32.

To further confirm the effects of ACA on TRAF2 protein expression, A549 cells were incubated with ACA for 1 h, and whole-cell lysates were prepared using 1% SDS lysis buffer. In whole-cell lysates, ACA reduced TRAF2 protein expression in a dose-dependent manner (Figure 7A,B). To clarify whether ACA promoted the translocation of the TRAF2 protein from cytoplasmic fractions to nuclear fractions, we lysed A549 cells using 1% Triton X-100 lysis buffer and separated them into cytoplasmic fractions and nuclear fractions containing nuclei and insoluble materials. A treatment with ACA for 1 h did not significantly affect the amount of the TRAF2 protein in nuclear fractions, while it decreased that in cytoplasmic fractions (Figure 7C–E). Therefore, these results showed that ACA promoted the downregulation of TRAF2 protein expression in whole-cell lysates.

### 2.7. ACA Promoted the Proteasomal Degradation of TRAF2 in A549 Cells

**Figure 8:**
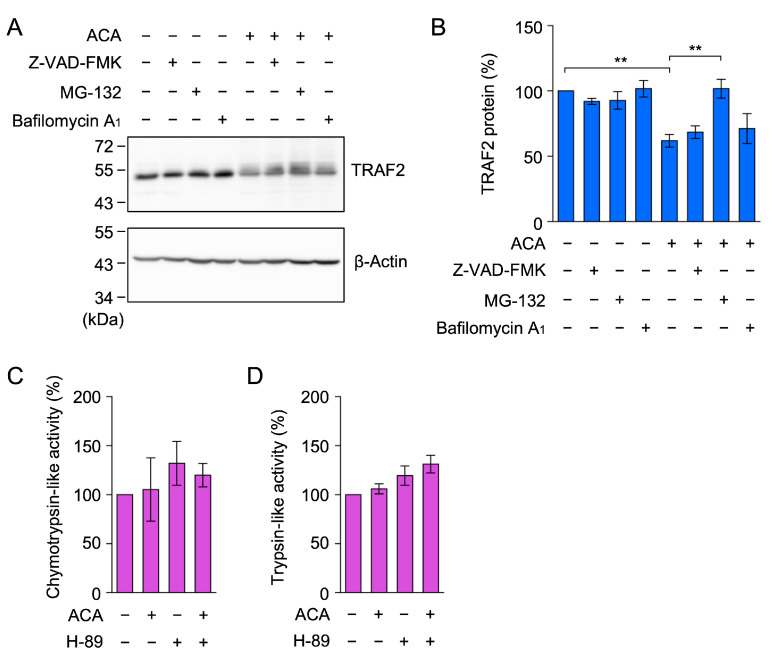
ACA promoted the proteasomal degradation of TRAF2 in A549 cells. (**A**,**B**) A549 cells were preincubated with (+) or without (−) Z-VAD-FMK, MG-132, and bafilomycin A_1_ for 1 h, and were then incubated with (+) or without (−) ACA for 1 h (40 µM) in the presence of Z-VAD-FMK (20 µM), MG-132 (20 µM), and bafilomycin A_1_ (100 nM). Western blotting was used to analyze whole-cell lysates. Representative blots for the expression of TRAF2 and β-actin are shown (**A**). The amount of the β-actin protein was used to normalize the amount of the TRAF2 protein. TRAF2 protein (%) is shown as the mean ± standard error of three independent experiments (**B**). ** *p* < 0.01. Original blots are shown in Figures S33–S36. (**C**,**D**) A549 cells were preincubated with (+) or without (−) H-89 (10 µM) for 1 h, and were then incubated with (+) or without (−) ACA (50 µM) for 1 h at the indicated concentrations. Chymotrypsin-like activity (%) (**C**) and trypsin-like activity (%) (**D**) are shown as the mean ± standard error of three independent experiments. No significant differences were observed in Figure 8C,D.

We previously reported that the treatment with the translation inhibitor cycloheximide for 1 h did not decrease TRAF2 protein expression in A549 cells [40]. Based on this finding, we herein investigated whether ACA promoted the proteolytic degradation of the TRAF2 protein using cellular proteolysis inhibitors, i.e., the caspase inhibitor Z-VAD-FMK, the proteasome inhibitor MG-132, and the vacuolar-type H^+^-ATPase inhibitor bafilomycin A_1_, which inhibits lysosomal degradation. MG-132 selectively reversed the reduction in the TRAF2 protein in ACA-treated A549 cells, whereas Z-VAD-FMK and bafilomycin A_1_ did not (Figure 8A,B).

A previous study reported that ACA increased proteasome activity by activating protein kinase A in rat pheochromocytoma PC-12 cells [41]. Therefore, we investigated whether ACA increased proteasome activity in A549 cells. A549 cells were pretreated with or without the protein kinase A inhibitor H-89 for 1 h, and were then treated with ACA for 1 h. In contrast to PC-12 cells, chymotrypsin-like activity and trypsin-like activity were not markedly affected by the presence of ACA and/or H-89 in A549 cells (Figure 8C,D). These results indicate that ACA did not increase the global proteasome activity in A549 cells. Taken together, it appears that ACA affects the ubiquitination process of the TRAF2 protein prior to proteasomal degradation.

### 2.8. ACA Inhibited the TNF-α-Induced NF-κB Signaling Pathway in HT-1080 Cells

**Figure 9:**
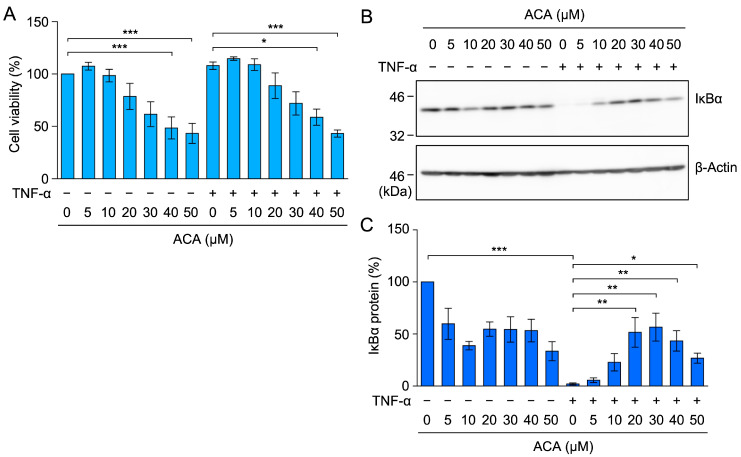
ACA inhibited TNF-α-induced IκBα degradation in HT-1080 cells. (**A**) HT-1080 cells were preincubated with ACA for 1 h, and were then incubated with (+) or without (−) TNF-α (2.5 ng/mL) for 2 h in the presence (+) or absence (−) of ACA. Cell viability (%) is shown as the mean ± standard error of three independent experiments. (**B**,**C**) HT-1080 cells were preincubated with ACA for 1 h, and were then incubated with (+) or without (−) TNF-α (2.5 ng/mL) for 15 min in the presence (+) or absence (−) of ACA. Western blotting was performed to analyze whole-cell lysates. Representative blots for the expression of IκBα and β-actin are shown (**B**). The amount of the IκBα protein was normalized to the amount of the β-actin protein. IκBα protein (%) is shown as the mean ± standard error of three independent experiments (**C**). * *p* < 0.05, ** *p* < 0.01, and *** *p* < 0.001. Original blots are shown in Figures S37–S40.

Human fibrosarcoma HT-1080 cells are responsive to TNF-α, which induces the activation of the NF-κB signaling pathway [42]. HT-1080 cells were used as an additional cell line to generalize the inhibitory activity of ACA on the TNF-α-dependent NF-κB signaling pathway. ACA decreased the viability of HT-1080 cells during a 3-h incubation at concentrations higher than 30 µM (Figure 9A). HT-1080 cells were treated with ACA for 1 h and were then stimulated with TNF-α for 15 min. TNF-α strongly induced the degradation of the IκBα protein (Figure 9B,C). ACA inhibited TNF-α-induced IκBα degradation at concentrations higher than 20 µM (Figure 9B,C). These results indicate that ACA inhibited the TNF-α-induced NF-κB signaling pathway in HT-1080 cells.

### 2.9. ACA Downregulated TRAF2 Protein Expression in HT-1080 Cells

**Figure 10:**
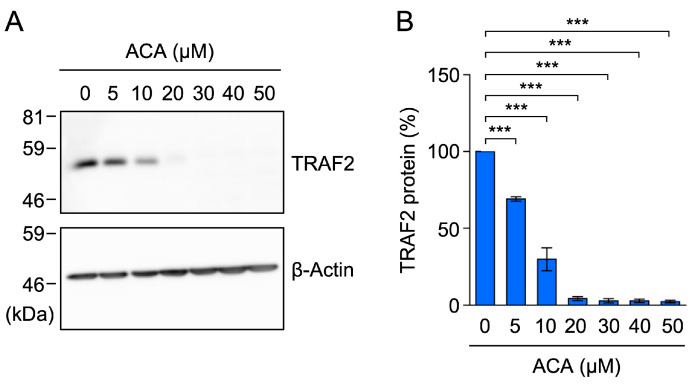
ACA downregulated the expression of TRAF2 in HT-1080 cells. (**A**,**B**) HT-1080 cells were incubated with ACA for 1 h. Western blotting was performed to analyze cell lysates. Representative blots for the expression of TRAF2 and β-actin are shown (**A**). The amount of the β-actin protein was used to normalize the amount of the TRAF2 protein. TRAF2 protein (%) is shown as the mean ± standard error of three independent experiments (**B**). *** *p* < 0.001. Original blots are shown in Figures S41–S44.

Further investigations were performed to examine the effects of ACA on TRAF2 protein expression in HT-1080 cells. ACA markedly reduced TRAF2 protein expression at concentrations higher than 10 µM (Figure 10A,B). These results show that ACA reduced TRAF2 protein expression in HT-1080 cells at concentrations that did not affect cell viability.

## 3. Discussion

**Figure 11:**
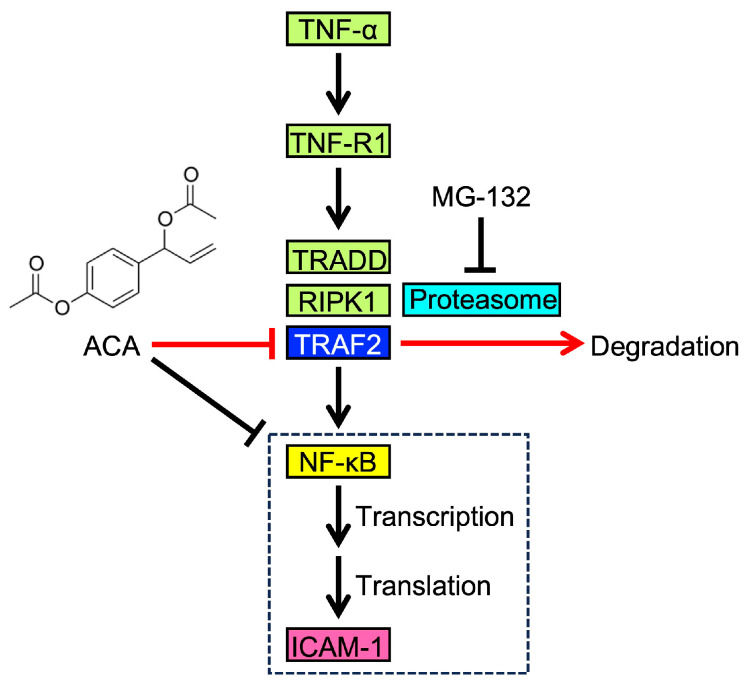
Proposed mechanism by which ACA affects the TNF-α-induced NF-κB pathway and ICAM-1 expression. Upon binding to TNF-α, TNF-R1 recruits TRADD, RIPK1, and TRAF2, which are required for the activation of NF-κB. NF-κB translocates to the nucleus and promotes the transcription of ICAM-1 as one of its target genes. ICAM-1 mRNA is then used to synthesize its translated product, which is transported to the cell surface. As an initial step in TNF-α-induced ICAM-1 expression, ACA downregulates TRAF2 expression through proteasome-dependent degradation. Luciferase reporter and quantitative PCR assays suggest that ACA inhibits the transcriptional process of NF-κB in addition to its effects on TRAF2.

ACA has been reported to inhibit the degradation of the IκBα protein and the subsequent NF-κB signaling pathway [23,24,26,27,28,29,30,31,32,33]; however, the underlying mechanisms remain unclear. In the present study, we found that ACA selectively downregulated TRAF2 protein expression without affecting the TNF receptor 1, TRADD, or RIPK1 protein. We also showed that ACA promoted the proteasomal degradation of the TRAF2 protein. These results reveal a novel biological activity for ACA, namely, the targeting of the TRAF2 protein. The potential mechanism by which ACA affects the TNF-α-induced NF-κB signaling pathway is shown in Figure 11.

ACA was previously shown to decrease cell viability and induce cell death in human breast cancer cells, mouse Ehrlich ascites tumor cells, and human leukemia HL-60 cells [35,36,37,38,39]. ACA increased ROS generation in cancer cells [35,36,37,38,39]. Furthermore, NAC or glutathione ethyl ester reversed the decrease in cell viability and the increase in cell death induced by ACA [35,36,37,38,39]. Consistent with these findings, ACA decreased cellular glutathione levels [35,38,39]. Therefore, these studies indicate that ACA inhibited tumor cell viability and induced cell death by affecting the intracellular ROS level.

ROS inactivate or activate the NF-κB signaling pathway in a cell context-dependent manner [43,44]. NAC and glutathione can be used as radical scavengers to inhibit the generation of ROS. In the NF-κB signaling pathway, many natural and synthetic compounds are known to bind the cysteine residues of essential proteins (i.e., IκB kinases and NF-κB subunits) and thereby inhibit their biological activities [34,45,46,47]. In addition to radical scavengers, NAC and glutathione can also be used to antagonize the binding of thiol-reactive compounds to their target proteins. To investigate whether the inhibitor activity of ACA is attributed to its binding of cellular proteins in a thiol-dependent manner or its promotion of ROS generation, NAC and glutathione were used as blocking agents. However, the addition of NAC or glutathione did not prevent TNF-α-induced ICAM-1 expression. Therefore, ACA appeared to inhibit NF-κB-dependent gene expression in a manner independent of the intracellular ROS levels or direct binding to cysteine residues of target proteins.

The NF-κB signaling pathway is activated by various stimuli, including pro-inflammatory cytokines and Toll-like receptor (TLR) ligands [2,48,49]. These stimuli trigger distinct sets of adaptor proteins required for the activation of the IκB kinase complex [12,13,14,48,49]. ACA has been reported to inhibit LPS (the TLR4 ligand)-induced IκB degradation in mouse RAW264.7 cells [23,26], LPS-induced IκB degradation in mouse bone marrow-derived macrophages and human monocytic THP-1 cells [28], and RANKL-induced IκB phosphorylation and degradation in RAW264.7 cells [27]. In the present study, we showed that ACA inhibited TNF-α-induced IκB degradation in A549 cells and HT-1080 cells. ACA also inhibited constitutively activated IκB phosphorylation and degradation in various human cancer cell lines [29,30]. The phosphorylation of IκB kinase α and β was decreased by ACA in human oral cancer cells [33]. Collectively, these findings suggest that ACA prevents the early process required for the activation of IκB kinase. However, it is currently unclear whether ACA inhibits a common step upstream of the activation of IκB kinase required for each signaling pathway.

Dose-dependent experiments were performed to examine the effects of ACA on ICAM-1 protein expression, ICAM-1 mRNA expression, ICAM-1 promoter-driven and NF-κB-dependent luciferase reporters, and TRAF2 protein expression. ACA almost completely inhibited TNF-α-induced ICAM-1 expression at the protein and mRNA levels at concentrations higher than 20 µM. Furthermore, ICAM-1 promoter-driven and NF-κB-dependent luciferase activities were decreased to unstimulated levels by ACA at concentrations higher than 30 µM. In the NF-κB pathway, ACA decreased TRAF2 protein expression and inhibited IκBα protein degradation in a dose-dependent manner at 20–50 µM. Therefore, ACA inhibited the NF-κB signaling pathway at the initial targets, while also suppressing downstream processes involved in transcription (Figure 11).

We demonstrated that a treatment with the translation inhibitor cycloheximide for 1 h did not affect TRAF2, TRADD, or RIPK1 protein amounts in A549 cells [40], indicating that these proteins are relatively stable in A549 cells. ACA selectively downregulated TRAF2 protein expression without affecting TNF receptor 1, TRADD, or RIPK1 protein amounts in cytoplasmic fractions. In whole-cell lysates prepared by 1% SDS lysis buffer, ACA decreased the amount of the TRAF2 protein. The presence of some TRAF2 protein in the nuclear fractions is consistent with previous findings showing the presence of TRAF2 in the cytoplasm and nucleus in different cell types [50,51,52] and the sequestration of TRAF2 in stress granules as insoluble materials [53,54].

The TRAF2 protein is ubiquitinated and subsequently undergoes proteasomal degradation [55,56,57] and lysosomal degradation [58,59]. The decrease in TRAF2 protein expression induced by ACA was reversed by the proteasome inhibitor MG-132, but not by the vacuolar-type H^+^-ATPase inhibitor bafilomycin A_1_, which blocks lysosomal proteolysis. ACA has been shown to increase proteasome activity by activating protein kinase A in rat pheochromocytoma PC12 cells [41]. In contrast to PC-12 cells, chymotrypsin-like and trypsin-like proteasome activities were not increased by ACA in the presence or absence of the protein kinase A inhibitor H-89 in A549 cells. Therefore, the ACA-induced downregulation of TRAF2 protein expression was not due to a global increase in proteasome activity. Thus, it seems most likely that ACA affects the ubiquitination process of the TRAF2 protein.

The TRAF family of proteins consists of seven members (TRAF1 to TRAF7) in mammals, and possesses conserved domains: the N-terminal RING domain (except TRAF1), the zinc finger domain, and the TRAF domain (except TRAF7) [60,61]. The RING domain is characterized by E3 ubiquitin ligases and is responsible for catalytic activity, while the TRAF domain is divided into the TRAF-N domain and the TRAF-C domain, which are associated with receptors and adaptor proteins [60]. The TRAF2 protein has been shown to undergo ubiquitination mediated by Siah2, c-IAP1, and FBXL2 as the E3 ubiquitin ligase or its subunit [55,56,62,63], whereas ubiquitin-specific proteinase 19 (USP19) and USP48, as the deubiquitinating enzymes, stabilize the TRAF2 protein [57,64]. Therefore, in addition to constitutive transcription and translation, the cellular level of the TRAF2 protein is regulated by the overall balance of multiple E3 ubiquitin ligases and deubiquitinating enzymes. Further experiments are needed to clarify whether ACA promotes the ubiquitination of the TRAF2 protein.

Previous studies reported that ACA exhibited anti-inflammatory activities toward allergic and anaphylactic reactions, as well as anticancer activity toward various tumor cells, such as colorectal carcinoma cells, multiple myeloma cells, and breast carcinoma cells [21,22]. Regarding TRAF2, somatic mutations in TRAF2 have been detected in diffuse large B-cell lymphoma [65]; the knockdown of TRAF2 suppressed the proliferation of human breast carcinoma cells [66,67], and liquidambaric acid inhibited colon carcinoma cells by directly targeting TRAF2 [68]. Collectively, these findings indicate the potential of TRAF2 as a target for anticancer drugs. The ablation of TRAF2 has been shown to reduce the TNF-dependent cytotoxicity threshold, thereby augmenting the susceptibility of tumors to immunotherapy mediated by CD8^+^ T cells [69]. Therefore, the downregulation of TRAF2 expression by ACA is promising as an anticancer agent.

## 4. Materials and Methods

### 4.1. Cell Culture

Human lung adenocarcinoma A549 cells (JCRB0076) and human fibrosarcoma HT-1080 cells (JCRB9113) were obtained from the National Institutes of Biomedical Innovation, Health and Nutrition JCRB Cell Bank (Osaka, Japan). Both cell lines were cultured with an RPMI 1640 medium (Thermo Fisher Scientific, Gland Island, NY, USA) containing fetal calf serum (Sigma-Aldrich, St. Louis, MO, USA), which was inactivated by a treatment at 56 °C for 30 min, and penicillin–streptomycin mixed solution (stabilized) (Nacalai Tesque, Kyoto, Japan).

### 4.2. Reagents

ACA (LKT Laboratories, Inc., St. Paul, MN, USA), bafilomycin A_1_ (Cayman Chemical, Ann Arbor, MI, USA), glutathione (Nacalai Tesque), H-89 (Abcam, Waltham, MA, USA), NAC (Nacalai Tesque), Z-Leu-Leu-Leu-H (MG-132) (Peptide Institute, Osaka, Japan), and Z-Val-Ala-Asp(OMe)-CH_2_F (Z-VAD-FMK) (Peptide Institute, Osaka, Japan) were obtained from commercial suppliers. Recombinant human TNF-α was provided by Dainippon Pharmaceutical Co., Ltd. (Osaka, Japan).

### 4.3. Antibodies

Primary mouse antibodies reactive to β-actin (A5441; Sigma-Aldrich, St. Louis, MO, USA), GAPDH (sc-32233; Santa Cruz Biotechnology, Dallas, TX, USA), γ1-actin (012-27823; FUJIFILM Wako Pure Chemical Corporation, Osaka, Japan), ICAM-1 (clone number: 15.2) (C167; Leinco Technologies, Inc., St. Louis, MO, USA), IκBα (610690; BD Biosciences, San Jose, CA, USA), lamin A/C (sc-376248; Santa Cruz Biotechnology), RIPK1 (610458; BD Biosciences), TNF-R1 (sc-8436; Santa Cruz Biotechnology), TRADD (610572; BD Biosciences), and TRAF2 (sc-136999; Santa Cruz Biotechnology), and a control mouse antibody (clone number: MOPC-21) (400101; BioLegend, San Diego, CA, USA), were used. A peroxidase-conjugated anti-mouse IgG(H+L) antibody (115-035-146; Jackson ImmunoResearch Laboratories, West Grove, PA, USA) and a phycoerythrin-conjugated anti-mouse IgG antibody (115-115-164; Jackson ImmunoResearch) were used as secondary antibodies.

### 4.4. Plasmids

A pGL4.22[luc2CP/Puro] vector encoding the ICAM-1 promoter (−1604 to +40) [70], an NF-κB-responsive firefly luciferase reporter vector [63], and a pCR3 expression vector containing cytomegalovirus promoter-driven *Renilla* luciferase were used in reporter assays. The NF-κB-responsive firefly luciferase reporter is under the control of two copies of κB sequences from the Igκ enhancer [71].

### 4.5. Measurement of Cell Viability

Cell viability was evaluated by using the MTT assay as previously described [72,73]. Absorbance at 570 nm was measured by an iMark^TM^ microplate reader (Bio-Rad Laboratories, Hercules, CA, USA).

### 4.6. Measurement of ICAM-1 Expression

ICAM-1 expression was evaluated using cell-ELISA as previously described [72,73]. A549 cells were treated with an anti-ICAM-1 antibody (clone number: 15.2) as the primary antibody and a peroxidase-conjugated anti-mouse IgG antibody as the secondary antibody. Absorbance at 450 nm was measured by the iMark^TM^ microplate reader.

### 4.7. Flow Cytometry

Flow cytometry was performed as previously described [74]. A549 cells were stained with the anti-ICAM-1 antibody (clone number: 15.2) or an isotype control antibody (clone number: MOPC-12) and then with a phycoerythrin-labeled secondary antibody, followed by an analysis using FACSCalibur (BD Biosciences) and FlowJo software version 8.5.1 (Tomy Digital Biology, Tokyo, Japan).

### 4.8. Measurement of mRNA Expression

Sepasol^®^-RNA I Super G (Nacalai Tesque), ReverTra Ace^®^ (TOYOBO, Osaka, Japan), and TB Green^®^ Premix Ex Taq^TM^ II (Tli RNase H Plus) (Takara Bio, Kusatsu, Japan) were obtained from commercial suppliers. The primers for human ICAM-1 (148 bp) [75] and β-actin (143 bp) [76] were previously described. Real-time PCR conditions using Thermal Cycler Dice^®^ Real-Time System Lite (Takara Bio) were described in another study [72,73]. The amount of mRNA expression was calculated based on primer-specific standard curves.

### 4.9. Luciferase Reporter Assay

HilyMax transfection reagent (Dojindo Laboratories, Kumamoto, Japan) was used for the transfection of luciferase reporter plasmids. The preparation of cell lysates and the measurement of firefly and *Renilla* luciferase activities were performed as previously reported [72]. Relative light units were measured by Lumitester C-110 (Kikkoman Biochemifa, Tokyo, Japan). Firefly luciferase activity was normalized by *Renilla* luciferase activity.

### 4.10. Preparation of Cell Lysates and Whole-Cell Lysates

Cells were washed with PBS and lyzed on ice for 15 min with Triton X-100 lysis buffer, consisting of 50 mM Tris-HCl (pH 7.4), 1% Triton X-100, 2 mM dithiothreitol, 2 mM sodium vanadate, and cOmplete^TM^ protease inhibitor cocktail (Sigma-Aldrich). After centrifugation (15,300× *g*, 5 min), cytoplasmic fractions were collected as supernatants. Precipitates were washed with Triton X-100 lysis buffer and further treated with sonication to prepare nuclear fractions. To prepare whole-cell lysates, cells were washed with PBS and then treated with Triton X-100 lysis buffer or SDS lysis buffer, consisting of 50 mM Tris-HCl (pH 7.4), 1% SDS, 2 mM dithiothreitol, 2 mM sodium vanadate, and 2 mM PMSF, and further treated with sonication. Protein concentrations were measured by Protein Assay CBB solution (5×) (Nacalai Tesque).

### 4.11. Western Blotting

Cell lysates or whole-cell lysates were analyzed by Western blotting as previously described [72,73]. Images of Western blots were captured using an Amersham Imager 680 (GE Healthcare Japan, Tokyo, Japan). Membranes were treated with a stripping solution (Fujifilm Wako Pure Chemical Corporation) and were then subjected to additional Western blotting using anti-β-actin, γ1-actin, GAPDH, and lamin A/C antibodies. The intensity of protein bands was measured using ImageQuantTL software toolbox version 7.0.1.0 (GE Healthcare Japan). The amounts of target proteins were normalized by that of β-actin, γ1-actin, GAPDH, and lamin A/C.

### 4.12. Proteasome Assay

Proteasome activities were measured using the proteasome-Glo^TM^ Chymotrypsin-Like Cell-based Assay (G8660) (Promega, Madison, MI, USA) and the Proteasome-Glo^TM^ Trypsin-Like Cell-based Assay (G8760) (Promega). Cells were washed twice with PBS and were then suspended in PBS. The cell suspension was incubated with the proteasome assay mixture for 10 min. Relative light units were measured using Lumitester C-110.

### 4.13. Statistical Analysis

Experiments were independently repeated at least three times for a quantitative analysis. The number of experiments was described in each figure caption. Means ± standard errors were calculated from at least three independent experiments. Data were analyzed statistically by a one-way ANOVA followed by Tukey’s test (KaleidaGraph software version 4.5.1.; Hulinks, Tokyo, Japan).

## 5. Conclusions

ACA is a major ingredient of plants in the Zingiberaceae family used as a spice and in folk medicine. The present results revealed the novel biological activity of ACA, which downregulates the expression of the TRAF2 protein via the ubiquitin–proteasome system. TRAF2 is one of the components of the TNF receptor 1 complex and regulates the NF-κB signaling pathway. Previous studies showed that ACA inhibited the NF-κB signaling pathway induced by various inflammatory stimuli, and the constitutive activation of NF-κB in in vitro cancer cell lines in in vivo tumor models. These findings indicate the potential of ACA as an anti-inflammatory and anticancer drug. Further studies are needed to elucidate the comprehensive molecular mechanisms by which ACA inhibits the NF-κB signaling pathway induced by various agents, including pro-inflammatory cytokines and the TLR ligands.

## Data Availability

Data will be made available upon reasonable request.

## Supplementary Materials

The following supporting information can be downloaded at: www.mdpi.com/article/10.3390/molecules30061243/s1, Figure S1: Original blots in Figure 5B; Figure S2: Original blots (1) in Figure 5C; Figure S3: Original blots (2) in Figure 5C; Figure S4: Original blots (3) in Figure 5C; Figure S5: Original blots in Figure 6A; Figure S6: Original blots (1) in Figure 6B; Figure S7: Original blots (2) in Figure 6B; Figure S8: Original blots (3) in Figure 6B; Figure S9: Original blots in Figure 6C; Figure S10: Original blots (1) in Figure 6D; Figure S11: Original blots (2) in Figure 6D; Figure S12: Original blots (3) in Figure 6D; Figure S13: Original blots in Figure 6E; Figure S14: Original blots (1) in Figure 6F; Figure S15: Original blots (2) in Figure 6F; Figure S16: Original blots (3) in Figure 6F; Figure S17: Original blots in Figure 6G; Figure S18: Original blots (1) in Figure 6H; Figure S19: Original blots (2) in Figure 6H; Figure S20: Original blots (3) in Figure 6H; Figure S21: Original blots in Figure 7A; Figure S22: Original blots (1) in Figure 7B; Figure S23: Original blots (3) in Figure 7B; Figure S24: Original blots (3) in Figure 7B; Figure S25: Original blots (4) in Figure 7B; Figure S26: Original blots in Figure 7C; Figure S27: Original blots (1) in Figure 7D; Figure S28: Original blots (2) in Figure 7D; Figure S29: Original blots (3) in Figure 7D; Figure S30: Original blots (1) in Figure 7E; Figure S31: Original blots (2) in Figure 7E; Figure S32: Original blots (3) in Figure 7E; Figure S33: Original blots in Figure 8A; Figure S34: Original blots (1) in Figure 8B; Figure S35: Original blots (2) in Figure 8B; Figure S36: Original blots (3) in Figure 8B; Figure S37: Original blots in Figure 9B; Figure S38: Original blots (1) in Figure 9C; Figure S39: Original blots (2) in Figure 9C; Figure S40: Original blots (3) in Figure 9C; Figure S41: Original blots in Figure 10A; Figure S42: Original blots (1) in Figure 10B; Figure S43: Original blots (2) in Figure 10B; Figure S44: Original blots (3) in Figure 10B.

## References

[1] Shapouri-Moghaddam A., Mohammadian S., Vazini H., Taghadosi M., Esmaeili S.A., Mardani F., Seifi B., Mohammadi A., Afshari J.T., Sahebkar A. (2018). Macrophage plasticity, polarization, and function in health and disease. J. Cell Physiol..

[2] Wang T., He C. (2018). Pro-inflammatory cytokines: The link between obesity and osteoarthritis. Cytokine Growth Factor Rev..

[3] Bui T.M., Wiesolek H.L., Sumagin R. (2020). ICAM-1: A master regulator of cellular responses in inflammation, injury resolution, and tumorigenesis. J. Leukoc. Biol..

[4] Gerhardt T., Ley K. (2015). Monocyte trafficking across the vessel wall. Cardiovasc. Res..

[5] Vestweber D. (2015). How leukocytes cross the vascular endothelium. Nat. Rev. Immunol..

[6] Harjunpää H., Llort Asens M., Guenther C., Fagerholm S.C. (2019). Cell adhesion molecules and their roles and regulation in the immune and tumor microenvironment. Front. Immunol..

[7] Qiu Z., Wang Y., Zhang Z., Qin R., Peng Y., Tang W., Xi Y., Tian G., Zhang Y. (2022). Roles of intercellular cell adhesion molecule-1 (ICAM-1) in colorectal cancer: Expression, functions, prognosis, tumorigenesis, polymorphisms and therapeutic implications. Front. Oncol..

[8] Zhong L., Simard M.J., Huot J. (2018). Endothelial microRNAs regulating the NF-κB pathway and cell adhesion molecules during inflammation. FASEB J..

[9] Singh M., Thakur M., Mishra M., Yadav M., Vibhuti R., Memon A.M., Nagda G., Dwivedi V.P., Dakal T.C., Yadav V. (2021). Gene regulation of intracellular adhesion molecule-1 (ICAM-1): A molecule with multiple functions. Immunol. Lett..

[10] Wallach D. (2018). The tumor necrosis factor family: Family conventions and private idiosyncrasies. Cold Spring Harb. Perspect. Biol..

[11] Ham B., Fernandez M.C., D’Costa Z., Brodt P. (2016). The diverse roles of the TNF axis in cancer progression and metastasis. Trends Cancer Res..

[12] Kalliolias G.D., Ivashkiv L.B. (2016). TNF biology, pathogenic mechanisms and emerging therapeutic strategies. Nat. Rev. Rheumatol..

[13] Wajant H., Siegmund D. (2019). TNFR1 and TNFR2 in the control of the life and death balance of macrophages. Front. Cell Dev. Biol..

[14] Atretkhany K.S.N., Gogoleva V.S., Drutskaya M.S., Nedospasov S.A. (2020). Distinct modes of TNF signaling through its two receptors in health and disease. J. Leukoc. Biol..

[15] Scheidereit C. (2006). IκB kinase complexes: Gateways to NF-κB activation and transcription. Oncogene.

[16] Hayden M.S., Ghosh S. (2008). Shared principles in NF-κB signaling. Cell.

[17] Bhoj V.G., Chen Z.J. (2009). Ubiquitylation in innate and adaptive immunity. Nature.

[18] Sasaki K., Iwai K. (2015). Roles of linear ubiquitinylation, a crucial regulator of NF-κB and cell death, in the immune system. Immunol. Rev..

[19] Puar Y.R., Shanmugam M.K., Fan L., Arfuso F., Sethi G., Tergaonkar V. (2018). Evidence for the involvement of the master transcription factor NF-κB in cancer initiation and progression. Biomedicines.

[20] Guo Q., Jin Y., Chen X., Ye X., Shen X., Lin M., Zeng C., Zhou T., Zhang J. (2024). NF-κB in biology and targeted therapy: New insights and translational implications. Signal Transduct. Target Ther..

[21] Chouni A., Paul S. (2018). A review on phytochemical and pharmacological potential of Alpinia galanga. Pharmacogn. J..

[22] Kojima-Yuasa A., Matsui-Yuasa I. (2020). Pharmacological effects of 1′-acetoxychavicol acetate, a major constituent in the rhizomes of *Alpinia galanga* and *Alpinia conchigera*. J. Med. Food.

[23] Ohata T., Fukuda K., Murakami A., Ohigashi H., Sugimura T., Wakabayashi K. (1998). Inhibition by 1′-acetoxychavicol acetate of lipopolysaccharide- and interferon-γ-induced nitric oxide production through suppression of inducible nitric oxide synthase gene expression in RAW264 cells. Carcinogenesis.

[24] Murakami A., Matsumoto K., Koshimizu K., Ohigashi H. (2003). Effects of selected food factors with chemopreventive properties on combined lipopolysaccharide- and interferon-γ-induced IκB degradation in RAW264.7 macrophages. Cancer Lett..

[25] Ando S., Matsuda H., Morikawa T., Yoshikawa M. (2005). 1′S-1′-Acetoxychavicol acetate as a new type inhibitor of interferon-β production in lipopolysaccharide-activated mouse peritoneal macrophages. Bioorg. Med. Chem..

[26] Murakami A., Shigemori T., Ohigashi H. (2005). Zingiberaceous and citrus constituents, 1′-acetoxychavicol acetate, zerumbone, auraptene, and nobiletin, suppress lipopolysaccharide-induced cyclooxygenase-2 expression in RAW264.7 murine macrophages through different modes of action. J. Nutr..

[27] Ichikawa H., Murakami A., Aggarwal B.B. (2006). 1′-Acetoxychavicol acetate inhibits RANKL-induced osteoclastic differentiation of RAW 264.7 monocytic cells by suppressing nuclear factor-κB activation. Mol. Cancer Res..

[28] Ong G.H., Ori D., Kawasaki T., Kawai T. (2022). Inhibition of lipopolysaccharide-induced inflammatory responses by 1′-acetoxychavicol acetate. Genes Cells.

[29] Ichikawa H., Takada Y., Murakami A., Aggarwal B.B. (2005). Identification of a novel blocker of IκBα kinase that enhances cellular apoptosis and inhibits cellular invasion through suppression of NF-κB-regulated gene products. J. Immunol..

[30] Ito K., Nakazato T., Xian M.J., Yamada T., Hozumi N., Murakami A., Ohigashi H., Ikeda Y., Kizaki M. (2005). 1′-Acetoxychavicol acetate is a novel nuclear factor κB inhibitor with significant activity against multiple myeloma in vitro and in vivo. Cancer Res..

[31] Misawa T., Dodo K., Ishikawa M., Hashimoto Y., Sagawa M., Kizaki M., Aoyama H. (2015). Structure-activity relationships of benzhydrol derivatives based on 1′-acetoxychavicol acetate (ACA) and their inhibitory activities on multiple myeloma cell growth via inactivation of the NF-κB pathway. Bioorg. Med. Chem..

[32] Batra V., Syed Z., Gill J.N., Coburn M.A., Adegboyega P., DiGiovanni J., Mathis J.M., Shi R., Clifford J.L., Kleiner-Hancock H.E. (2012). Effects of the tropical ginger compound, 1′-acetoxychavicol acetate, against tumor promotion in K5.Stat3C transgenic mice. J. Exp. Clin. Cancer Res..

[33] In L.L., Arshad N.M., Ibrahim H., Azmi M.N., Awang K., Nagoor N.H. (2012). 1′-Acetoxychavicol acetate inhibits growth of human oral carcinoma xenograft in mice and potentiates cisplatin effect via proinflammatory microenvironment alterations. BMC Complement. Altern. Med..

[34] Kataoka T. (2009). Chemical biology of inflammatory cytokine signaling. J. Antibiot..

[35] Moffatt J., Kennedy D.O., Kojima A., Hasuma T., Yano Y., Otani S., Murakami A., Koshimizu K., Ohigashi H., Matsui-Yuasa I. (2002). Involvement of protein tyrosine phosphorylation and reduction of cellular sulfhydryl groups in cell death induced by 1′-acetoxychavicol acetate in Ehrlich ascites tumor cells. Chem. Biol. Interact..

[36] Campbell C.T., Prince M., Landry G.M., Kha V., Kleiner H.E. (2007). Pro-apoptotic effects of 1′-acetoxychavicol acetate in human breast carcinoma cells. Toxicol. Lett..

[37] Unahara Y., Kojima-Yuasa A., Higashida M., Kennedy D.O., Murakami A., Ohigashi H., Matsui-Yuasa I. (2007). Cellular thiol status-dependent inhibition of tumor cell growth via modulation of p27(kip1) translocation and retinoblastoma protein phosphorylation by 1′-acetoxychavicol acetate. Amino Acids.

[38] Higashida M., Xu S., Kojima-Yuasa A., Kennedy D.O., Murakami A., Ohigashi H., Matsui-Yuasa I. (2009). 1′-Acetoxychavicol acetate-induced cytotoxicity is accompanied by a rapid and drastic modulation of glutathione metabolism. Amino Acids.

[39] Xu S., Kojima-Yuasa A., Azuma H., Kennedy D.O., Konishi Y., Matsui-Yuasa I. (2010). Comparison of glutathione reductase activity and the intracellular glutathione reducing effects of 13 derivatives of 1′-acetoxychavicol acetate in Ehrlich ascites tumor cells. Chem. Biol. Interact..

[40] Ogura H., Tsukumo Y., Sugimoto H., Igarashi M., Nagai K., Kataoka T. (2008). Ectodomain shedding of TNF receptor 1 induced by protein synthesis inhibitors regulates TNF-α-mediated activation of NF-κB and caspase-8. Exp. Cell Res..

[41] Yaku K., Matsui-Yuasa I., Kojima-Yuasa A. (2018). 1′-Acetoxychavicol acetate increases proteasome activity by activating cAMP-PKA signaling. Planta Med..

[42] Fukuhara S., Tanigaki R., Kimura K., Kataoka T. (2018). Kujigamberol interferes with pro-inflammatory cytokine-induced expression of and *N*-glycan modifications to cell adhesion molecules at different stages in human umbilical vein endothelial cells. Int. Immunopharmacol..

[43] Nakajima S., Kitamura M. (2013). Bidirectional regulation of NF-κB by reactive oxygen species: A role of unfolded protein response. Free Radical Biol. Med..

[44] Lingappan K. (2018). NF-κB oxidative stress. Curr. Opin. Toxicol..

[45] Gilmore T.D., Herscovitch M. (2006). Inhibitors of NF-κB signaling: 785 and counting. Oncogene.

[46] Pande V., Sousa S.F., Ramos M.J. (2009). Direct covalent modification as a strategy to inhibit nuclear factor-kappa B. Curr. Med. Chem..

[47] Zhang J., Zhang R., Li W., Ma X.C., Qiu F., Sun C.P. (2023). IκB kinase β (IKKβ): Structure, transduction mechanism, biological function, and discovery of its inhibitors. Int. J. Biol. Sci..

[48] Kawai T., Akira S. (2011). Toll-like receptors and their crosstalk with other innate receptors in infection and immunity. Immunity.

[49] Cohen P. (2014). The TLR and IL-1 signaling network at a glance. J. Cell Sci..

[50] Min W., Bradley J.R., Galbraith J.J., Jones S.J., Ledgerwood E.C., Pober J.S. (1998). The N-terminal domains target TNF receptor-associated factor-2 to the nucleus and display transcriptional regulatory activity. J. Immunol..

[51] Thakar N.Y., Ovchinnikov D.A., Hastie M.L., Kobe B., Gorman J.J., Wolvetang E.J. (2015). TRAF2 recruitment via T61 in CD30 drives NFκB activation and enhances hESC survival and proliferation. Mol. Biol. Cell.

[52] El Hokayem J., Brittain IV G.C., Nawaz Z., Bethea J.R. (2017). Tumor necrosis factor receptor associated factors (TRAFs) 2 and 3 form a transcriptional complex with phosho-RNA polymerase II and p65 in CD40 ligand activated Neuro2a cells. Mol. Neurobiol..

[53] Kim W.J., Back S.H., Kim V., Ryu I., Jang S.K. (2005). Sequestration of TRAF2 into stress granules interrupts tumor necrosis factor signaling under stress conditions. Mol. Cell. Biol..

[54] Kim W.J., Kim J.H., Jang S.K. (2007). Anti-inflammatory lipid mediator 15d-PGJ2 inhibits translation through inactivation of eIF4A. EMBO J..

[55] Habelhah H., Frew I.J., Laine A., Janes P.W., Relaix F., Sassoon D., Bowtell D.D.L., Ronai Z. (2002). Stress-induced decrease in TRAF2 stability is mediated by Siah2. EMBO J..

[56] Li X., Yang Y., Ashwell J.D. (2002). TNF-RII and c-IAP1 mediate ubiquitination and degradation of TRAF2. Nature.

[57] Dhingra R., Rabinovich-Nikitin I., Rothman S., Guberman M., Gang H., Margulets V., Jassal D.S., Alagarsamy K.N., Dhingra S., Ripoll C.V. (2022). Proteasomal degradation of TRAF2 mediates mitochondrial dysfunction in doxorubicin-cardiomyopathy. Circulation.

[58] Vince J.E., Chau D., Callus B., Wong W.W.L., Hawkins C.J., Schneider P., McKinlay M., Benetatos C.A., Condon S.M., Chunduru S.K. (2008). TWEAK-FN14 signaling induces lysosomal degradation of a cIAP1-TRAF2 complex to sensitize tumor cells to TNFα. J. Cell Biol..

[59] Li L., Soetandyo N., Wang Q., Ye Y. (2009). The zinc finger protein A20 targets TRAF2 to the lysosomes for degradation. Biochim. Biophys. Acta.

[60] Park H.H. (2018). Structure of TRAF Family: Current understanding of receptor recognition. Front. Immunol..

[61] Arkee T., Bishop G.A. (2020). TRAF family molecules in T cells: Multiple receptors and functions. J. Leukoc. Biol..

[62] Chen B.B., Coon T.A., Glasser J.R., McVerry B.J., Zhao J., Zhao Y., Zou C., Ellis B., Sciurba F.C., Zhang Y. (2013). A combinatorial F box protein directed pathway controls TRAF adaptor stability to regulate inflammation. Nat. Immunol..

[63] Mallampalli R.K., Coon T.A., Glasser J.R., Wang C., Dunn S.R., Weathington N.M., Zhao J., Zou C., Zhao Y., Chen B.B. (2013). Targeting F box protein Fbxo3 to control cytokine-driven inflammation. J. Immunol..

[64] Li S., Wang D., Zhao J., Weathington N.M., Shang D., Zhao Y. (2018). The deubiquitinating enzyme USP48 stabilizes TRAF2 and reduces E-cadherin-mediated adherens junctions. FASEB J..

[65] Compagno M., Lim W.K., Grunn A., Nandula S.V., Brahmachary M., Shen Q., Bertoni F., Ponzoni M., Scandurra M., Califano A. (2009). Mutations of multiple genes cause deregulation of NF-κB in diffuse large B-cell lymphoma. Nature.

[66] Sun L.L., Wang J., Zhao Z.J., Liu N., Wang A.L., Ren H.Y., Yang F., Diao K.X., Fu W.N., Wan E.H. (2014). Suppressive role of miR-502-5p in breast cancer via downregulation of TRAF2. Oncol. Rep..

[67] Peramuhendige P., Marino S., Bishop R.T., de Ridder D., Khogeer A., Baldini I., Capulli M., Rucci N., Idris A.I. (2018). TRAF2 in osteotropic breast cancer cells enhances skeletal tumour growth and promotes osteolysis. Sci. Rep..

[68] Yan R., Zhu H., Huang P., Yang M., Shen M., Pan Y., Zhang C., Zhou X., Li H., Ke X. (2022). Liquidambaric acid inhibits Wnt/β-catenin signaling and colon cancer via targeting TNF receptor-associated factor 2. Cell Rep..

[69] Vredevoogd D.W., Kuilman T., Ligtenberg M.A., Boshuizen J., Stecker K.E., de Bruijn B., Krijgsman O., Huang X., Kenski J.C.N., Lacroix R. (2019). Augmenting immunotherapy impact by lowering tumor TNF cytotoxicity threshold. Cell.

[70] Vo N.T., Kusagawa E., Nakano K., Moriwaki C., Miyake Y., Haruyama S., Fukuhara S., Nguyen N.T., Dang P.H., Nguyen M.T.T. (2021). Biological evaluation of alkyl triphenylphosphonium ostruthin derivatives as potential anti-inflammatory agents targeting the nuclear factor κB signaling pathway in human lung adenocarcinoma A549 cells. BioChem.

[71] Dohrman A., Kataoka T., Cuenin S., Russell J.Q., Tschopp J., Budd R.C. (2005). Cellular FLIP (long form) regulates CD8^+^ T cell activation through caspase-8-dependent NF-κB activation. J. Immunol..

[72] Vu Q.V., Baba K., Sakaki S., Kawaguchi K., Hirano H., Osada H., Kataoka T. (2024). Alantolactone derivatives inhibit the tumor necrosis factor α-induced nuclear factor κB pathway by a different mechanism from alantolactone. Eur. J. Pharmacol..

[73] Vu Q.V., Vu N.T., Baba K., Sasaki S., Tamura R., Morimoto K., Hirano H., Osada H., Kataoka T. (2024). Porphyrin derivatives inhibit tumor necrosis factor α-induced gene expression and reduce the expression and increase the cross-linked forms of cellular components of the nuclear factor κB signaling pathway. Eur. J. Pharmacol..

[74] Vo N.T., Sasaki S., Miyake Y., Nguyen N.T., Dang P.H., Nguyen M.T.T., Kataoka T. (2021). α-Conidendrin inhibits the expression of intercellular adhesion molecule-1 induced by tumor necrosis factor-α in human lung adenocarcinoma A549 cells. Eur. J. Pharmacol..

[75] Wan M., Liu J., Ouyang X. (2015). Nucleotide-binding oligomerization domain 1 regulates *Porphyromonas gingivalis*-induced vascular cell adhesion molecule 1 and intercellular adhesion molecule 1 expression in endothelial cells through NF-κB pathway. J. Periodontal. Res..

[76] Zhang Y., Lian F., Zhu Y., Xia M., Wang Q., Ling W., Wang X.D. (2010). Cyanidin-3-*O*-*β*-glucoside inhibits LPS-induced expression of inflammatory mediators through decreasing IκBα phosphorylation in THP-1 cells. Inflamm. Res..

