# Supplementary material for: 1′-Acetoxychavicol Acetate Selectively Downregulates Tumor Necrosis Factor Receptor-Associated Factor 2 (TRAF2) Expression
Source: Molecules. 2025 Mar 10;30(6):1243. doi: 10.3390/molecules30061243 (PMC11945442; doi:10.3390/molecules30061243)

Figure S1: Original blots in Figure 5B

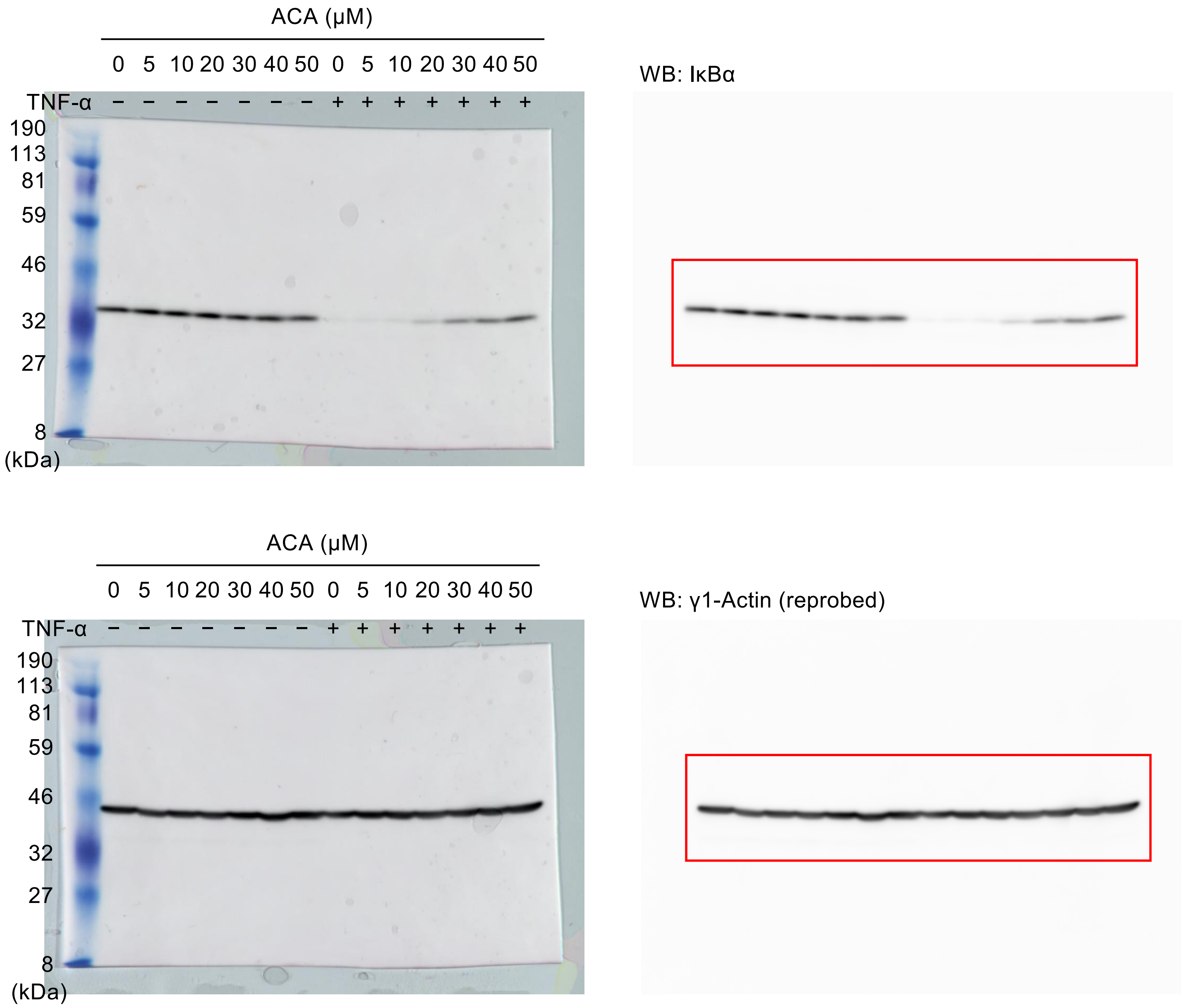

Figure S2: Original blots (1) in Figure 5C

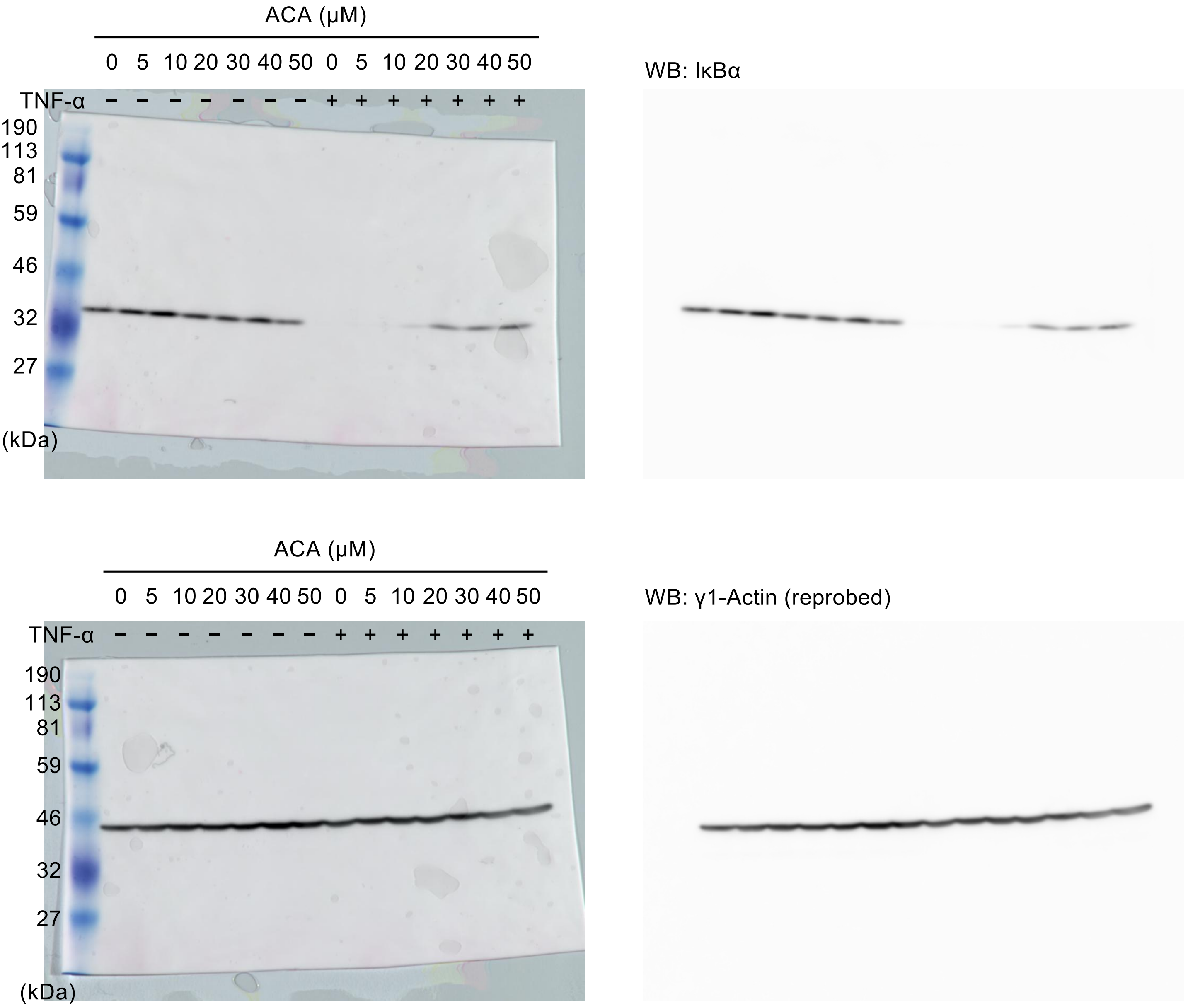

Figure S3: Original blots (2) in Figure 5C

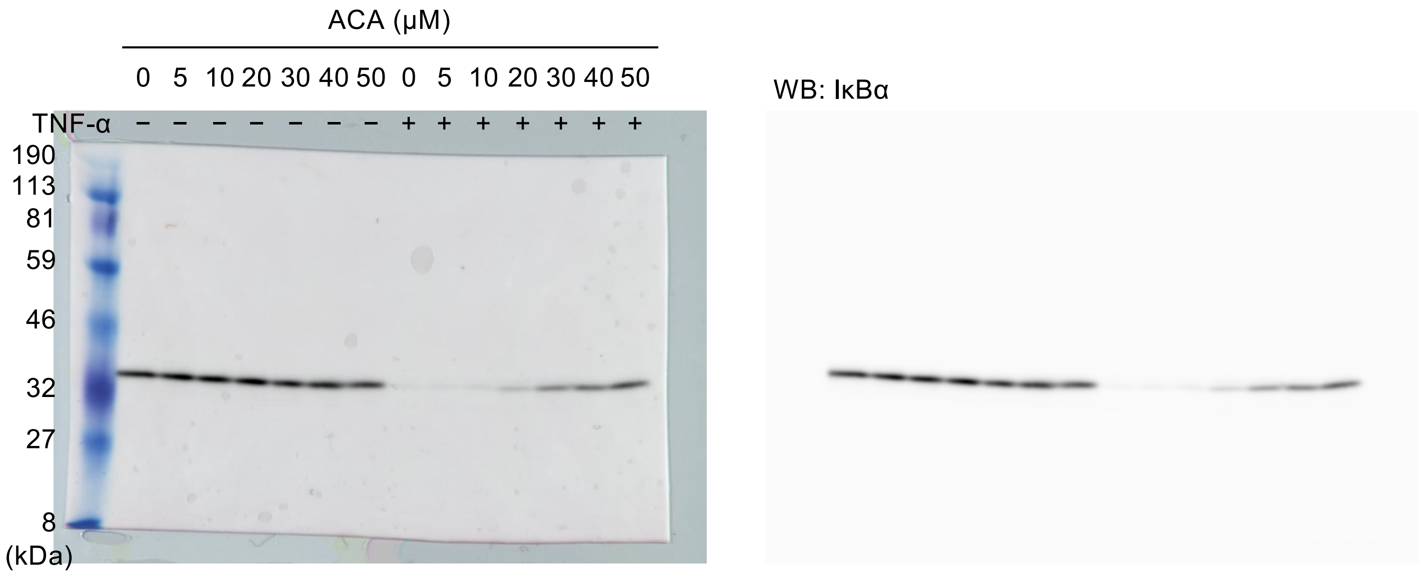

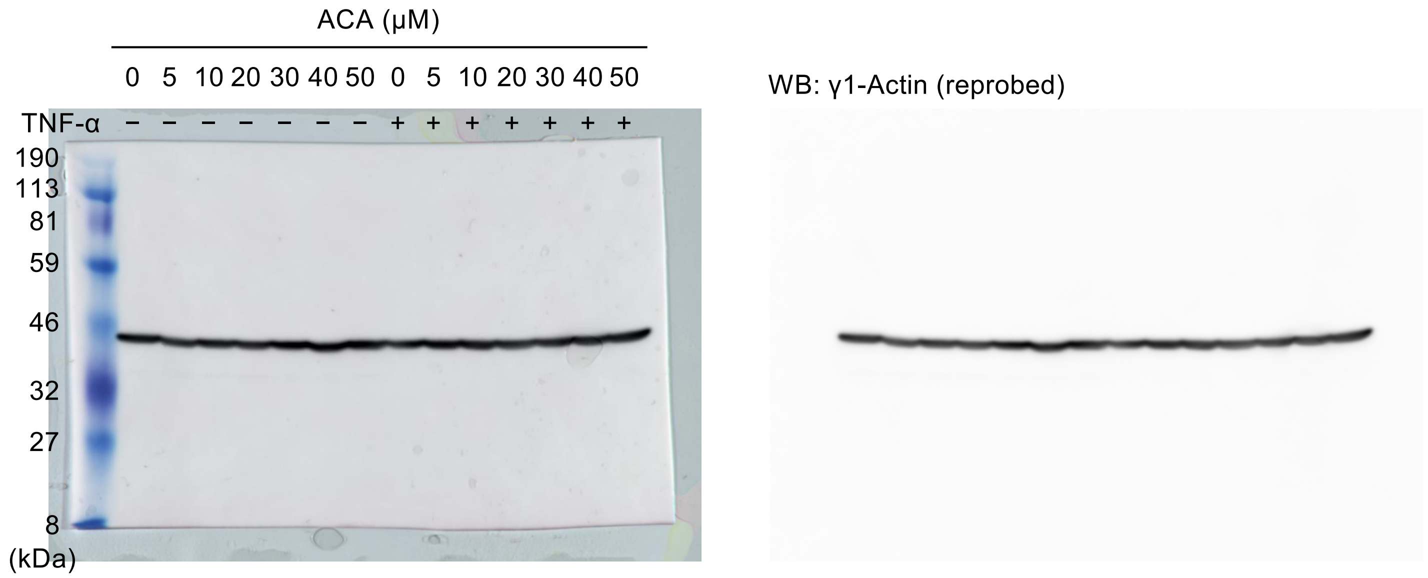

Figure S4: Original blots (3) in Figure 5C

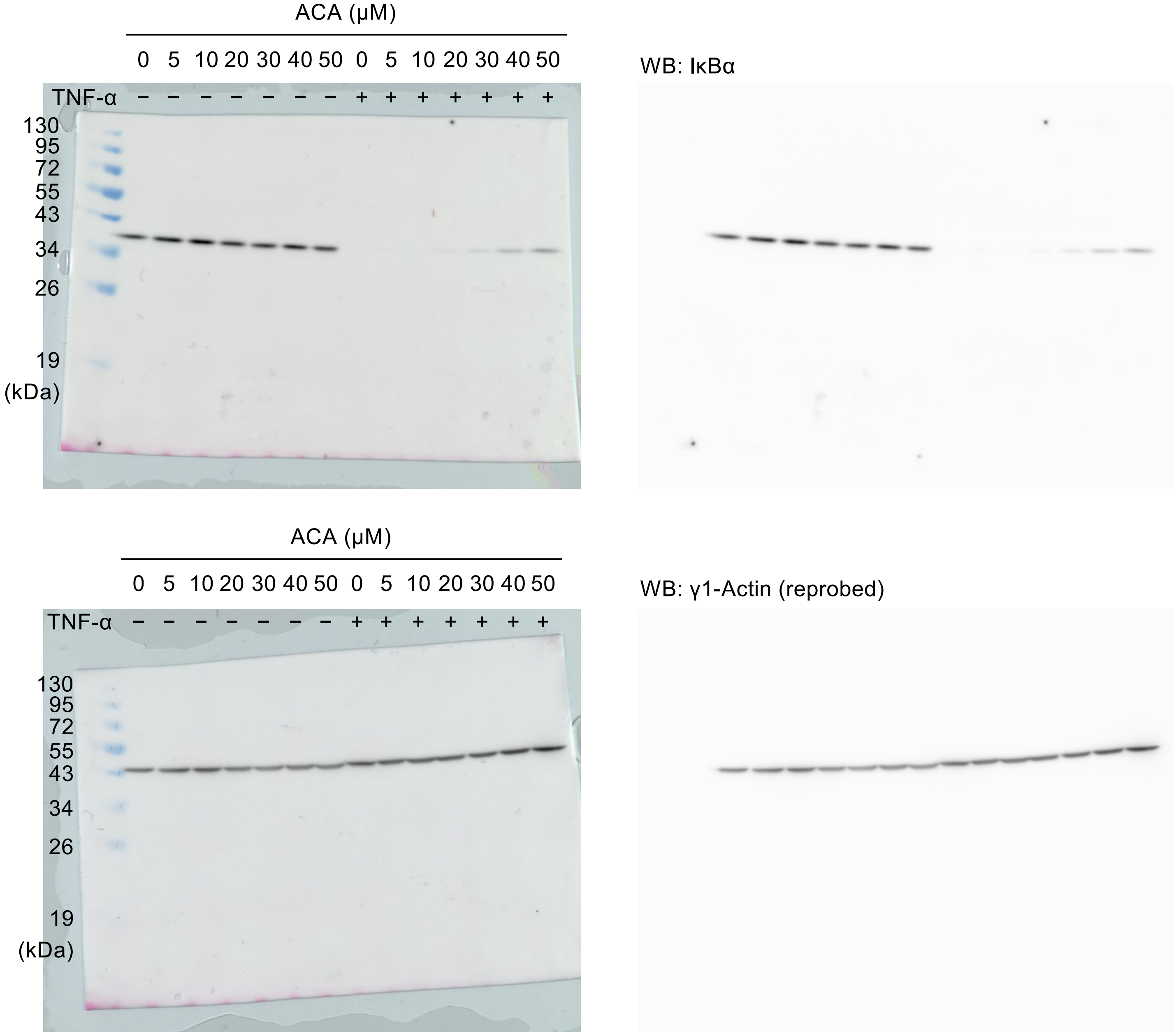

Figure S5: Original blots in Figure 6A

ACA (μM)

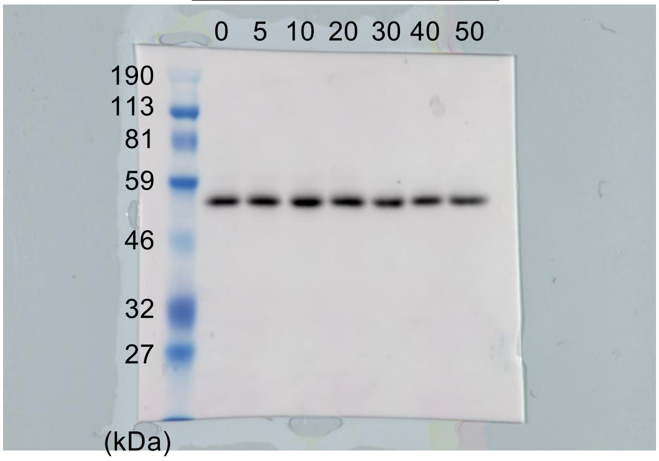

WB: TNF receptor 1

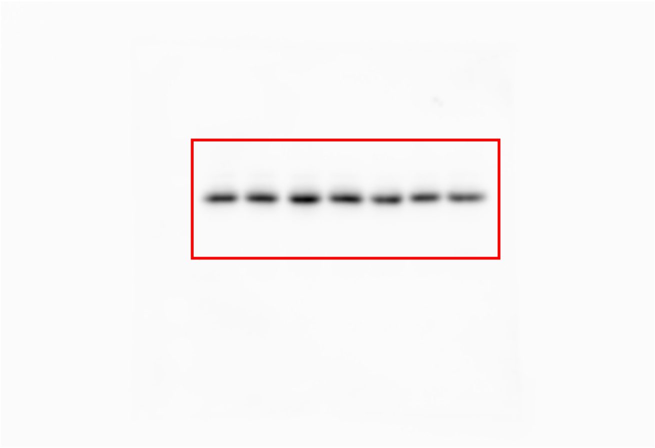

ACA (μM)

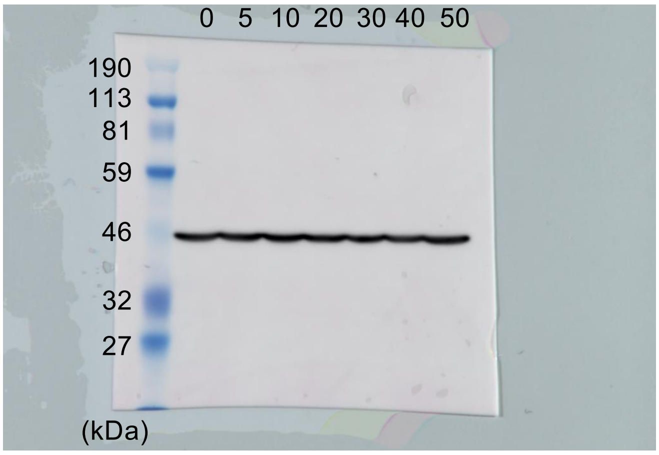

WB: β-Actin (reprobed)

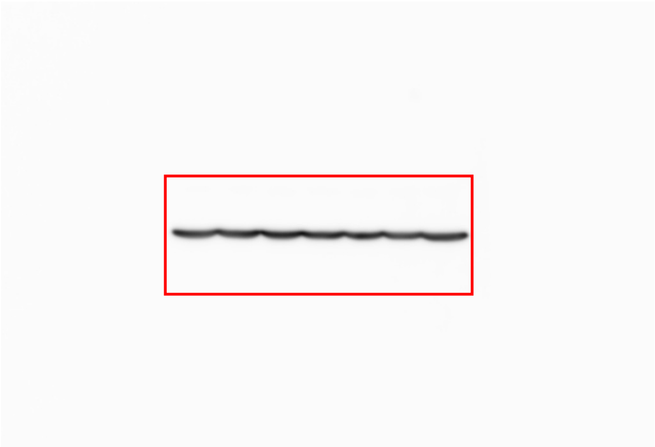

Figure S6: Original blots (1) in Figure 6B

ACA (μM)

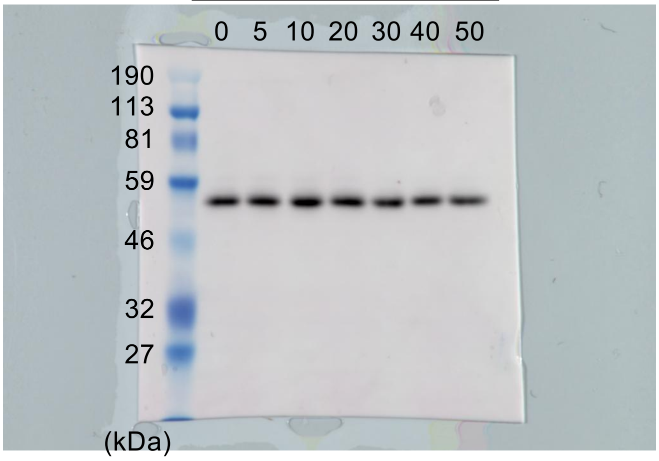

WB: TNF receptor 1

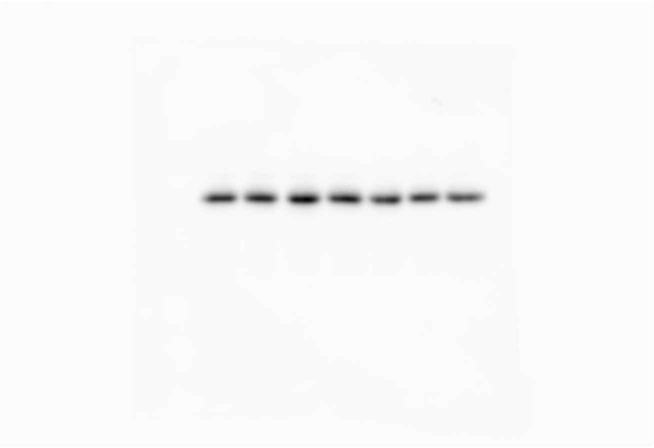

ACA (μM)

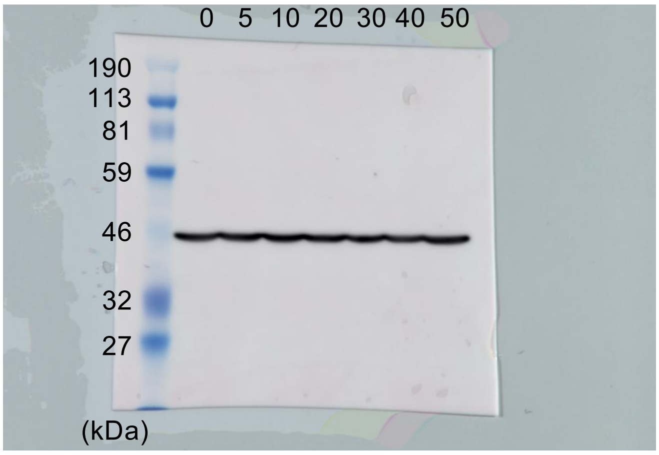

WB: β-Actin (reprobed)

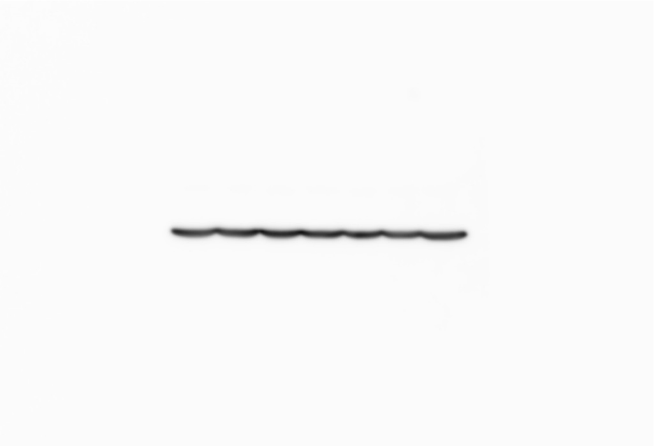

Figure S7: Original blots (2) in Figure 6B

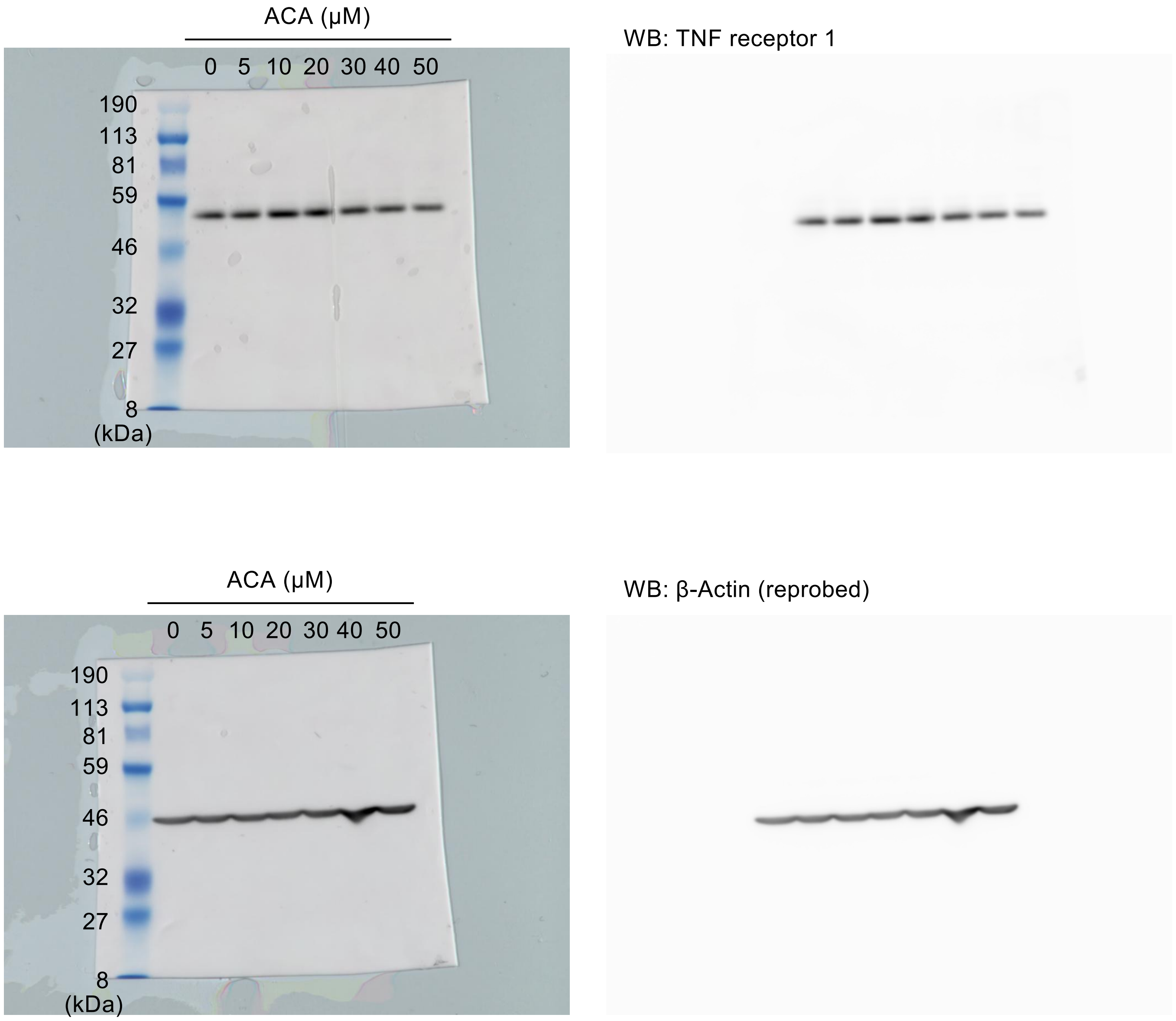

Figure S8: Original blots (3) in Figure 6B

ACA (μM)

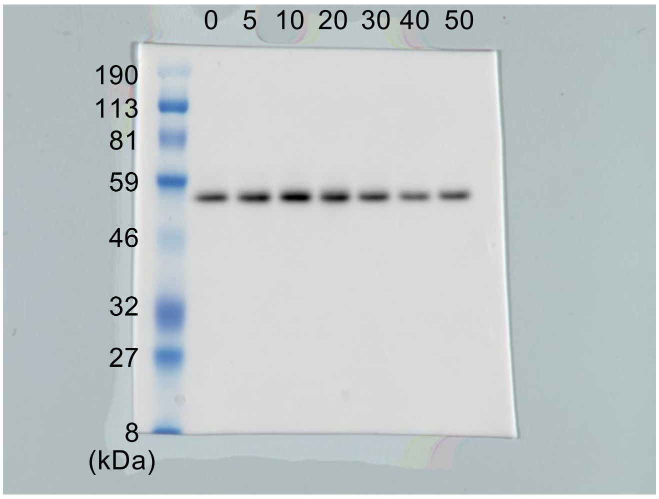

WB: TNF receptor 1

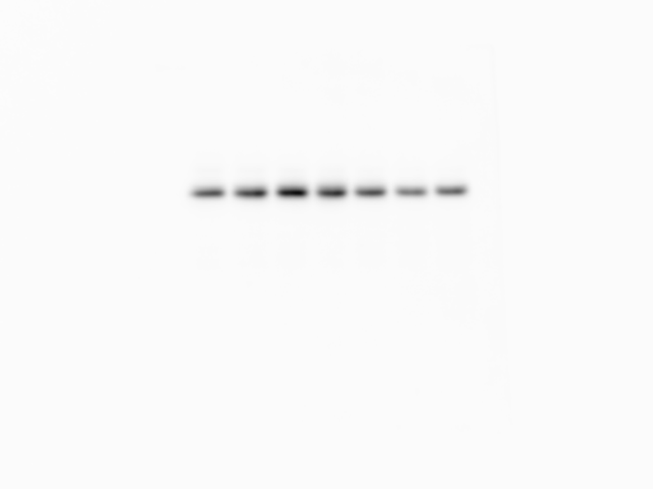

ACA (μM)

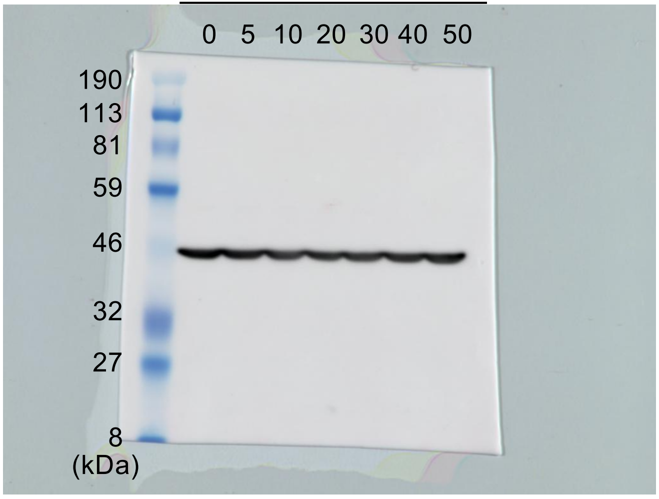

WB: β-Actin (reprobed)

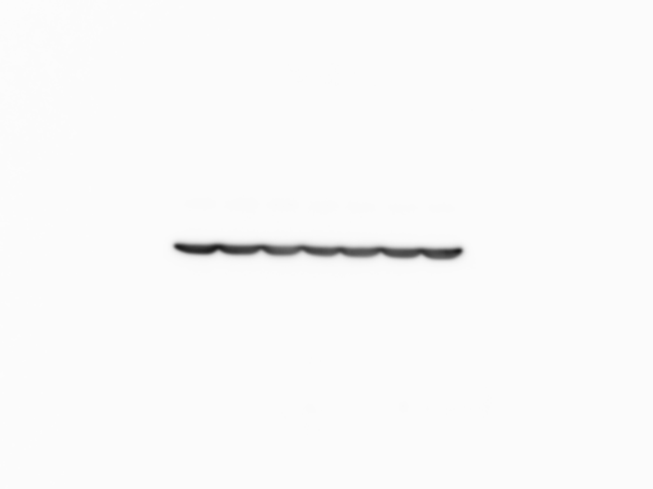

Figure S9: Original blots in Figure 6C

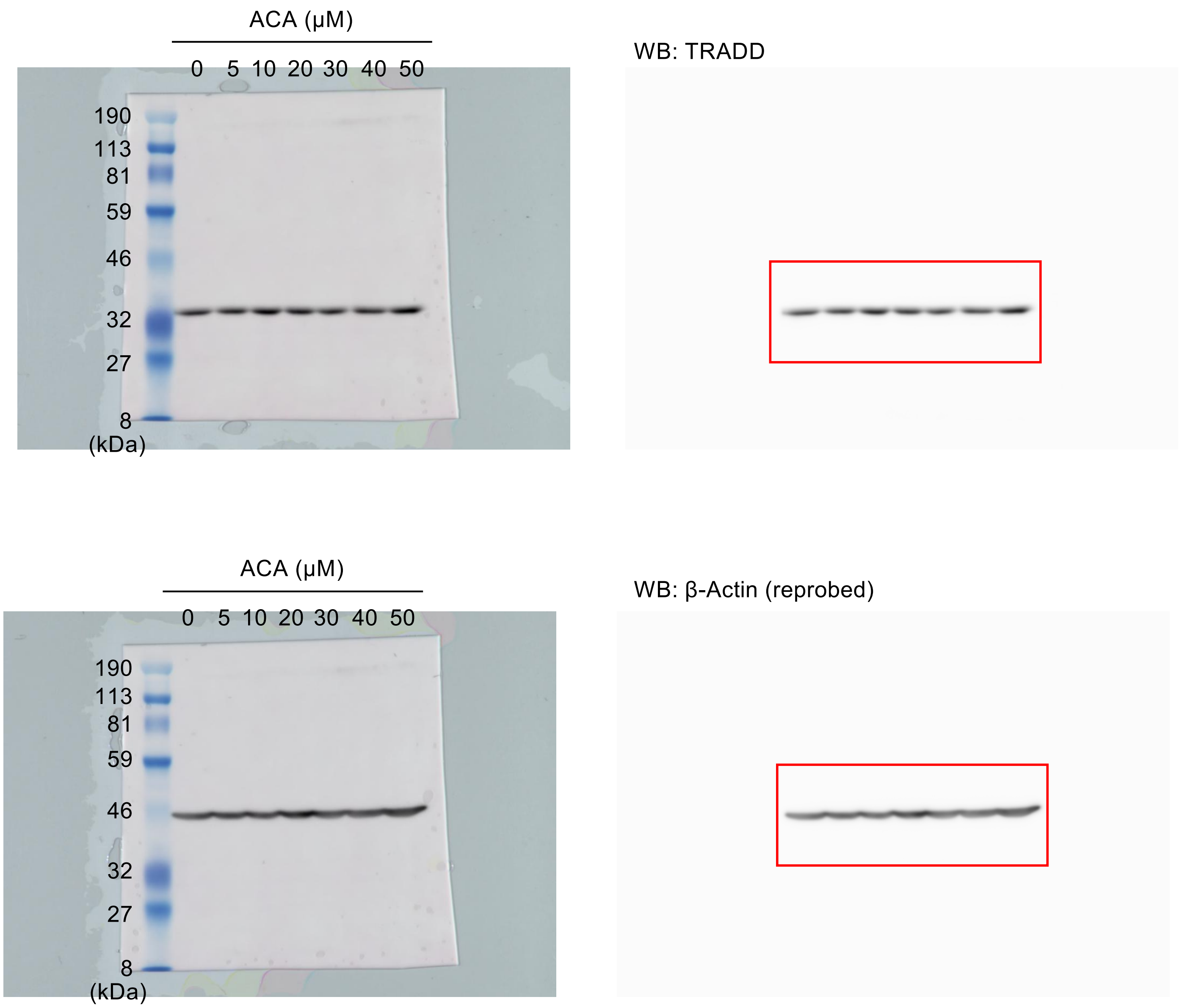

Figure S10: Original blots (1) in Figure 6D

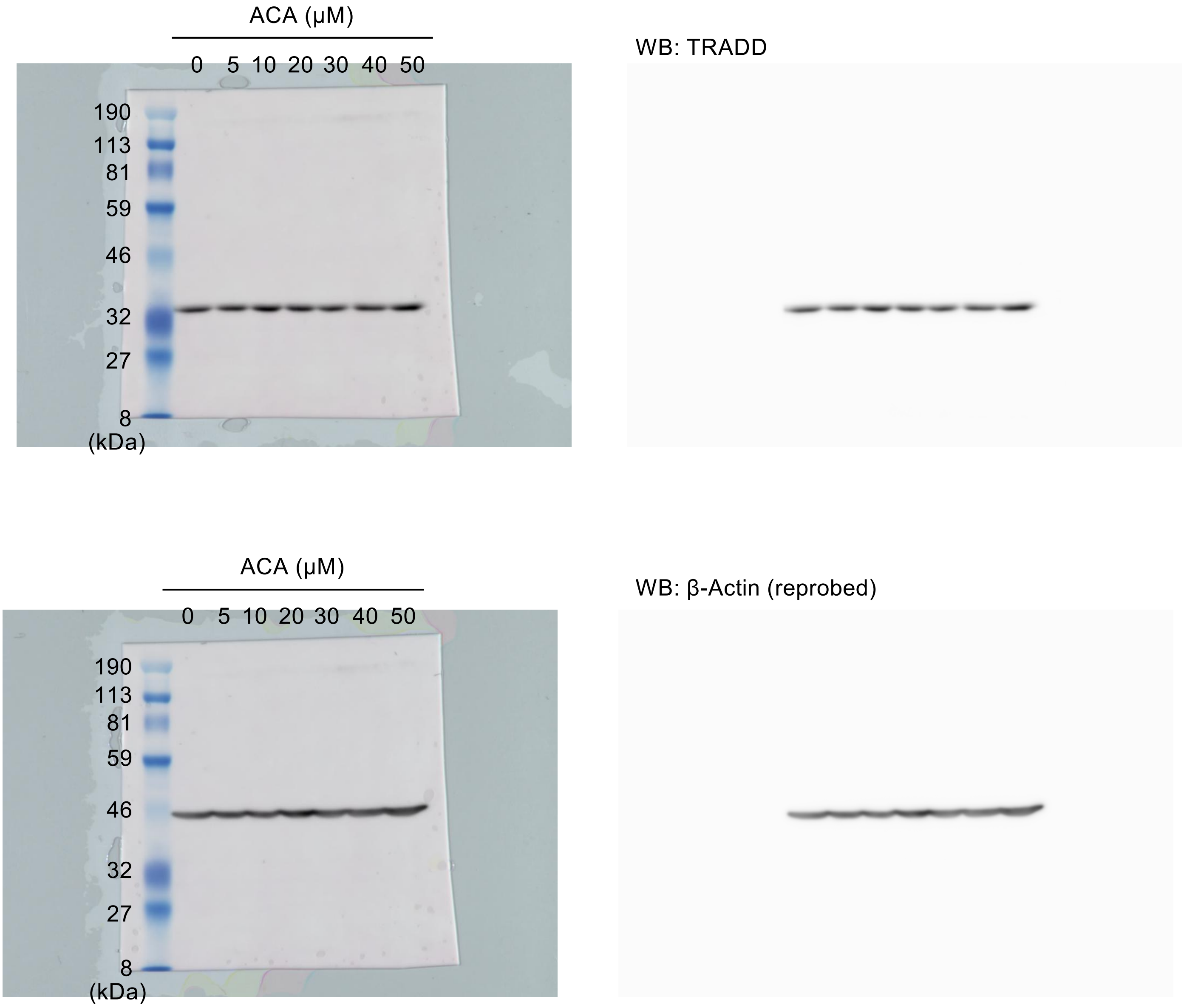

Figure S11: Original blots (2) in Figure 6D

ACA (μM)

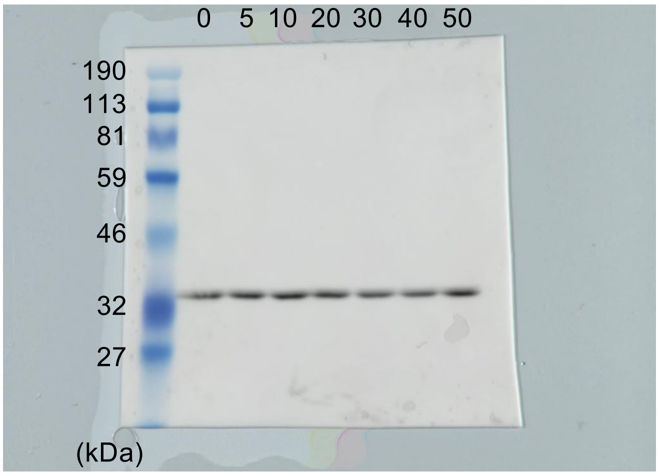

WB: TRADD

ACA (μM)

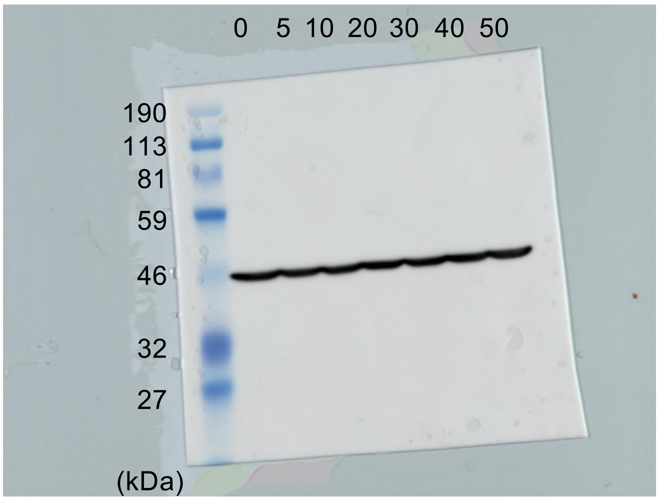

WB: β-Actin (reprobed)

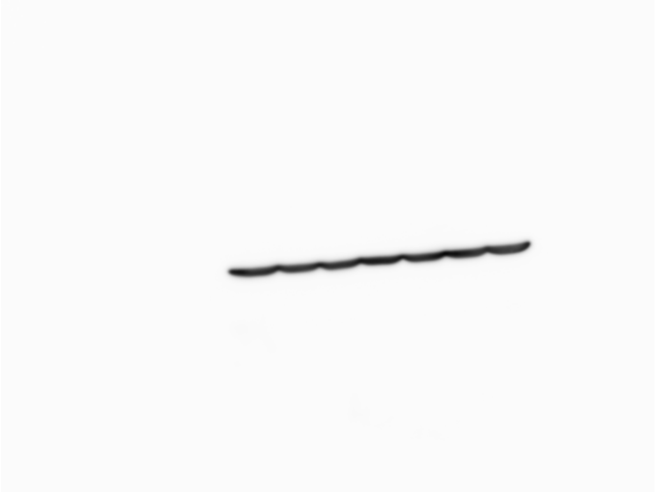

Figure S12: Original blots (3) in Figure 6D

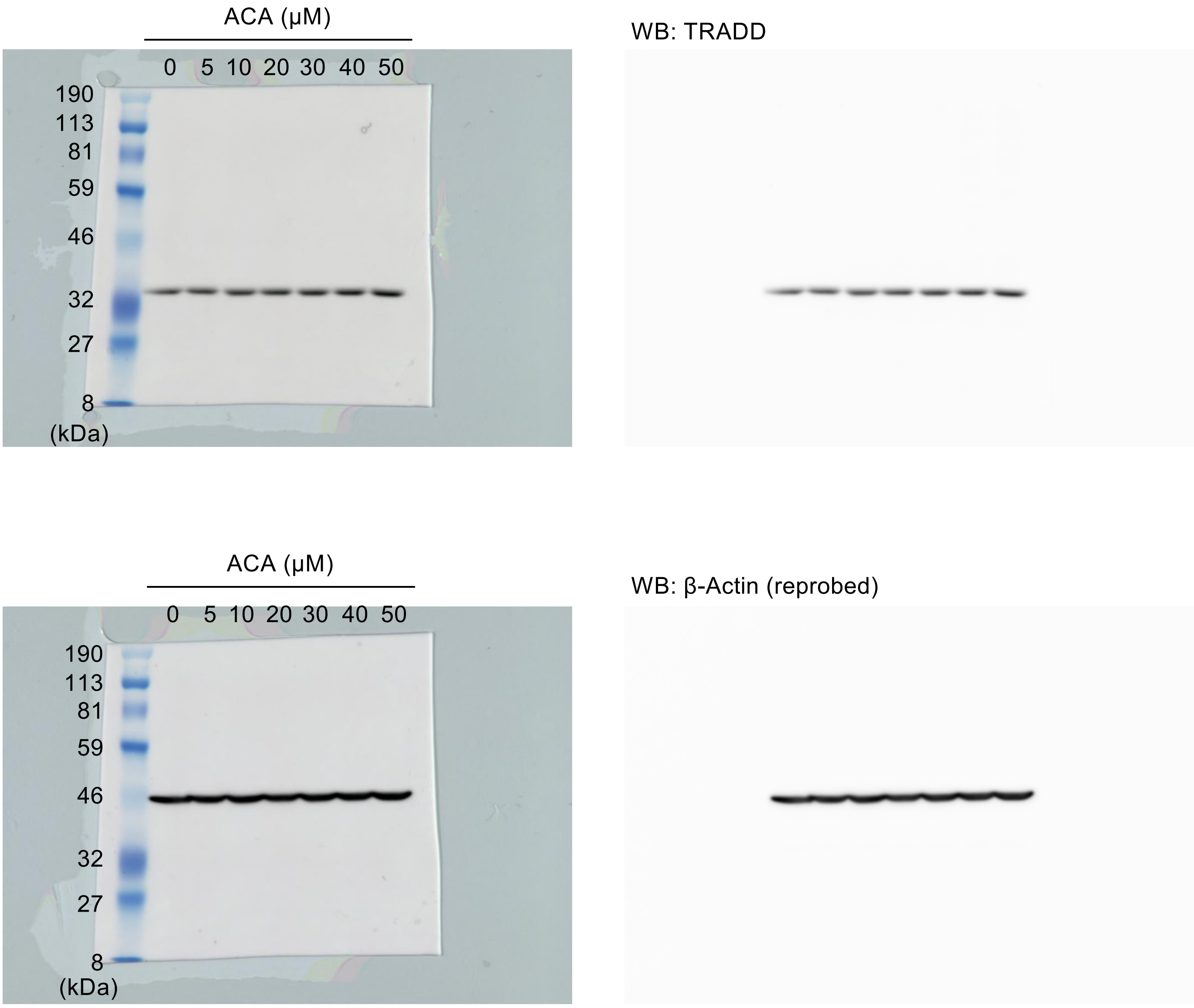

Figure S13: Original blots in Figure 6E

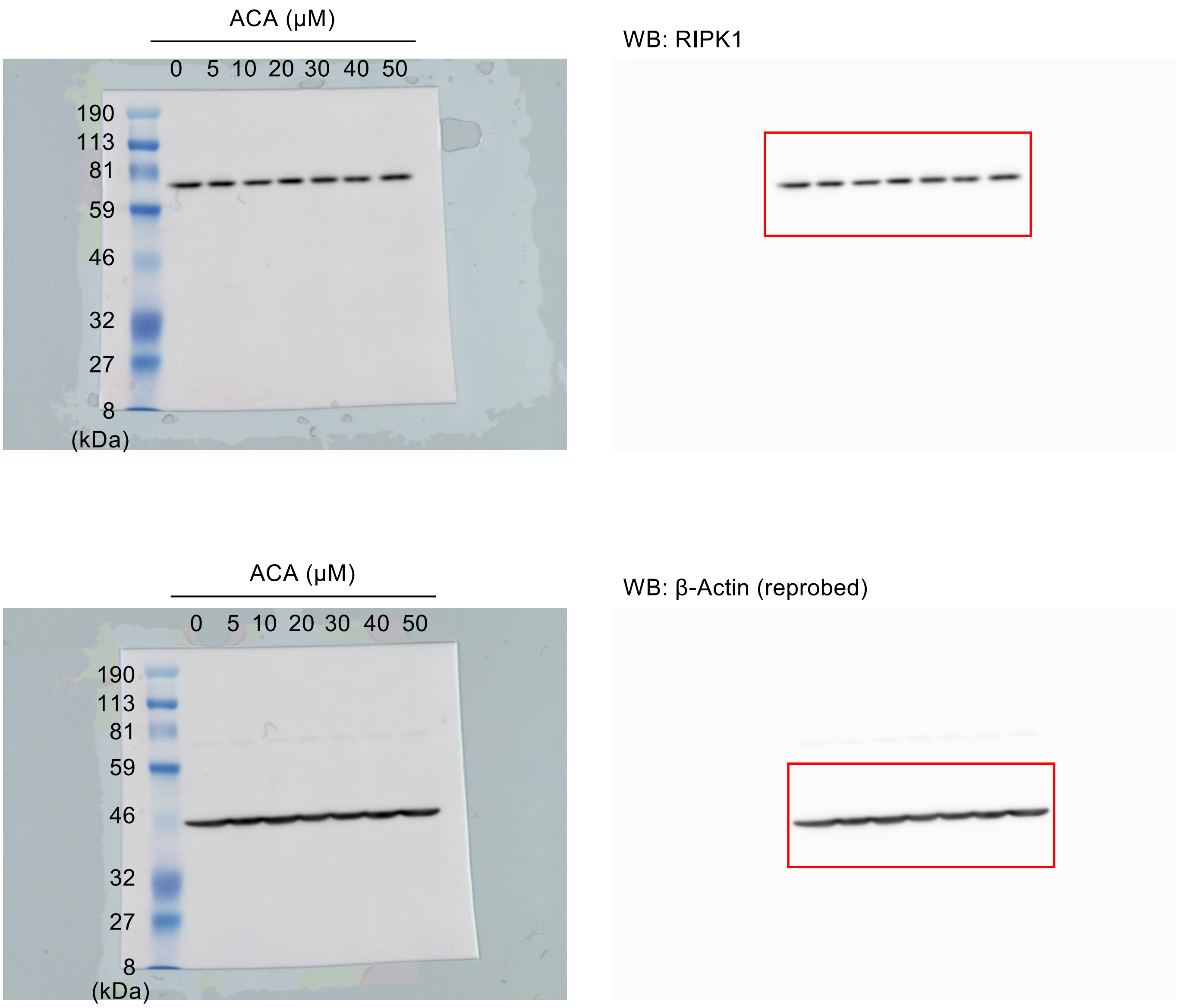

Figure S14: Original blots (1) in Figure 6F

ACA (μM)

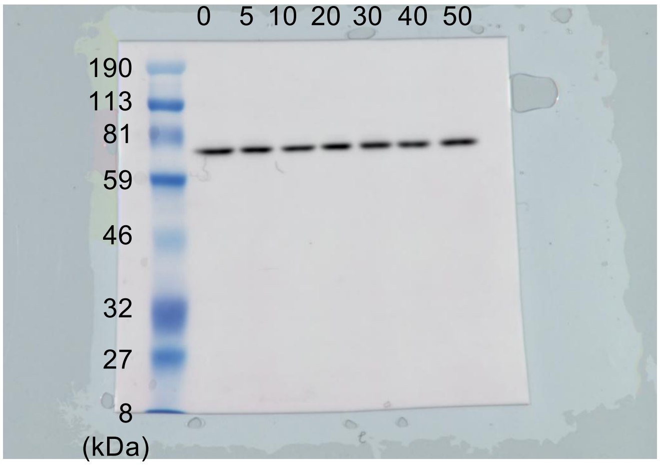

WB: RIPK1

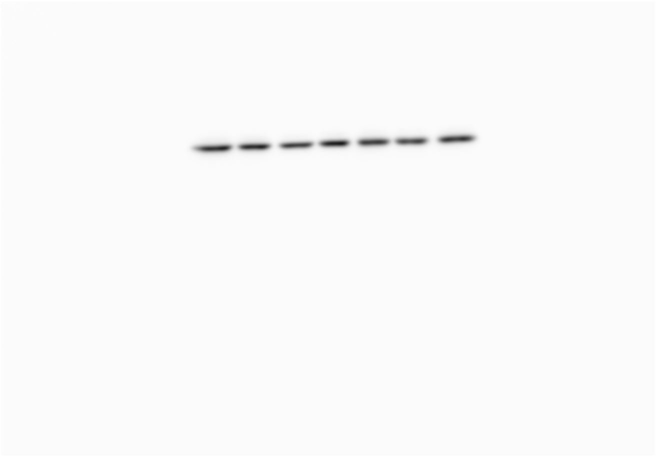

ACA (μM)

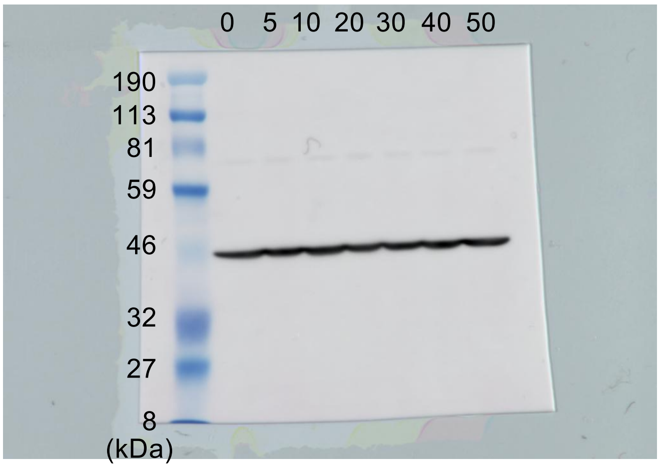

WB: β-Actin (reprobed)

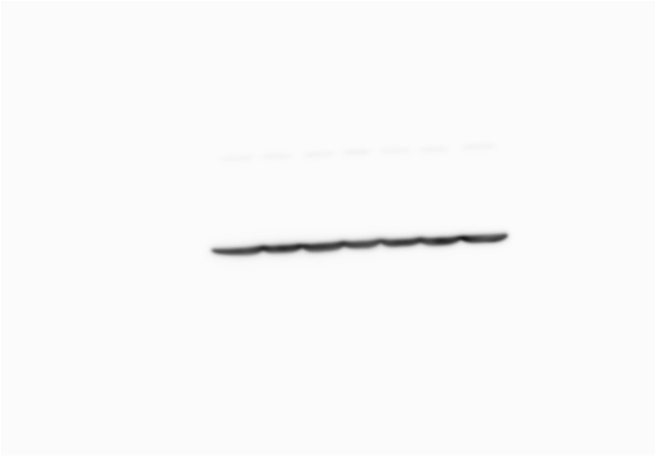

Figure S15: Original blots (2) in Figure 6F

ACA (µM)

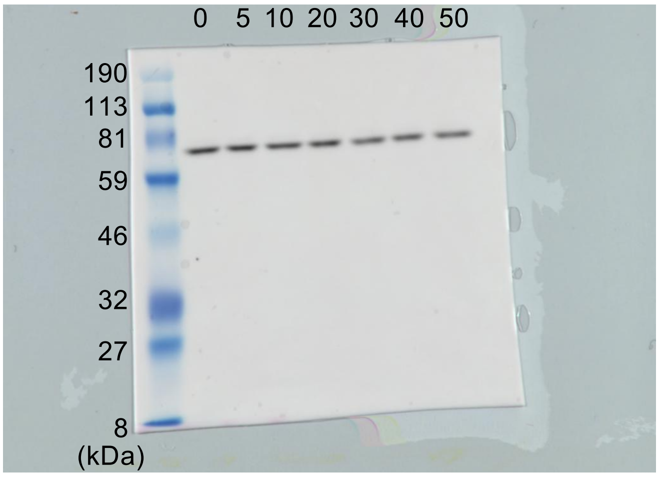

WB: RIPK1

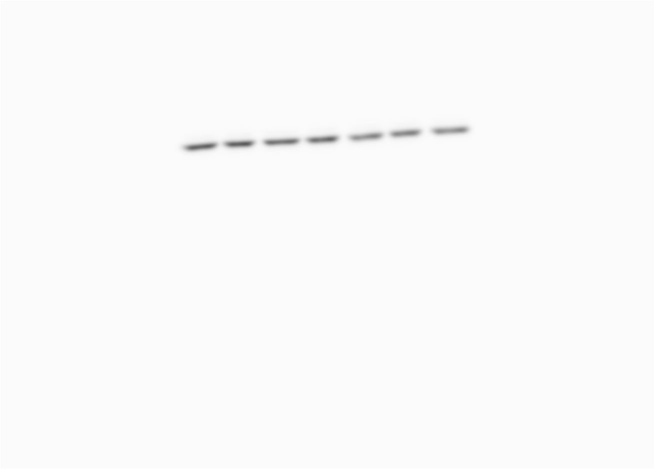

ACA (µM)

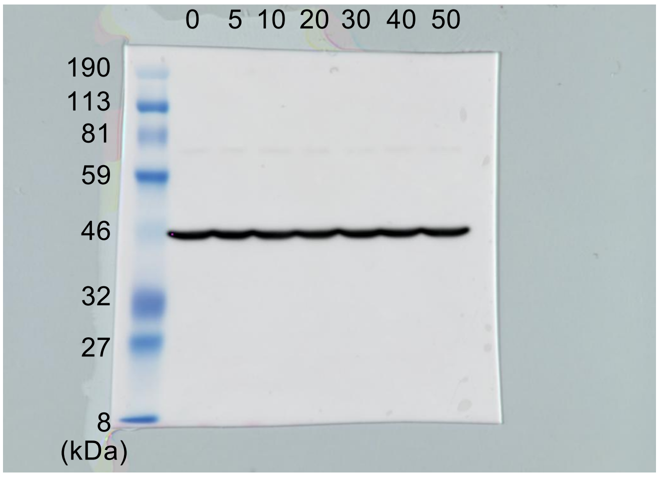

WB: β-Actin (reprobed)

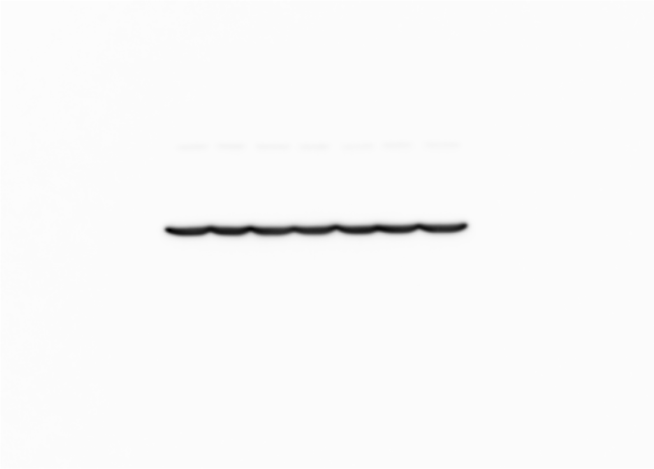

Figure S16: Original blots (3) in Figure 6F

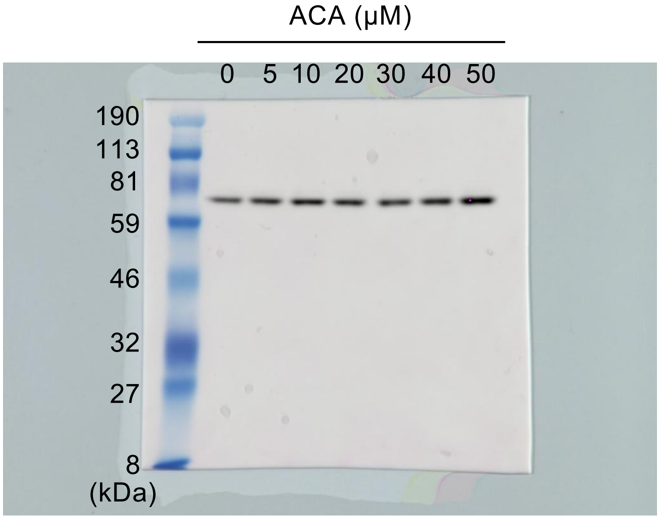

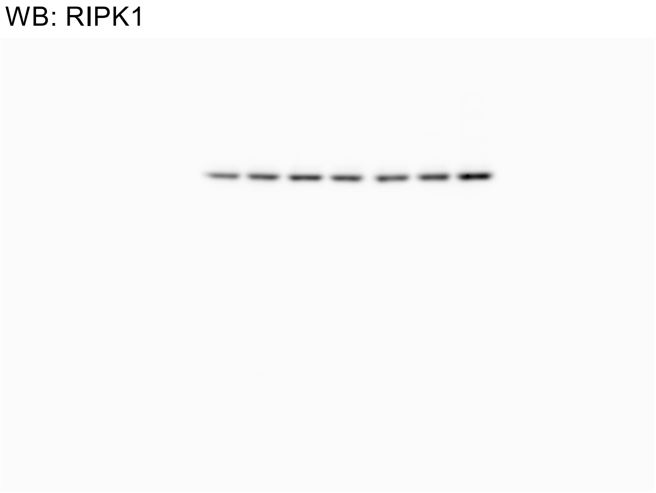

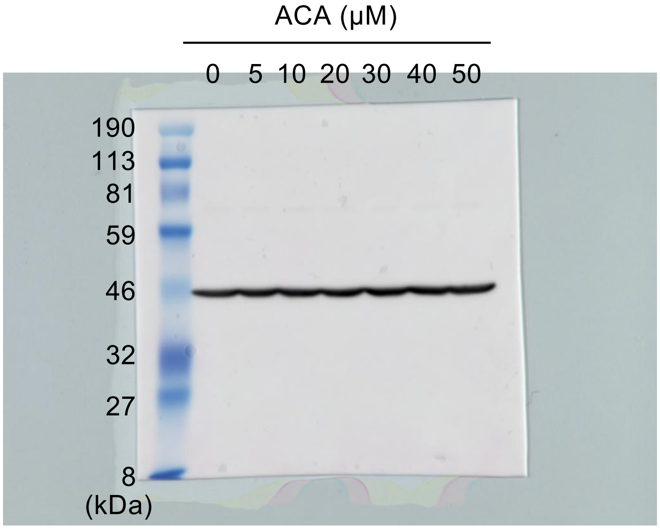

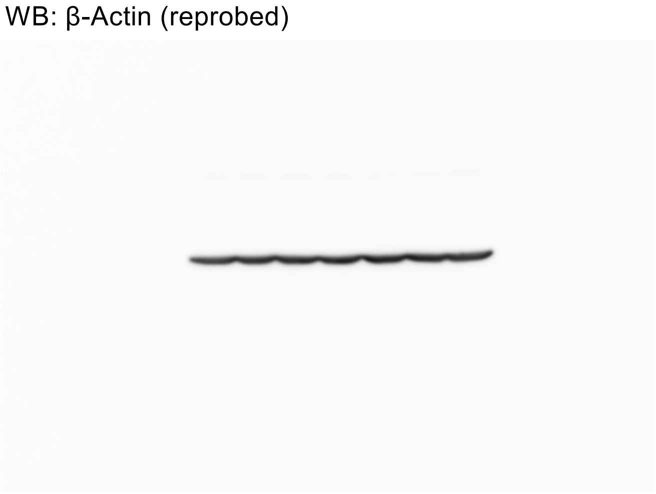

Figure S17: Original blots in Figure 6G

ACA (µM)

0 5 10 20 30 40 50

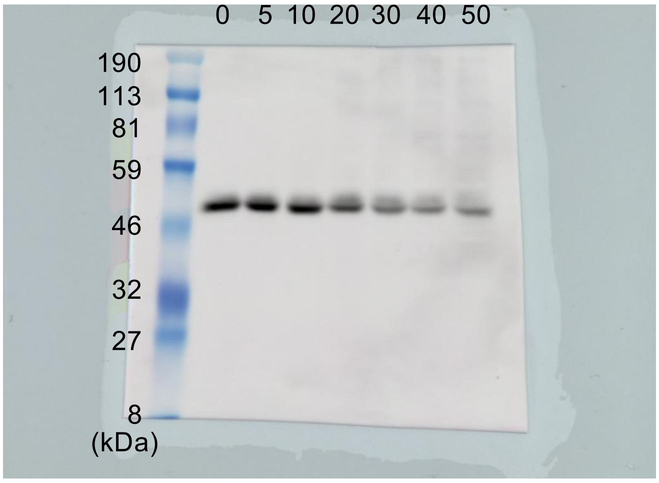

WB: TRAF2

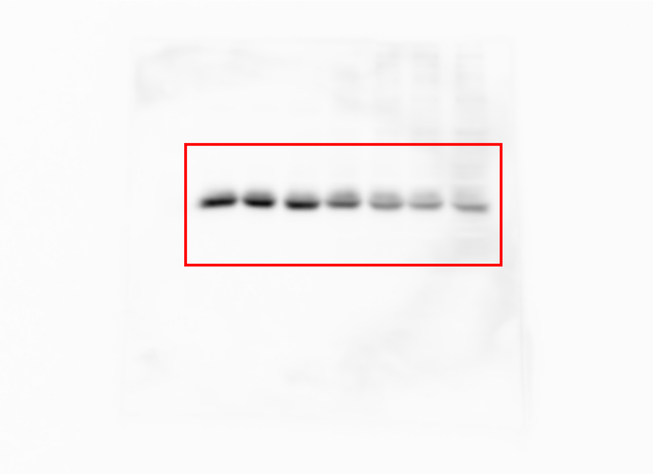

ACA (µM)

0 5 10 20 30 40 50

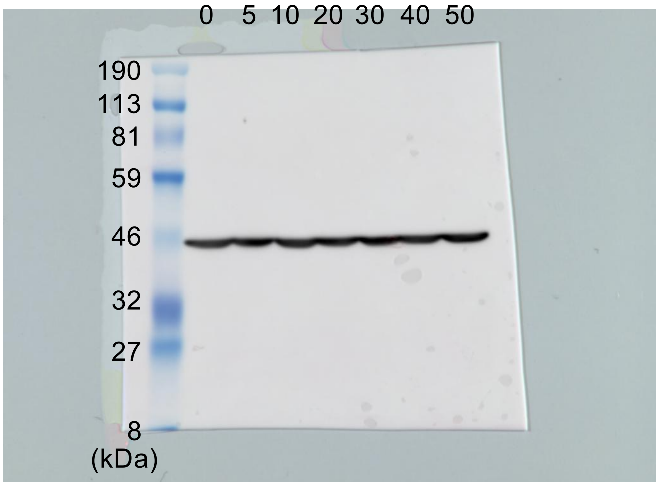

WB: β-Actin (reprobed)

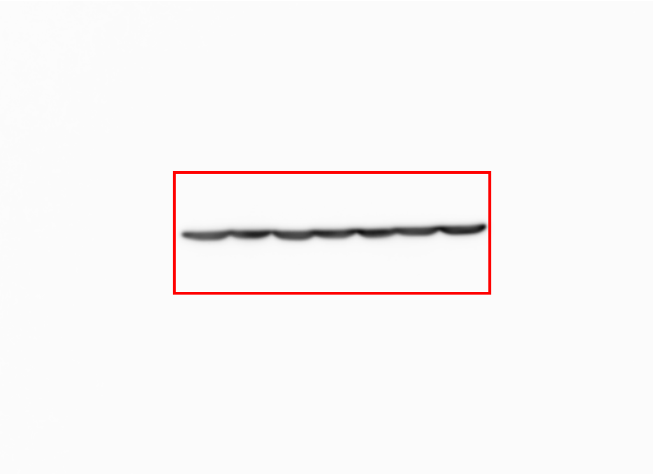

Figure S18: Original blots (1) in Figure 6H

ACA (μM)

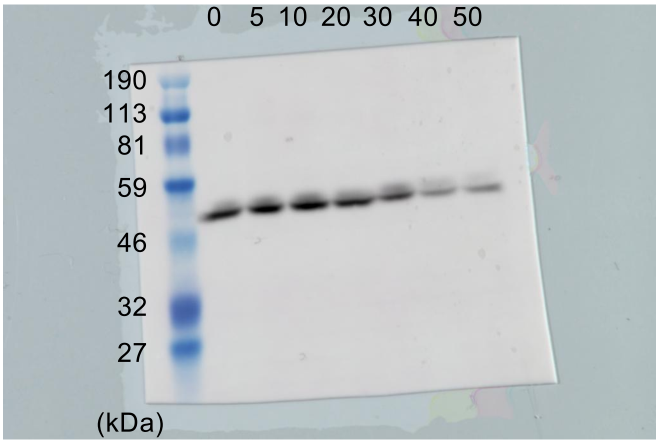

WB: TRAF2

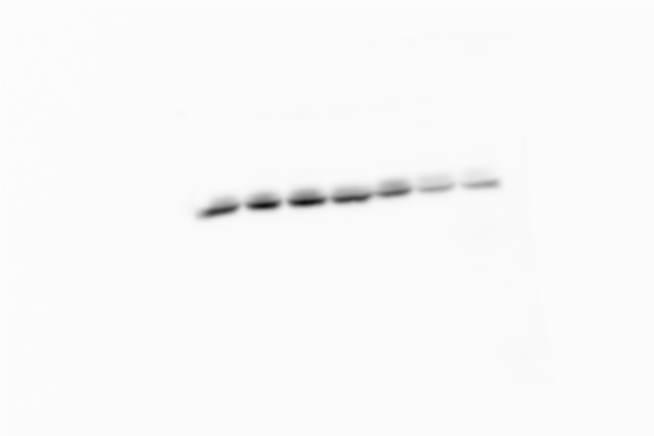

ACA (μM)

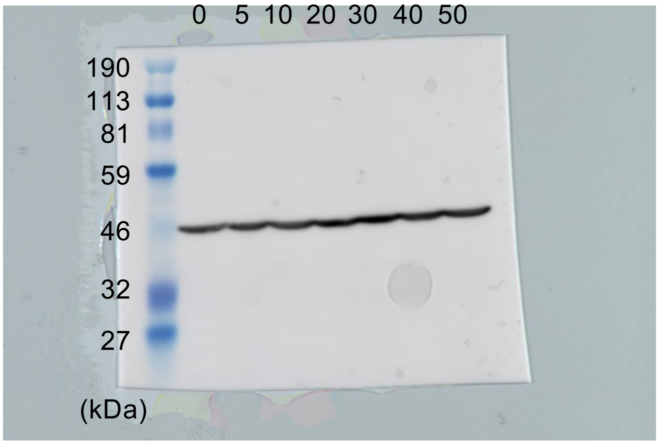

WB: β-Actin (reprobed)

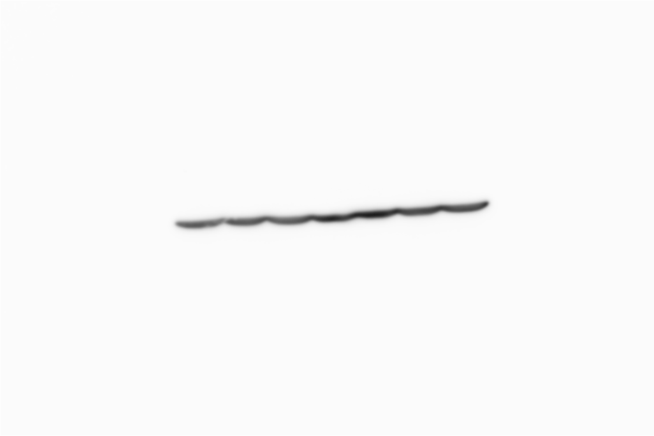

Figure S19: Original blots (2) in Figure 6H

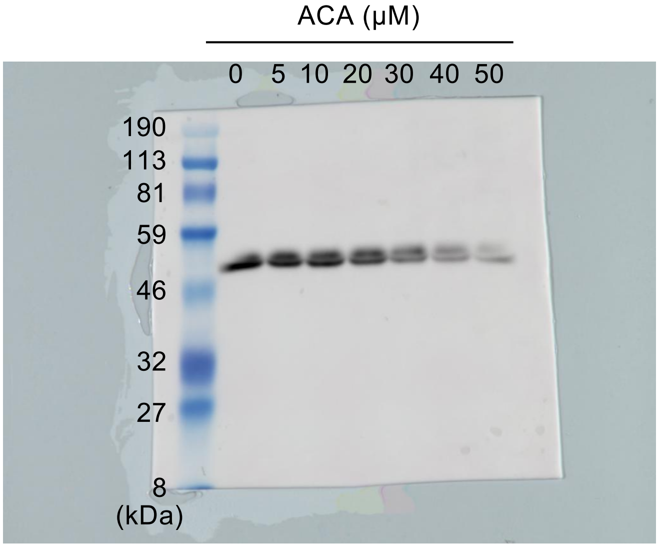

WB: TRAF2

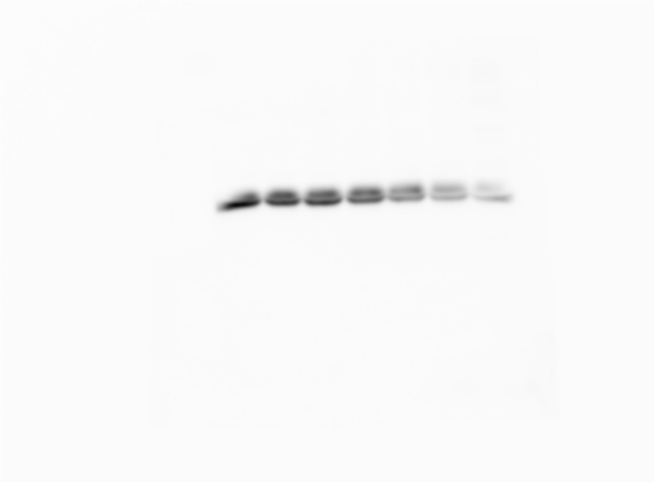

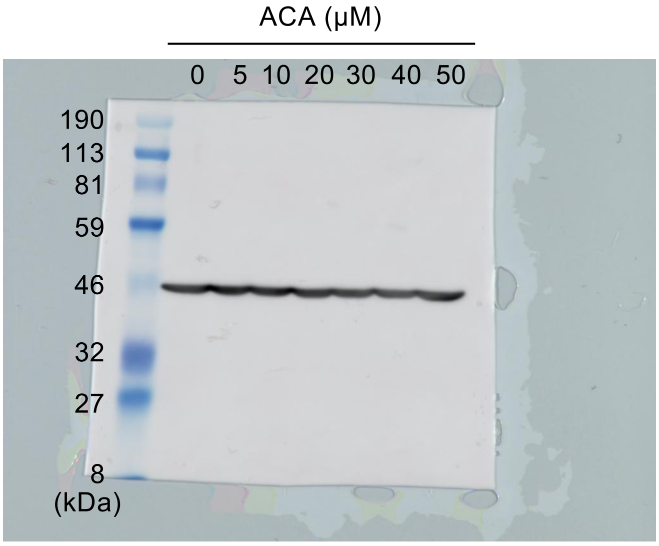

WB: β-Actin (reprobed)

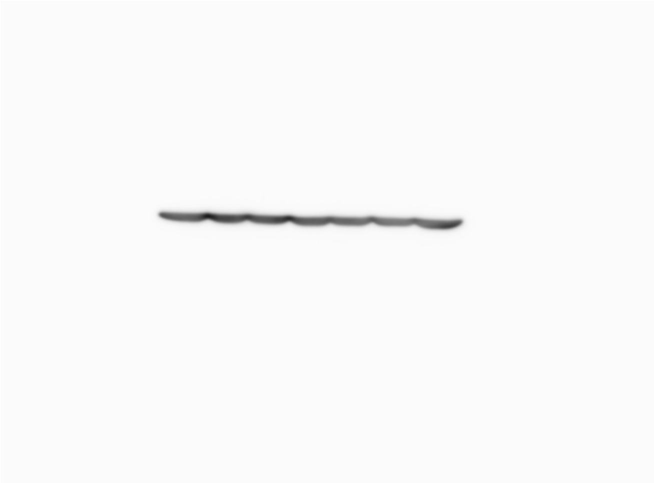

Figure S20: Original blots (3) in Figure 6H

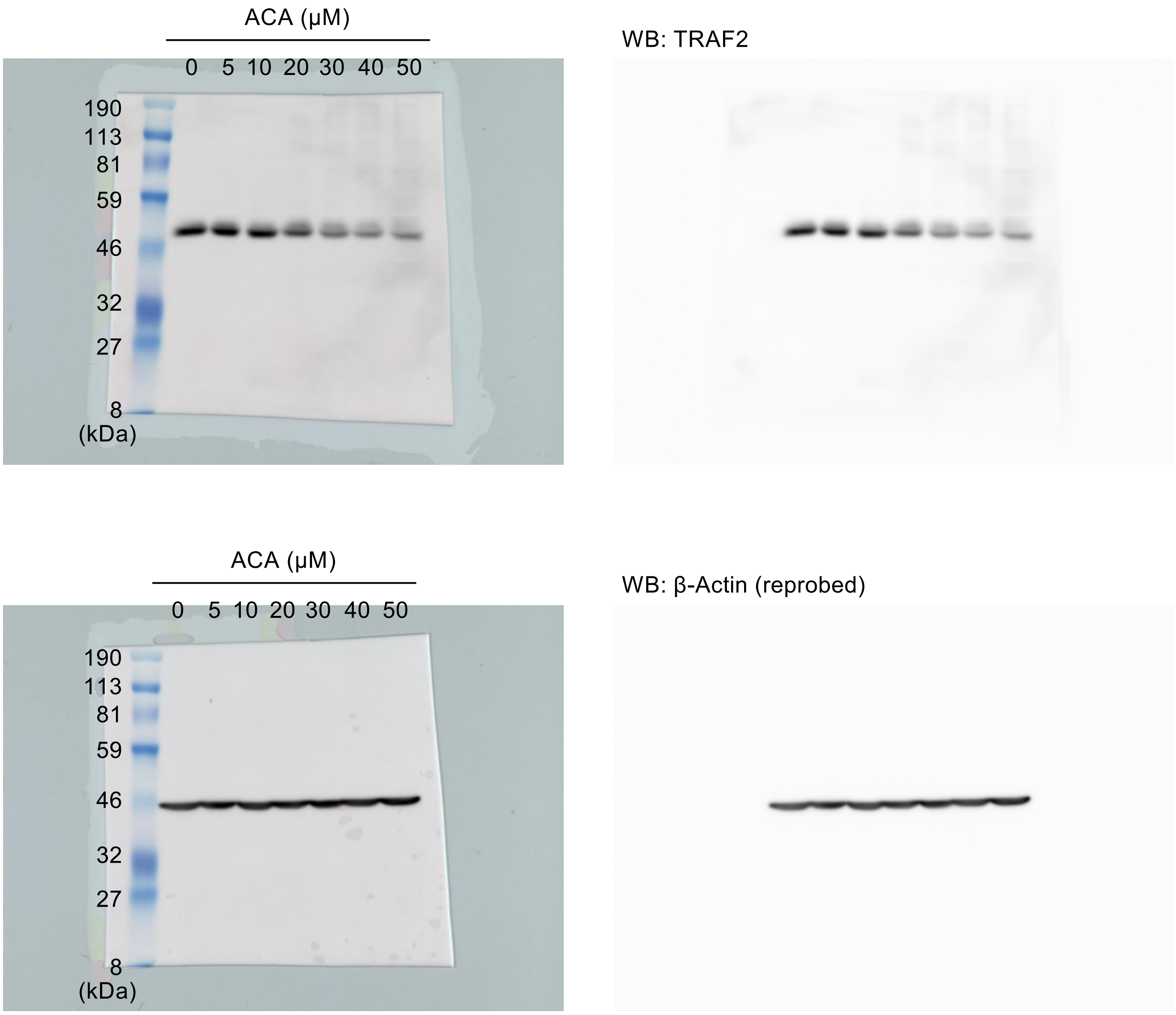

Figure S21: Original blots in Figure 7A

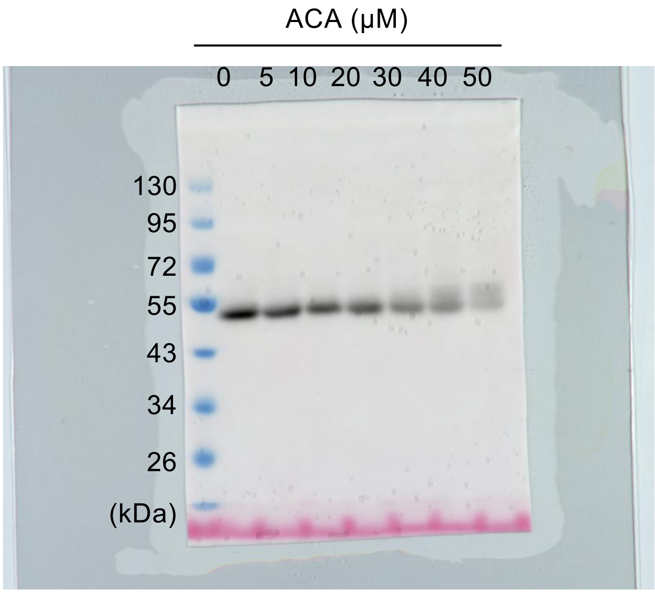

WB: TRAF2

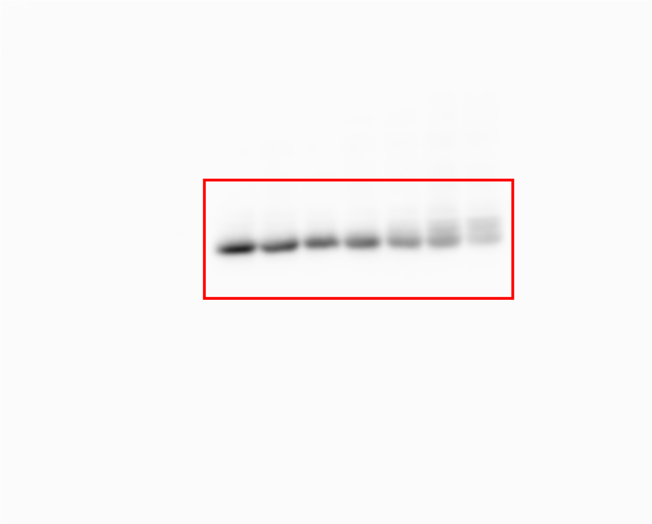

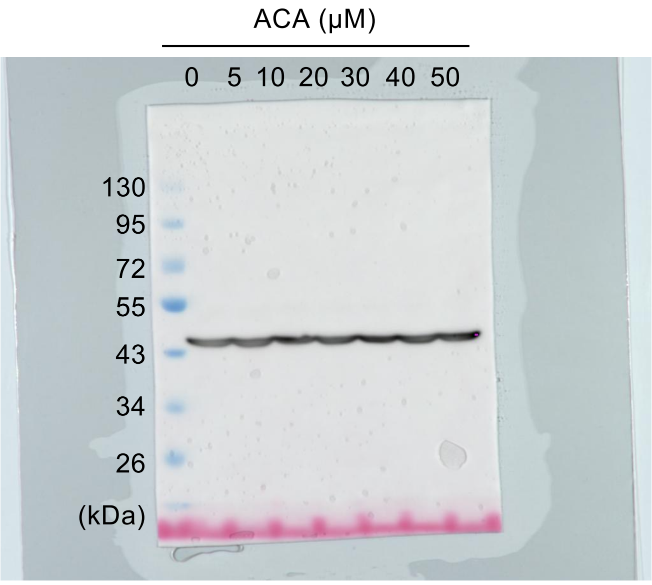

WB: β-Actin (reprobed)

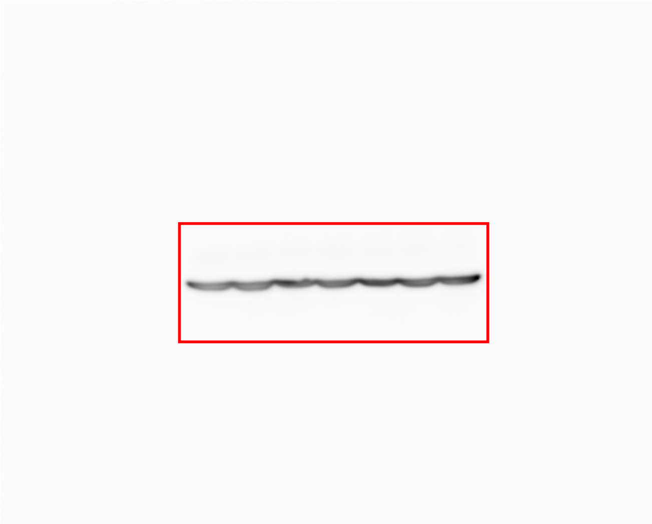

Figure S22: Original blots (1) in Figure 7B

ACA (µM)

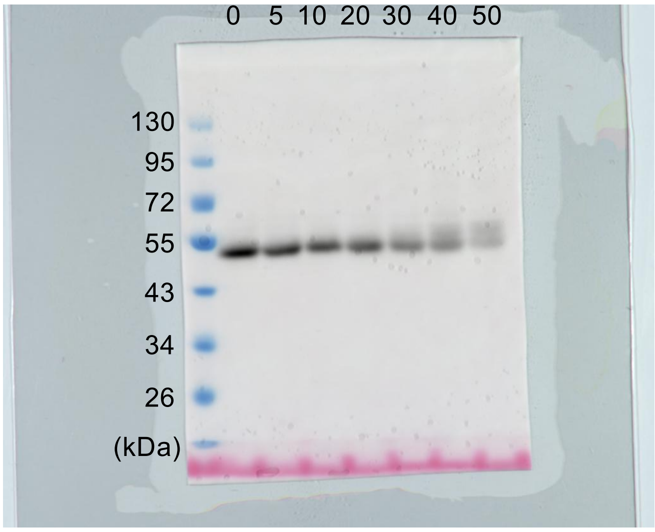

WB: TRAF2

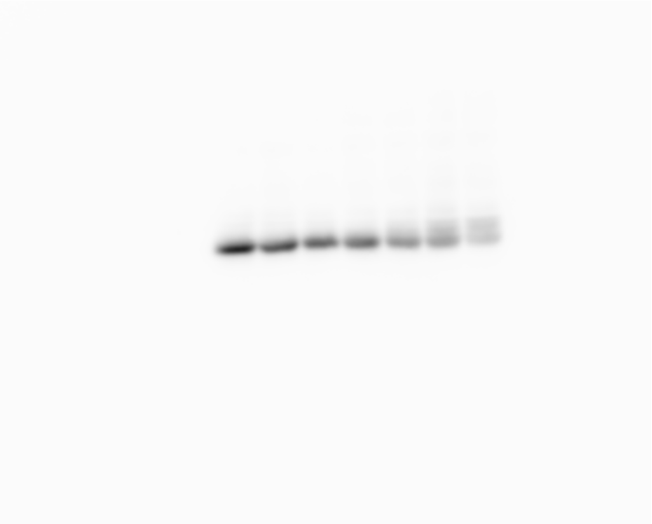

ACA (µM)

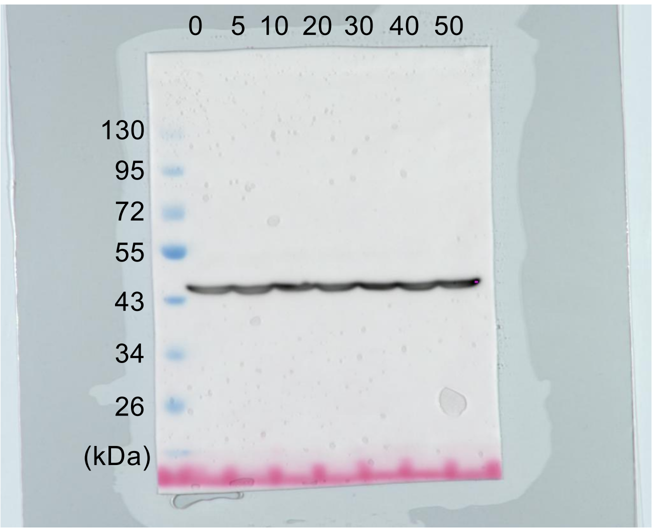

WB: β-Actin (reprobed)

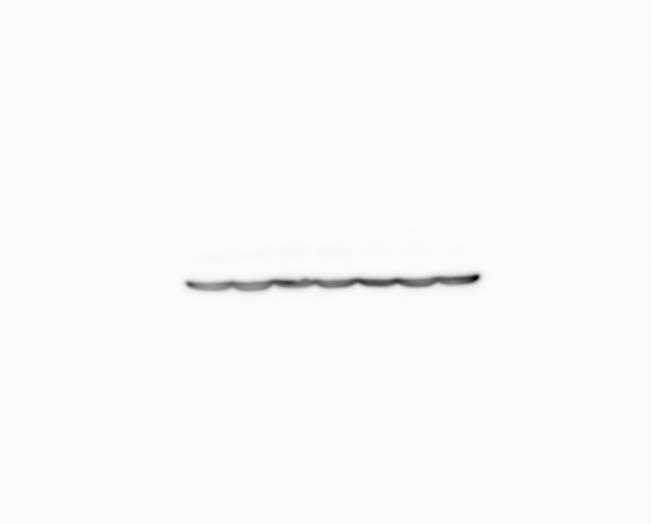

Figure S23: Original blots (2) in Figure 7B

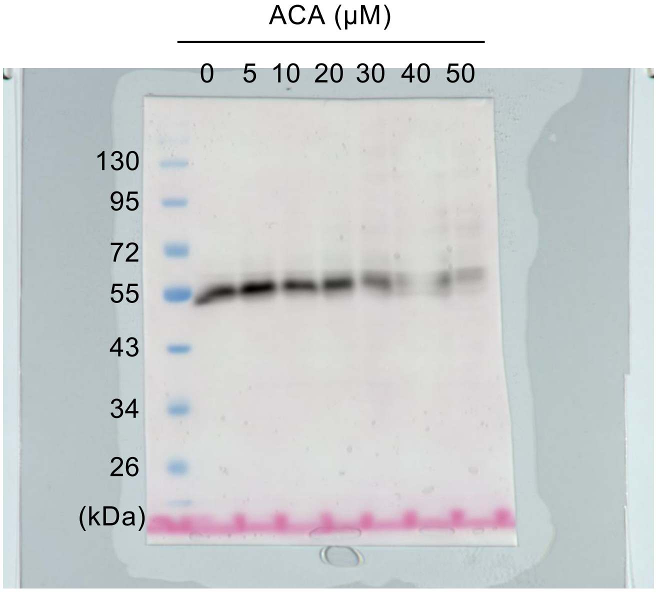

WB: TRAF2

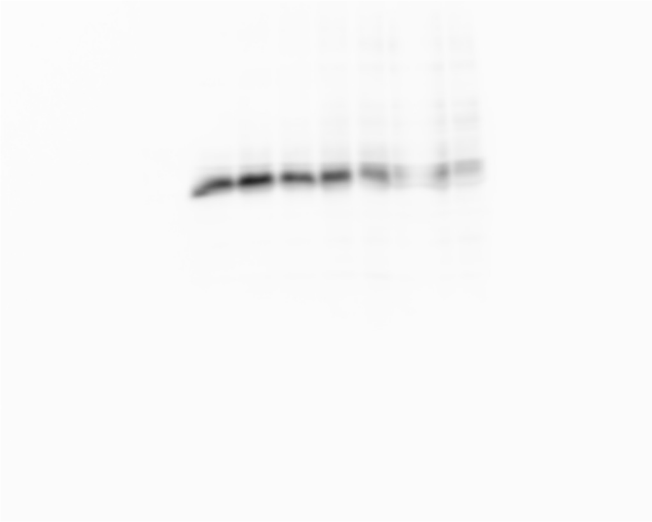

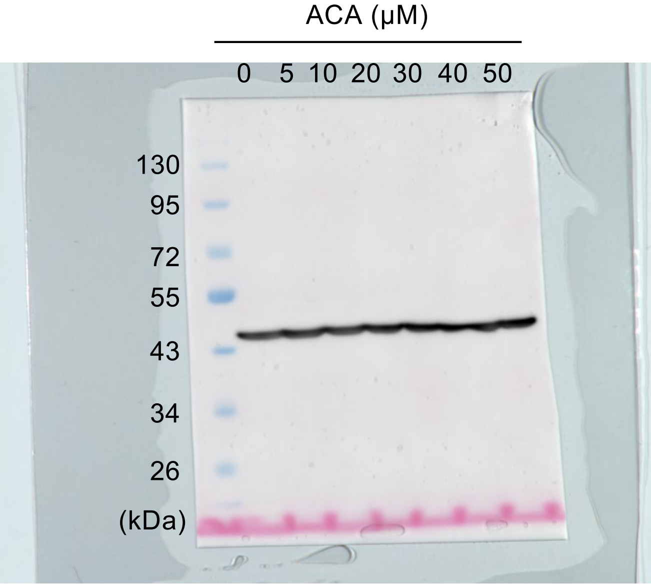

WB: β-Actin (reprobed)

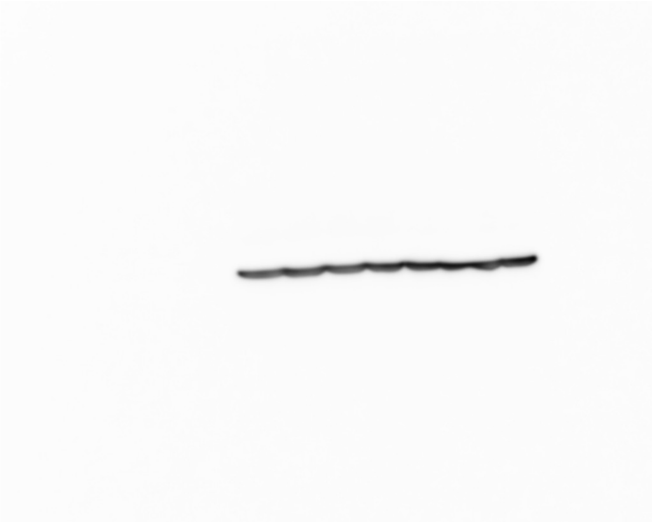

Figure S24: Original blots (3) in Figure 7B

ACA (μM)

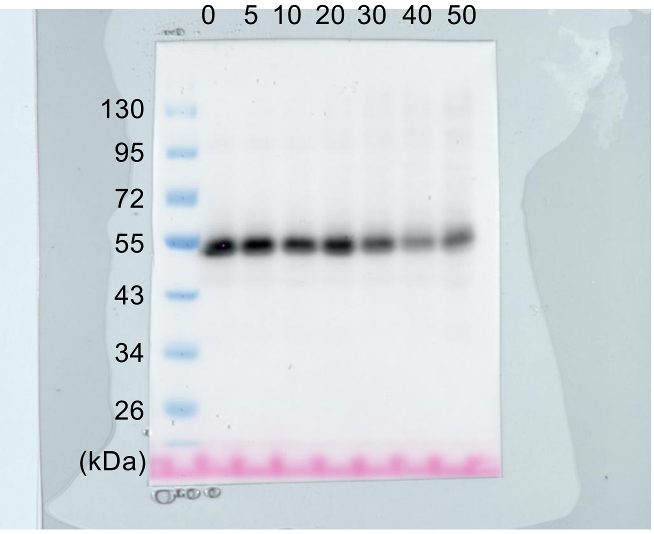

WB: TRAF2

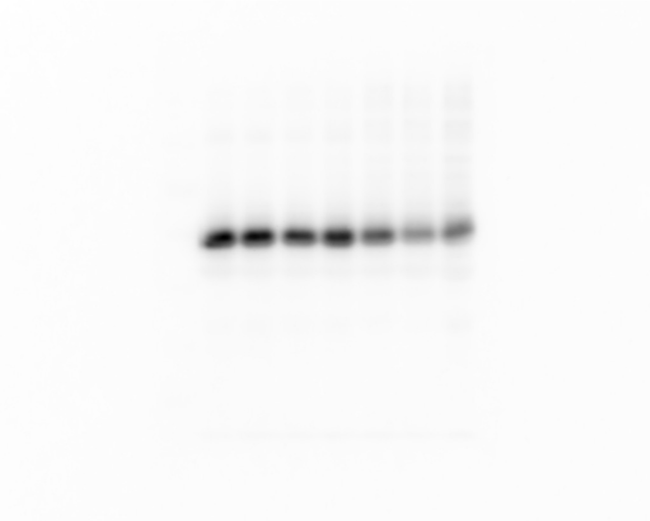

ACA (μM)

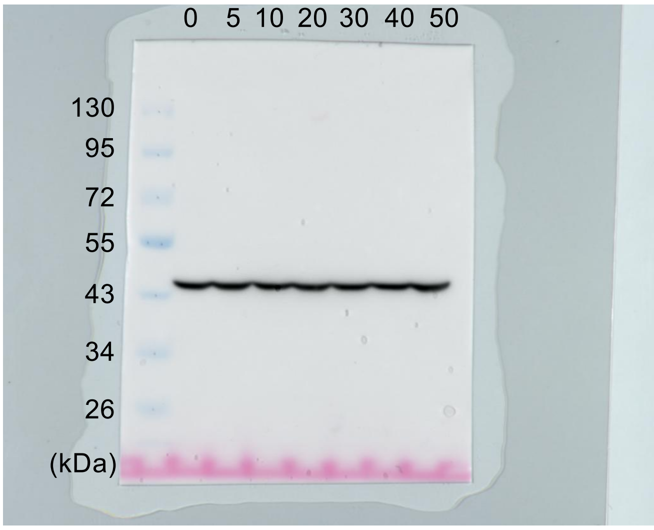

WB: β-Actin (reprobed)

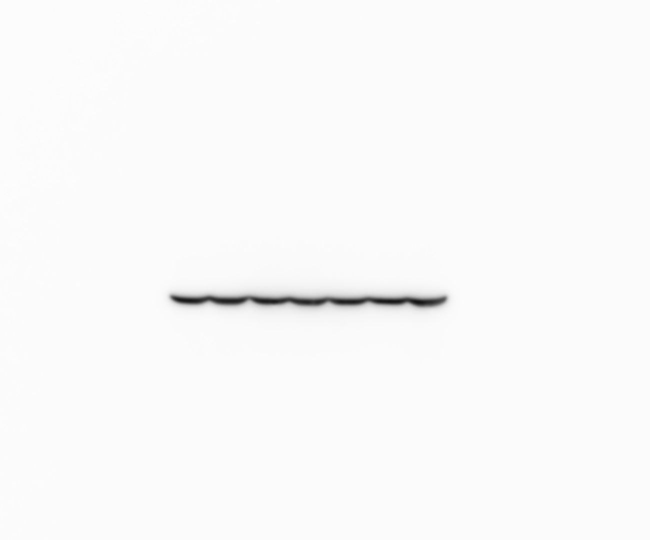

Figure S25: Original blots (4) in Figure 7B

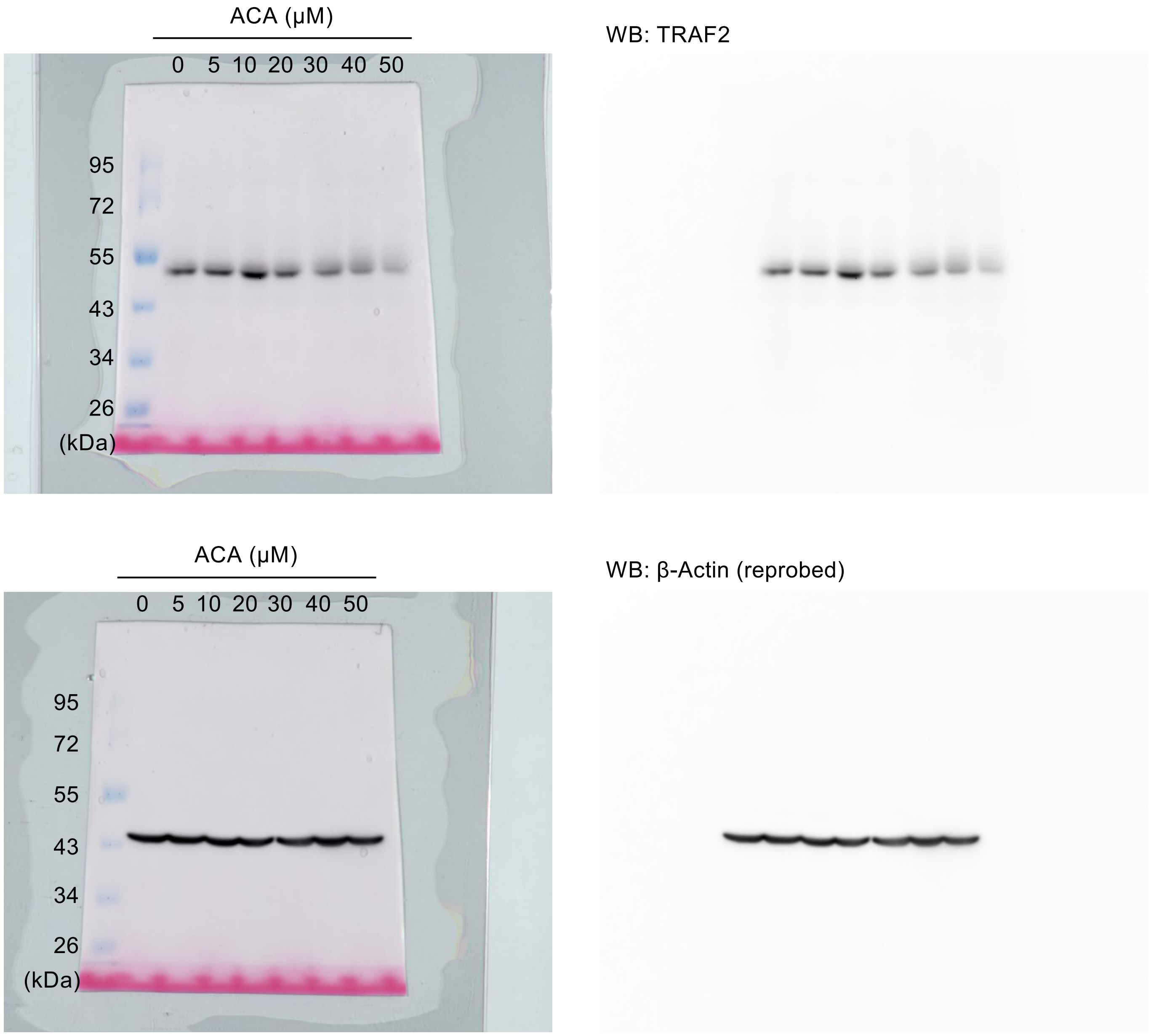

Figure S26: Original blots in Figure 7C

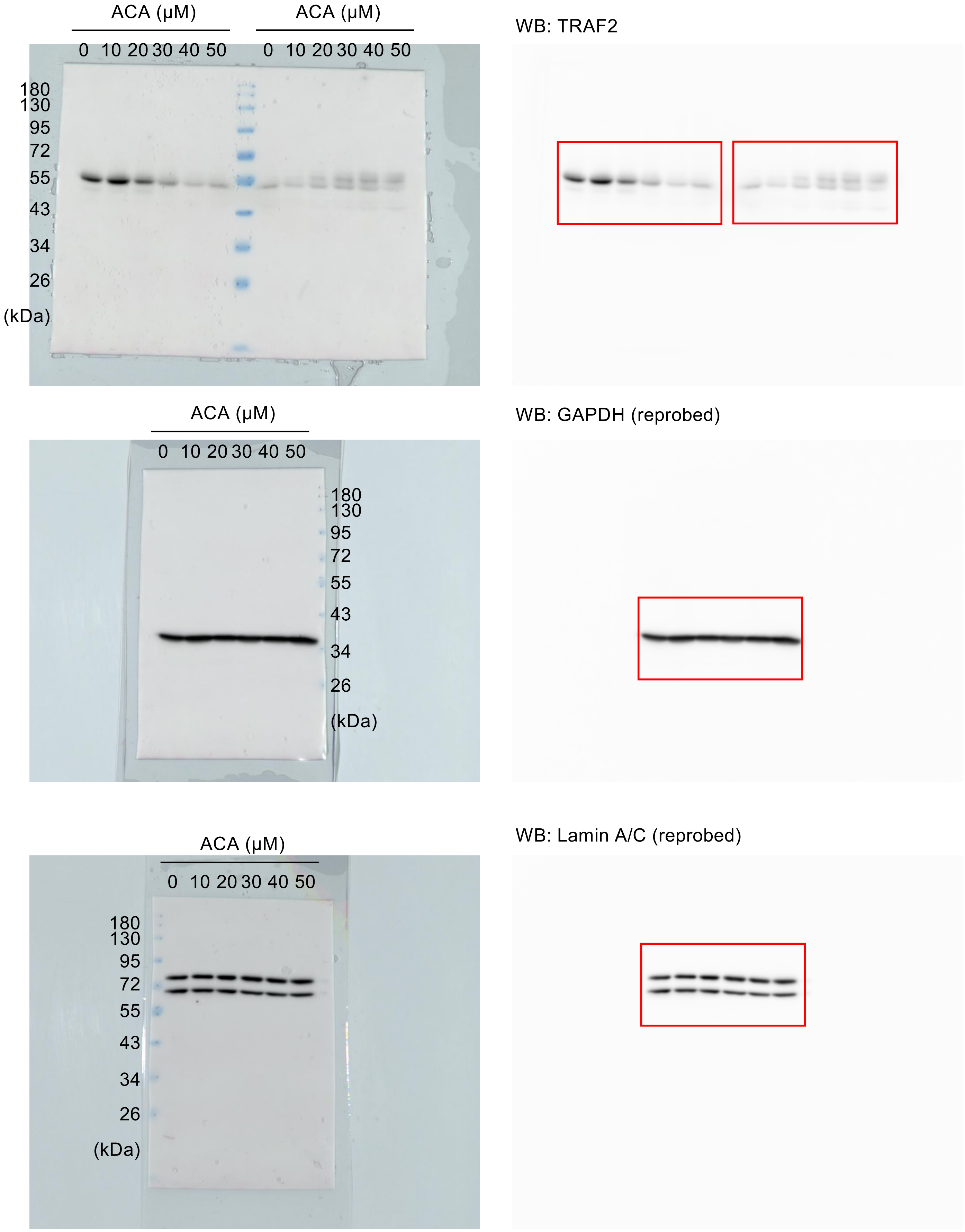

Figure S27: Original blots (1) in Figure 7D

ACA (μM)

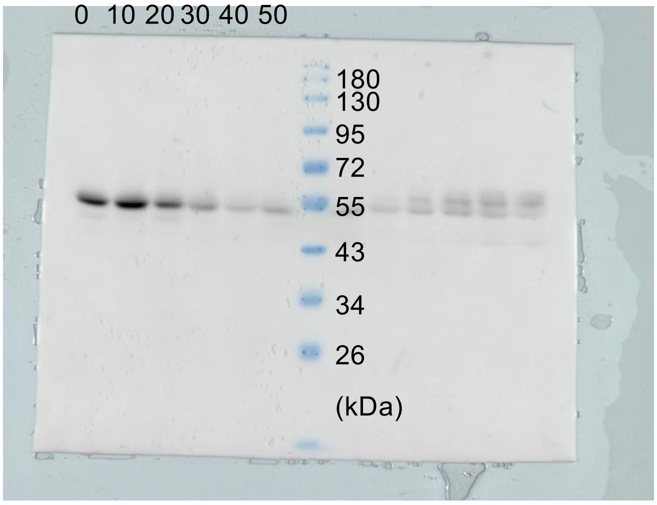

WB: TRAF2

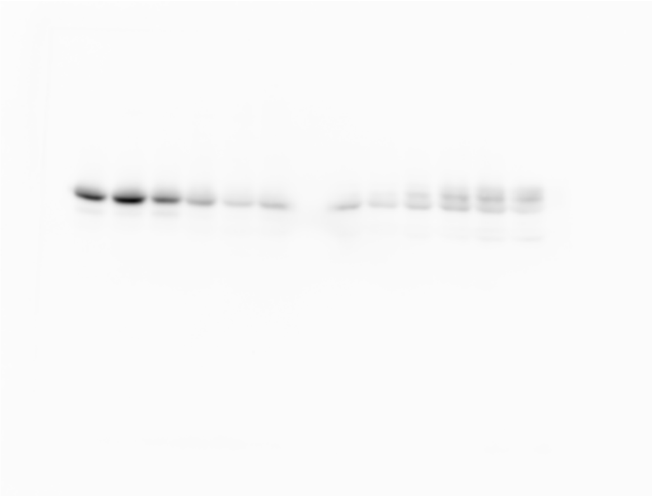

ACA (μM)

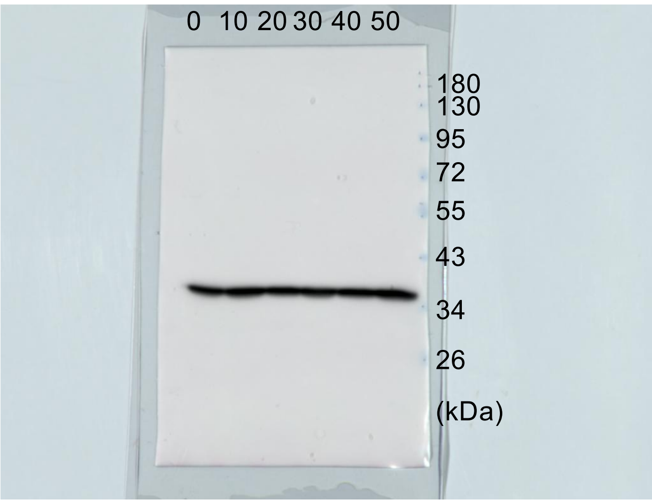

WB: GAPDH (reprobed)

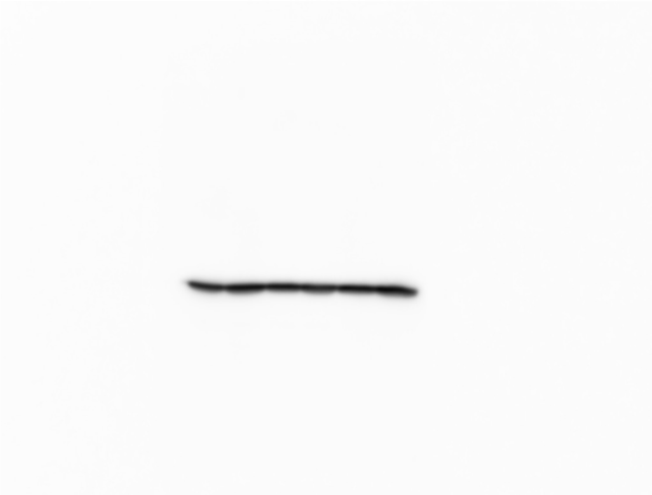

Figure S28: Original blots (2) in Figure 7D

ACA (µM)

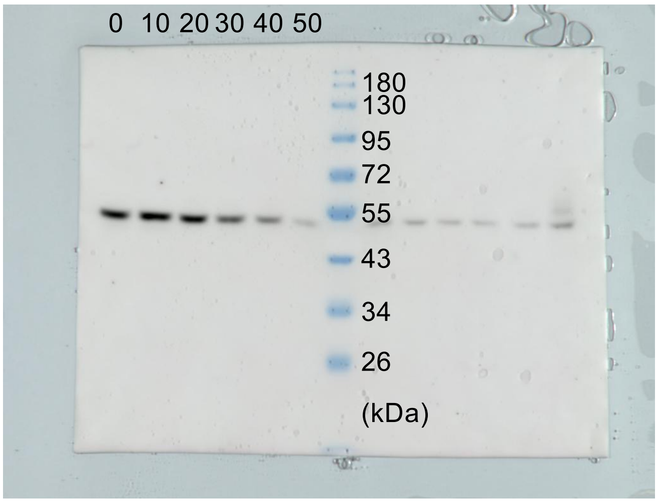

WB: TRAF2

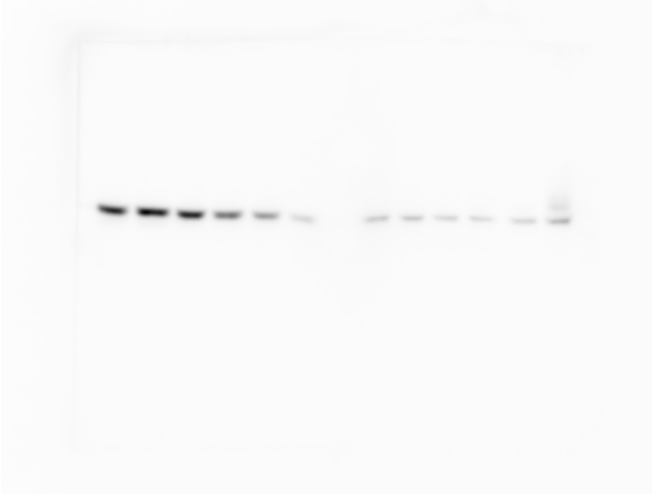

ACA (µM)

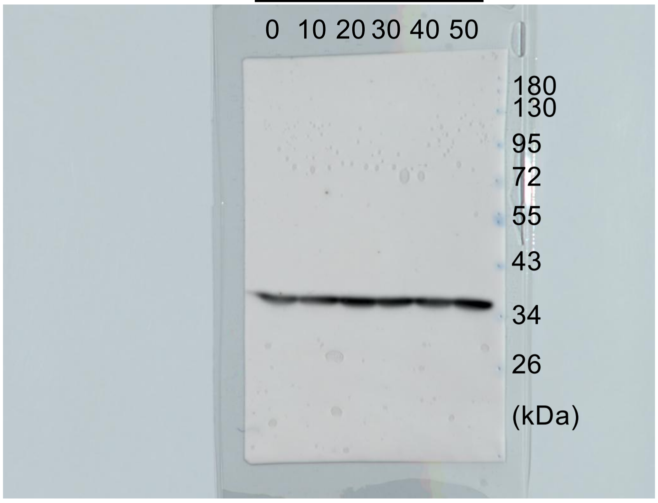

WB: GAPDH (reprobed)

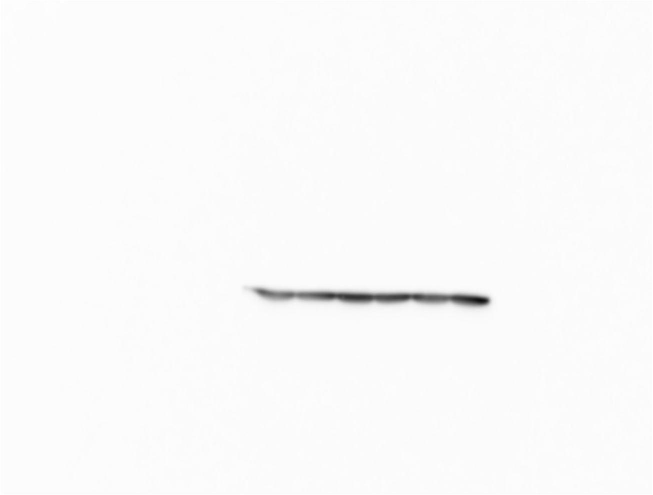

Figure S29: Original blots (3) in Figure 7D

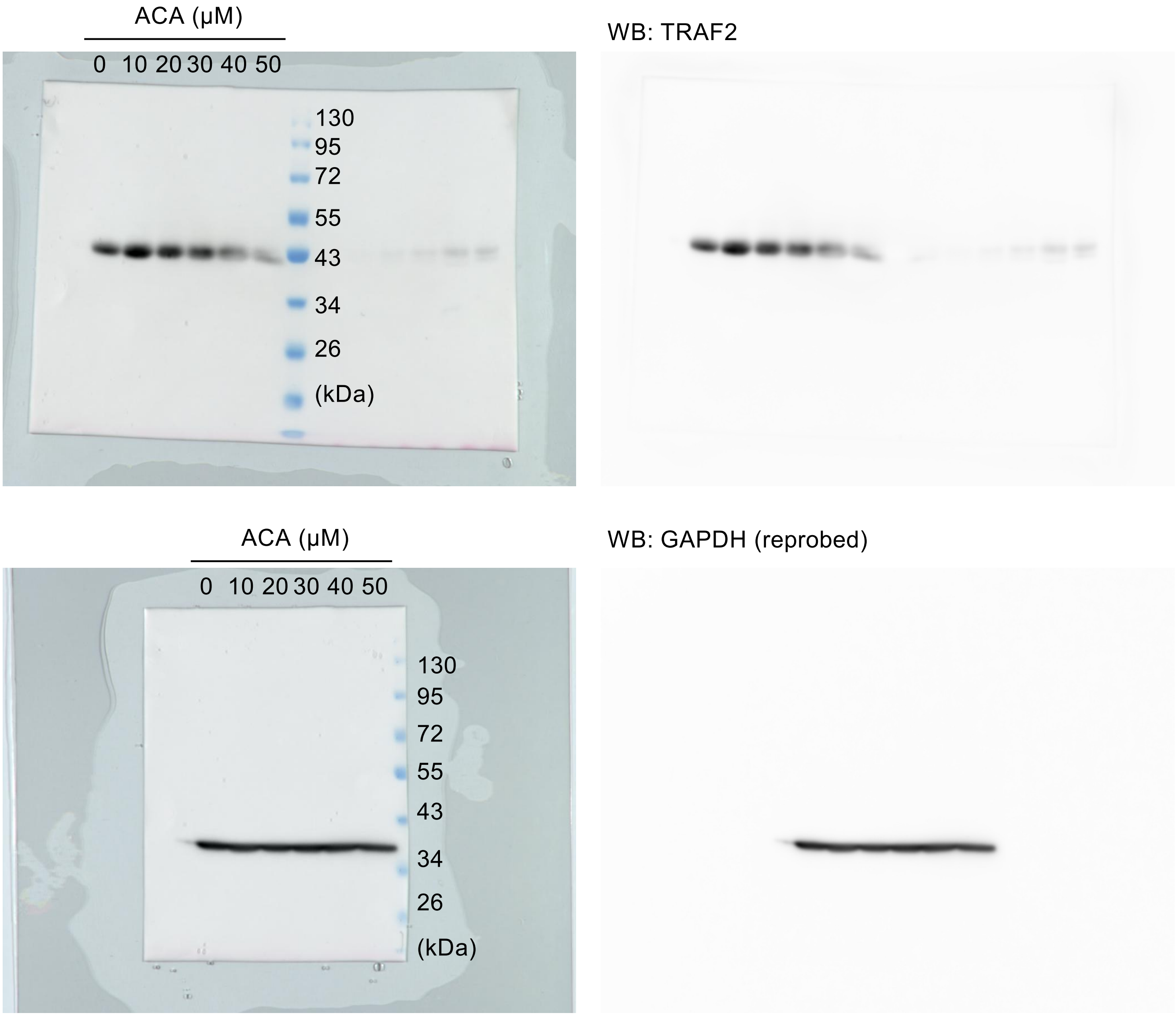

Figure S30: Original blots (1) in Figure 7E

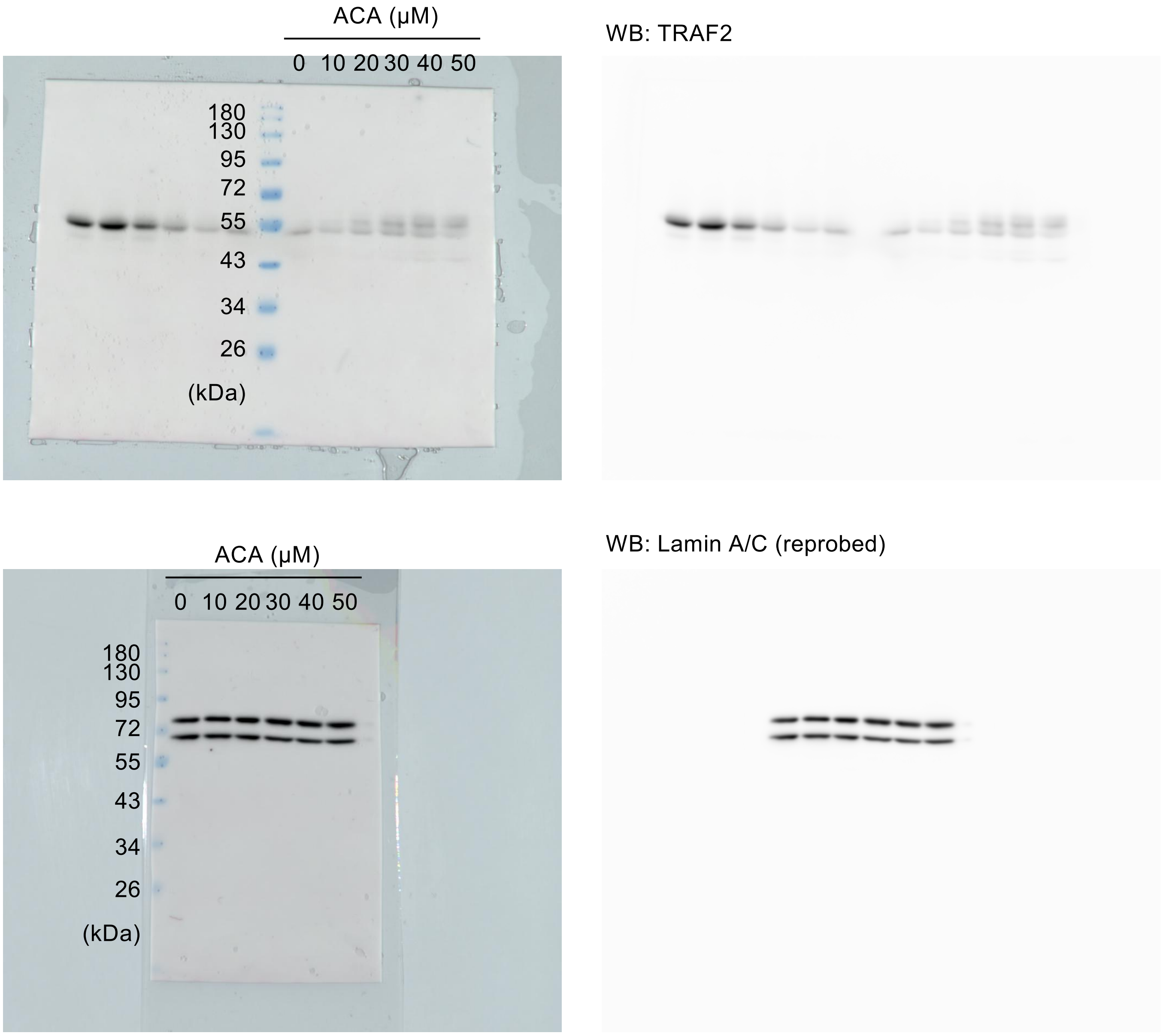

Figure S31: Original blots (2) in Figure 7E

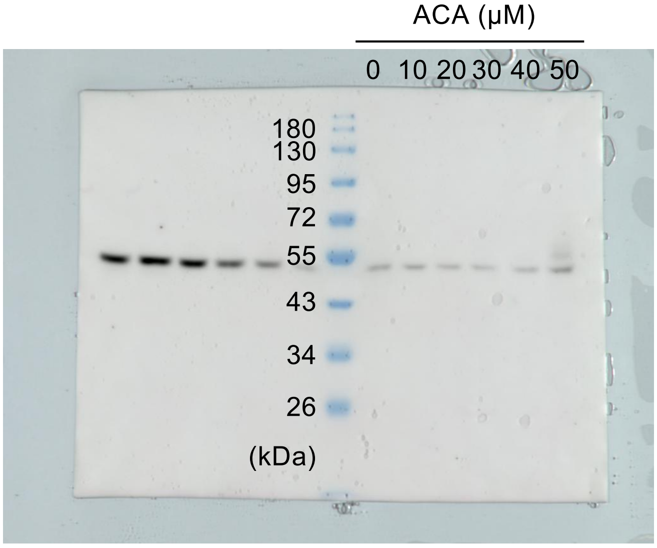

WB: TRAF2

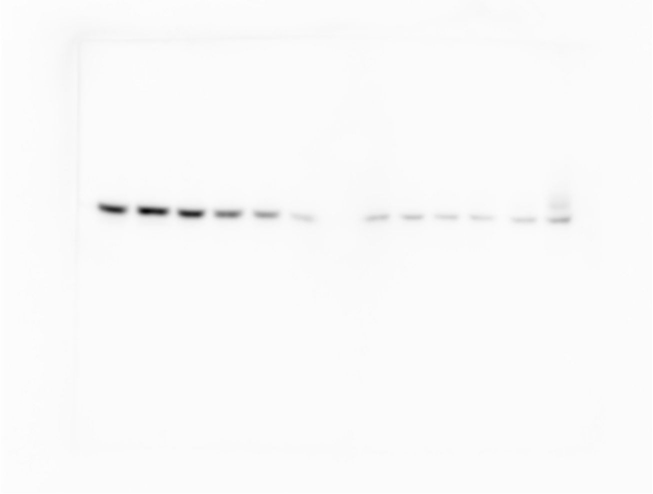

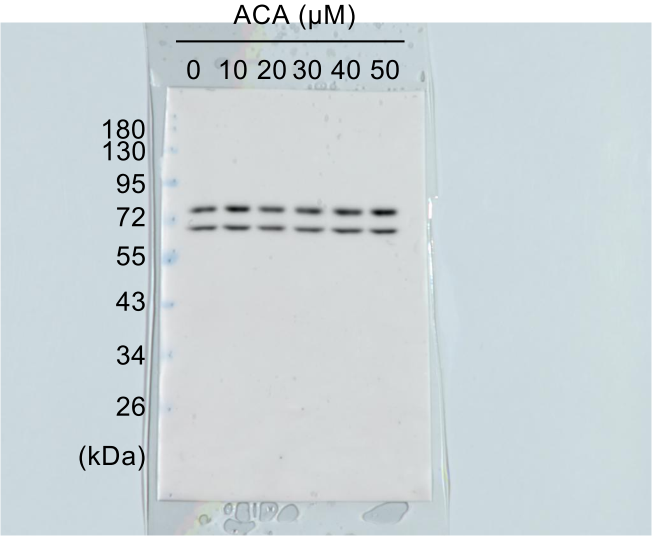

WB: Lamin A/C (reprobed)

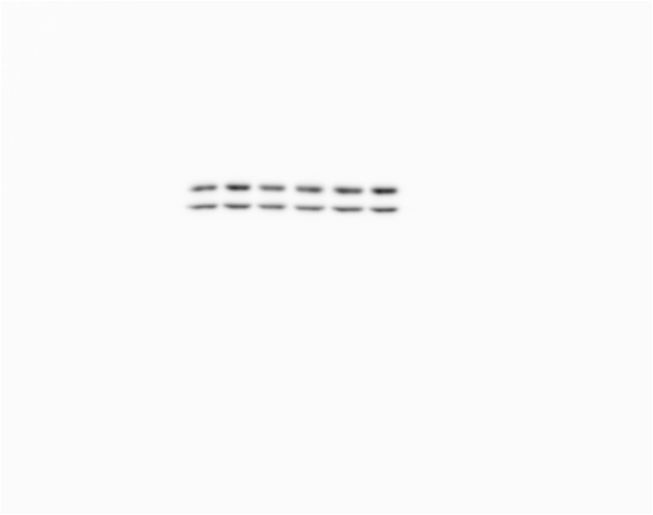

Figure S32: Original blots (3) in Figure 7E

ACA (μM)

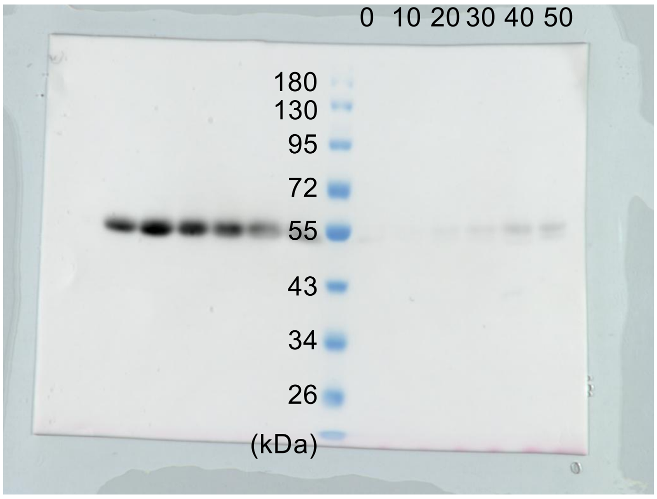

WB: TRAF2

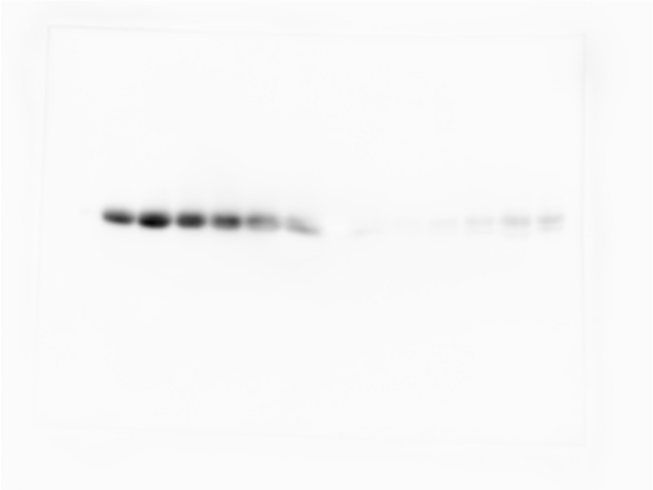

ACA (μM)

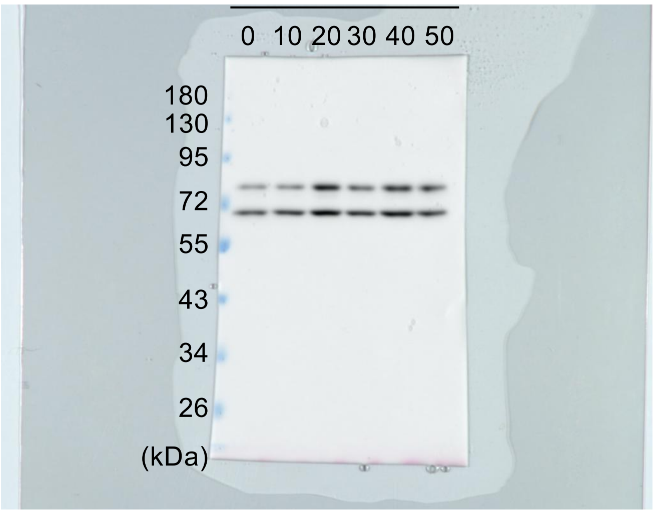

WB: Lamin A/C (reprobed)

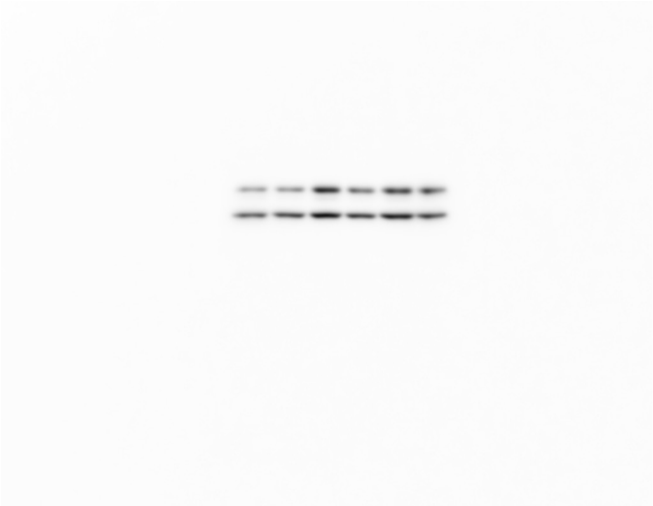

Figure S33: Original blots in Figure 8A

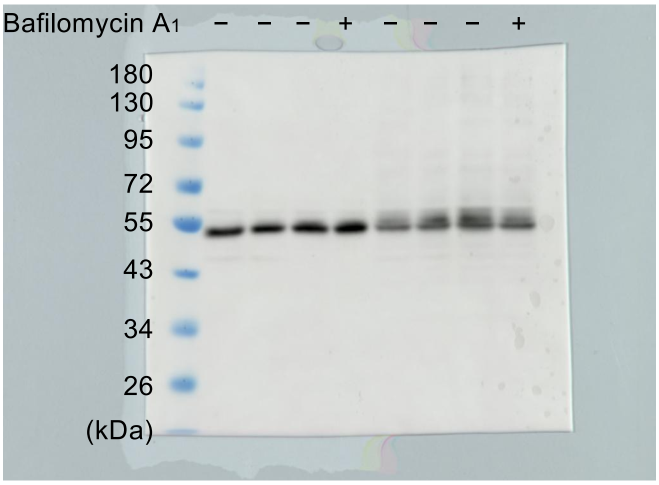

WB: TRAF2

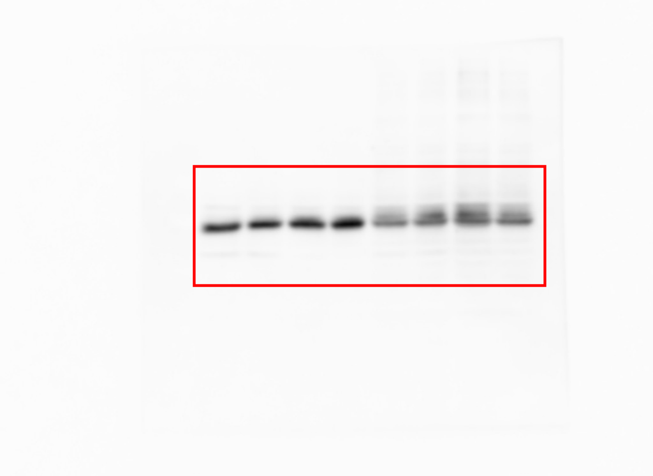

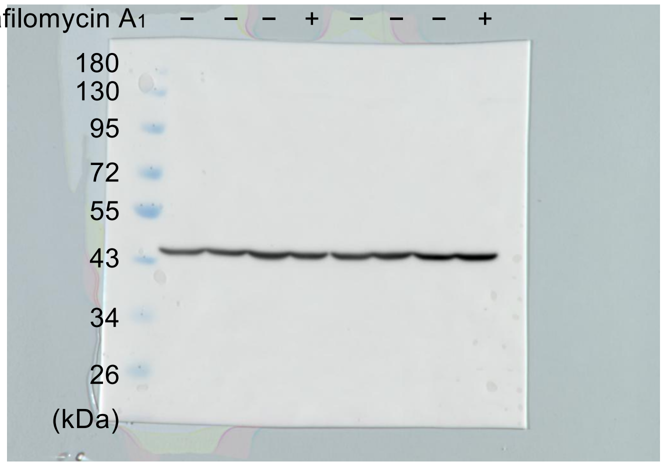

WB: β-Actin (reprobed)

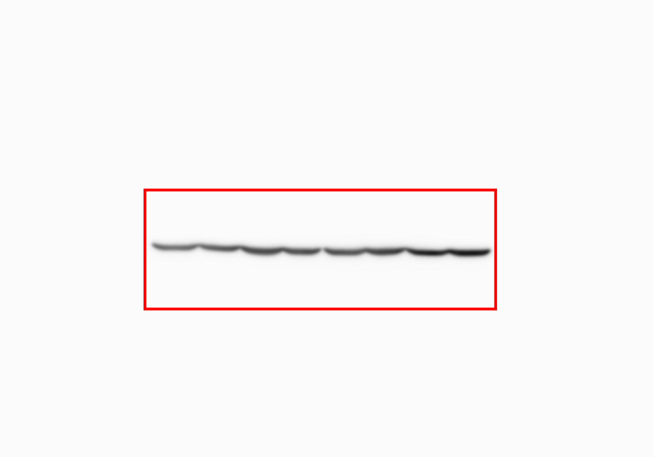

Figure S34: Original blots (1) in Figure 8B

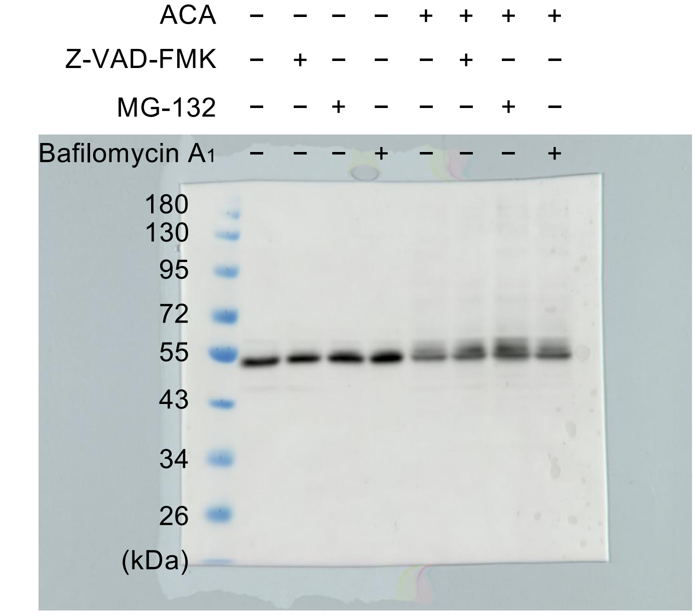

WB: TRAF2

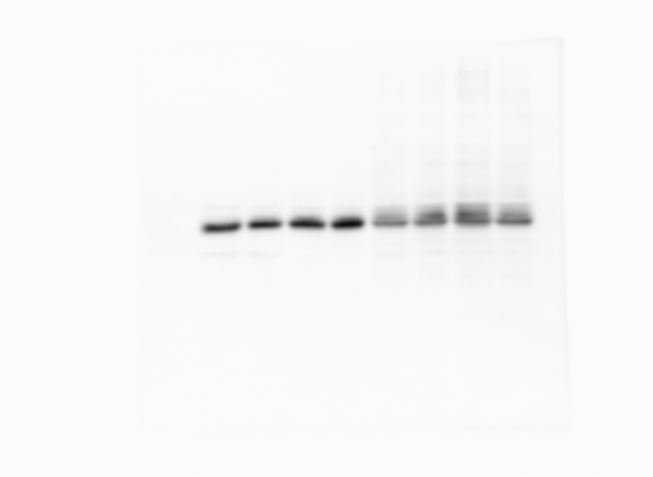

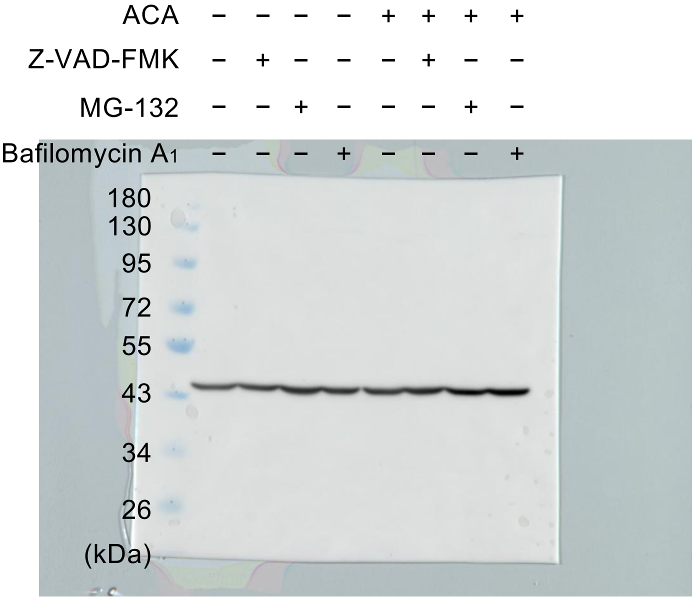

WB: β-Actin (reprobed)

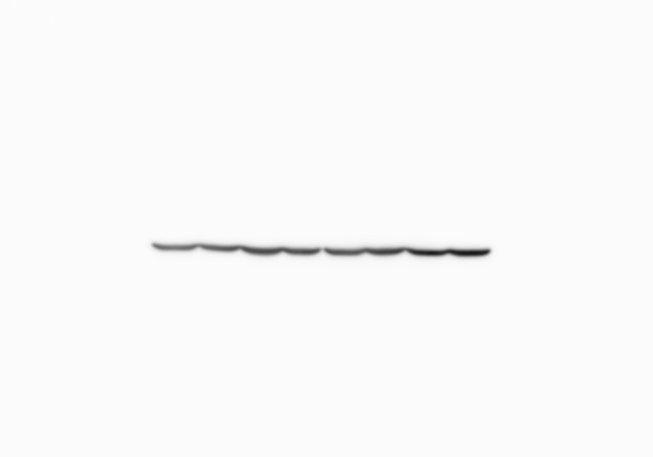

Figure S35: Original blots (2) in Figure 8B

ACA	–	–	–	–	+	+	+	+
Z-VAD-FMK	–	+	–	–	–	+	–	–
MG-132	–	–	+	–	–	–	+	–
Bafilomycin A1	–	–	–	+	–	–	–	+

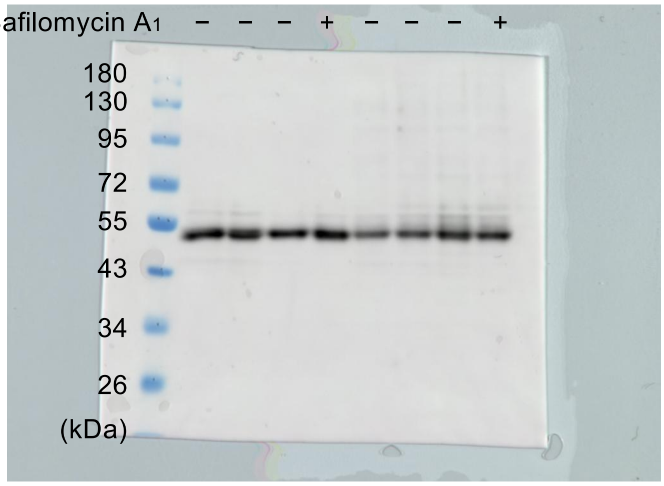

WB: TRAF2

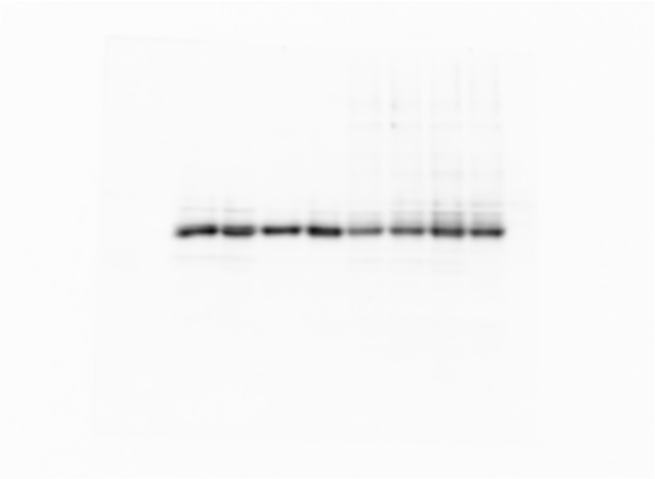

ACA	–	–	–	–	+	+	+	+
Z-VAD-FMK	–	+	–	–	–	+	–	–
MG-132	–	–	+	–	–	–	+	–
Bafilomycin A1	–	–	–	+	–	–	–	+

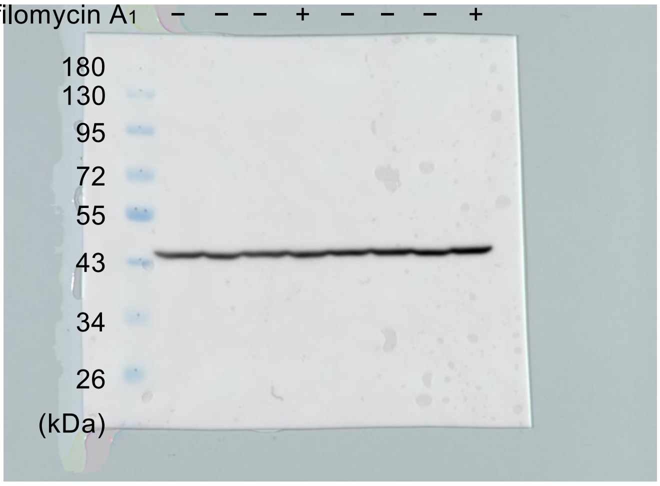

WB: β-Actin (reprobed)

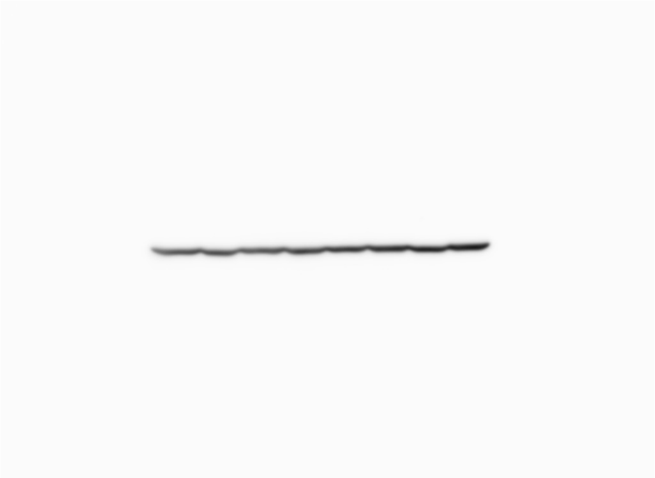

Figure S36: Original blots (3) in Figure 8B

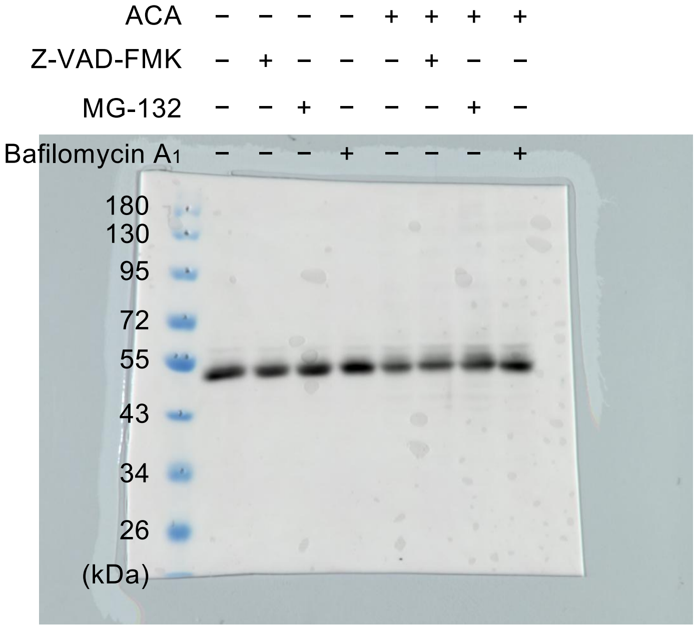

WB: TRAF2

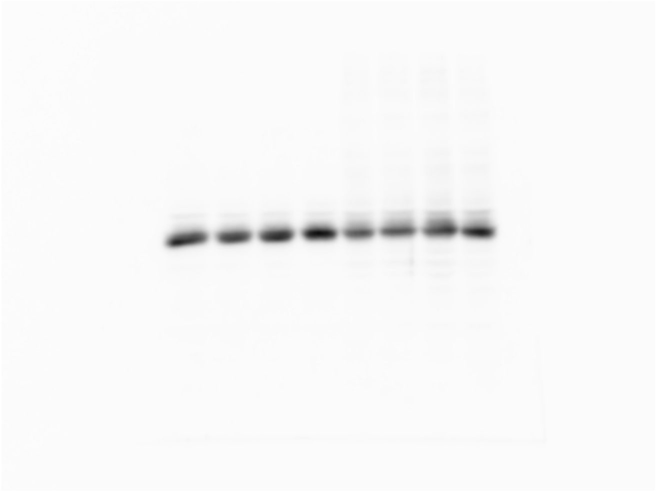

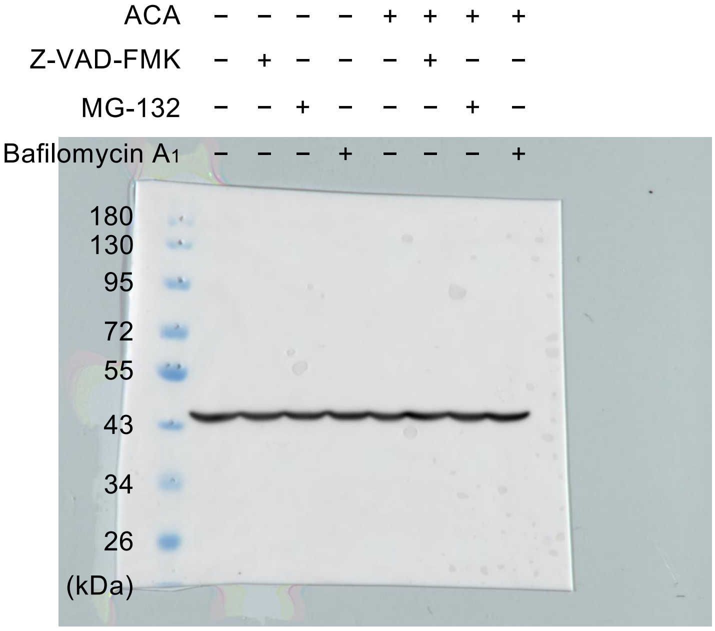

WB: β-Actin (reprobed)

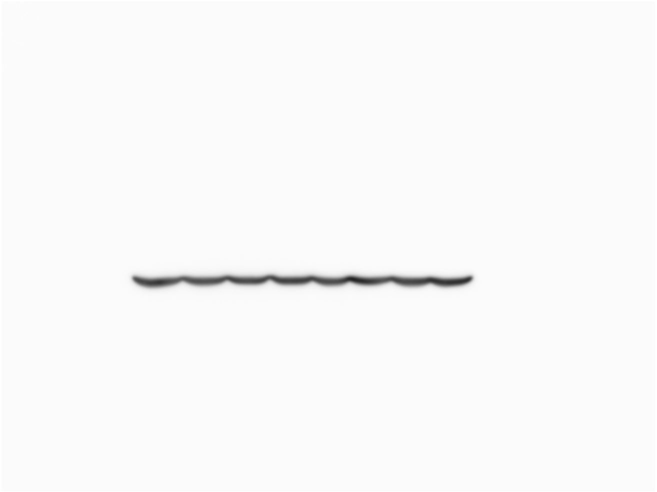

Figure S37: Original blots in Figure 9B

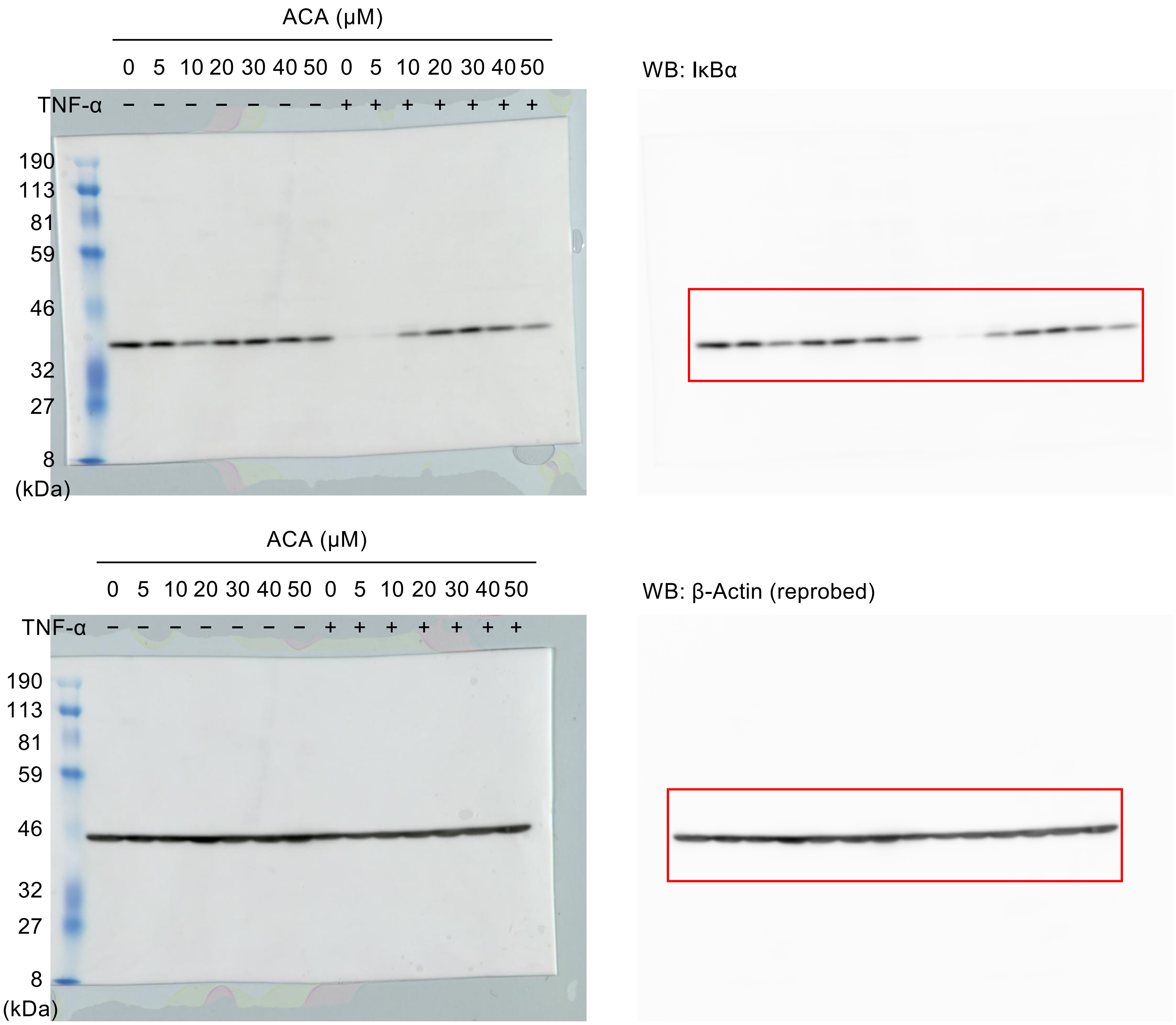

Figure S38: Original blots (1) in Figure 9C

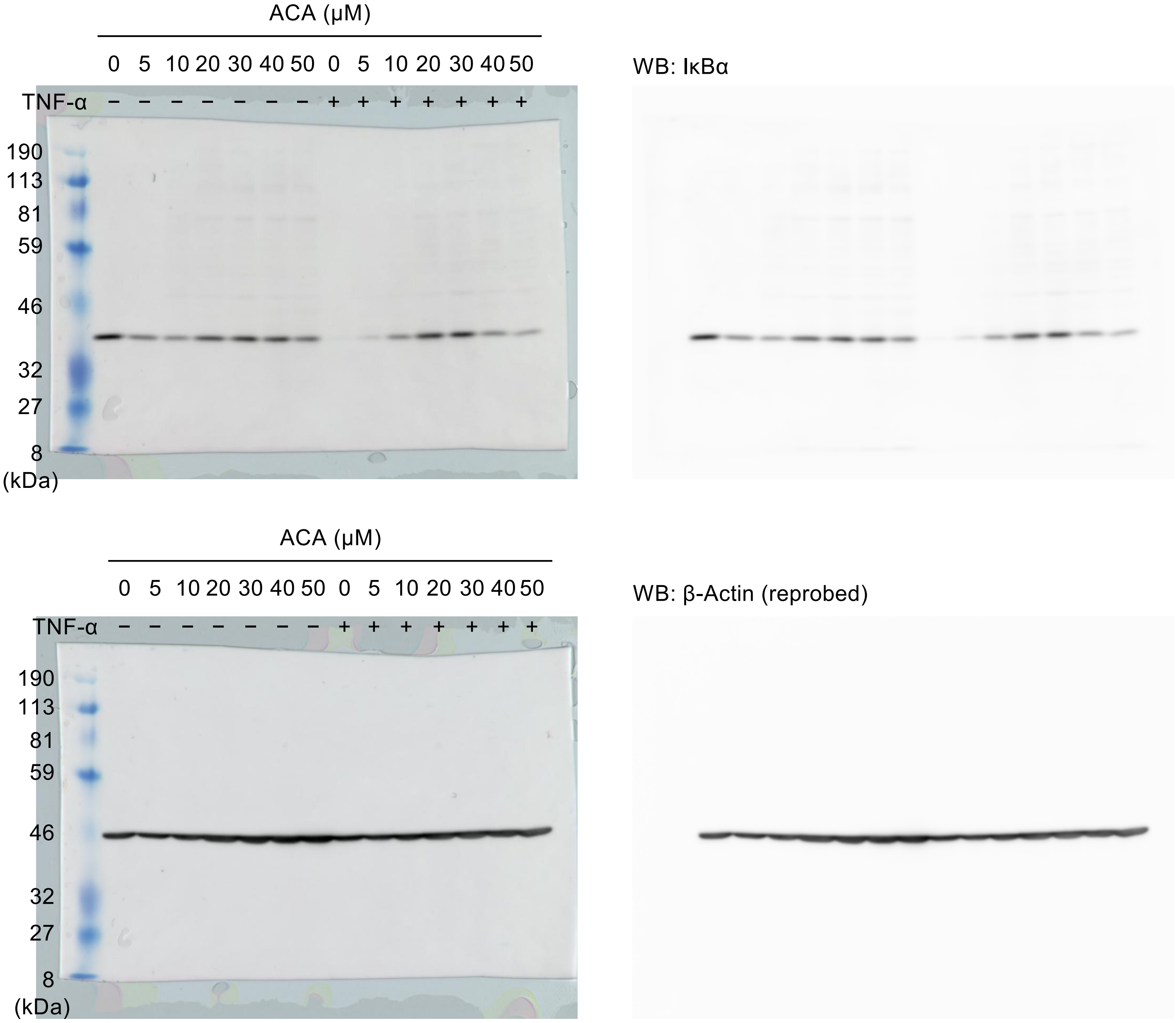

Figure S39: Original blots (2) in Figure 9C

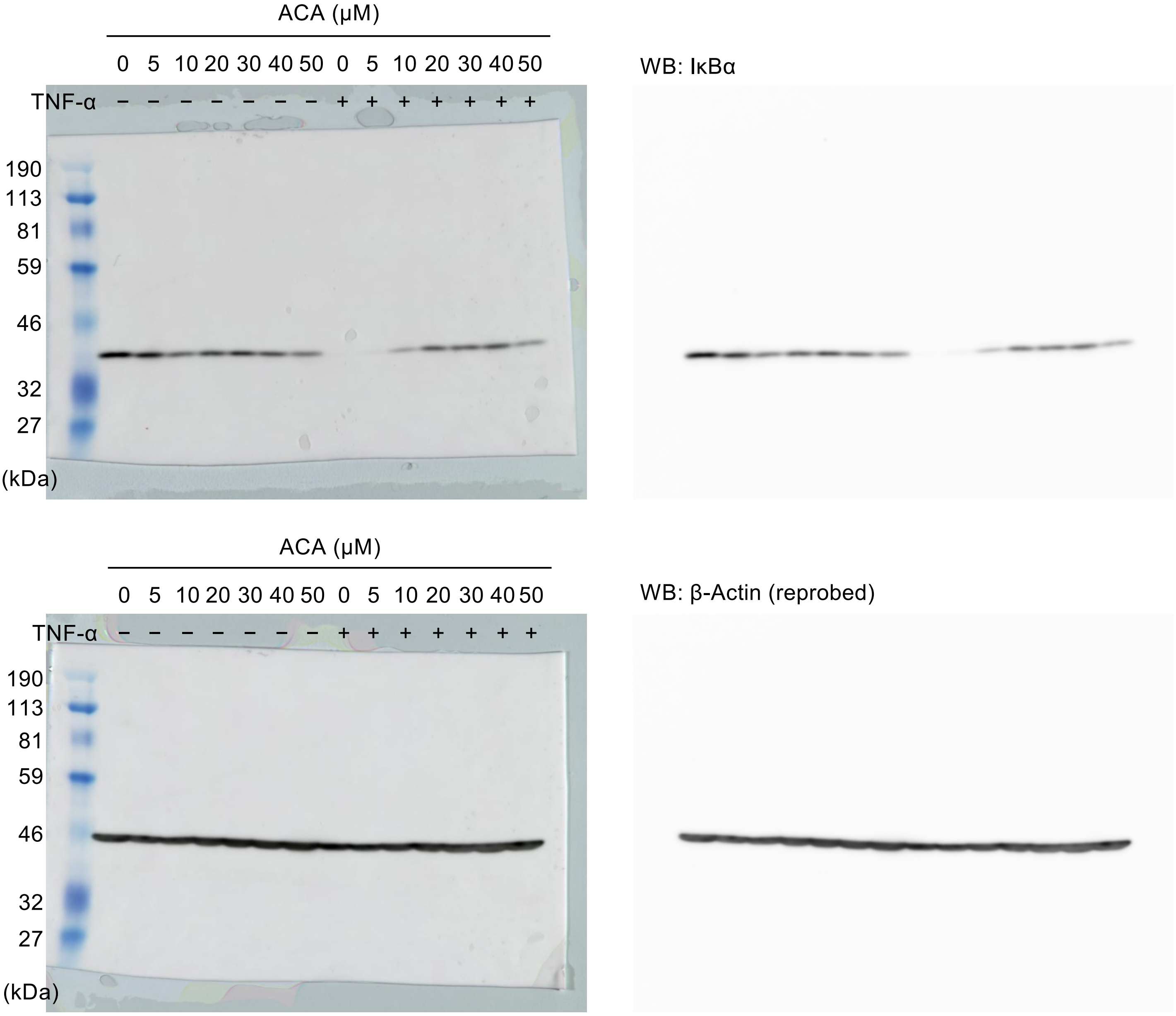

Figure S40: Original blots (3) in Figure 9C

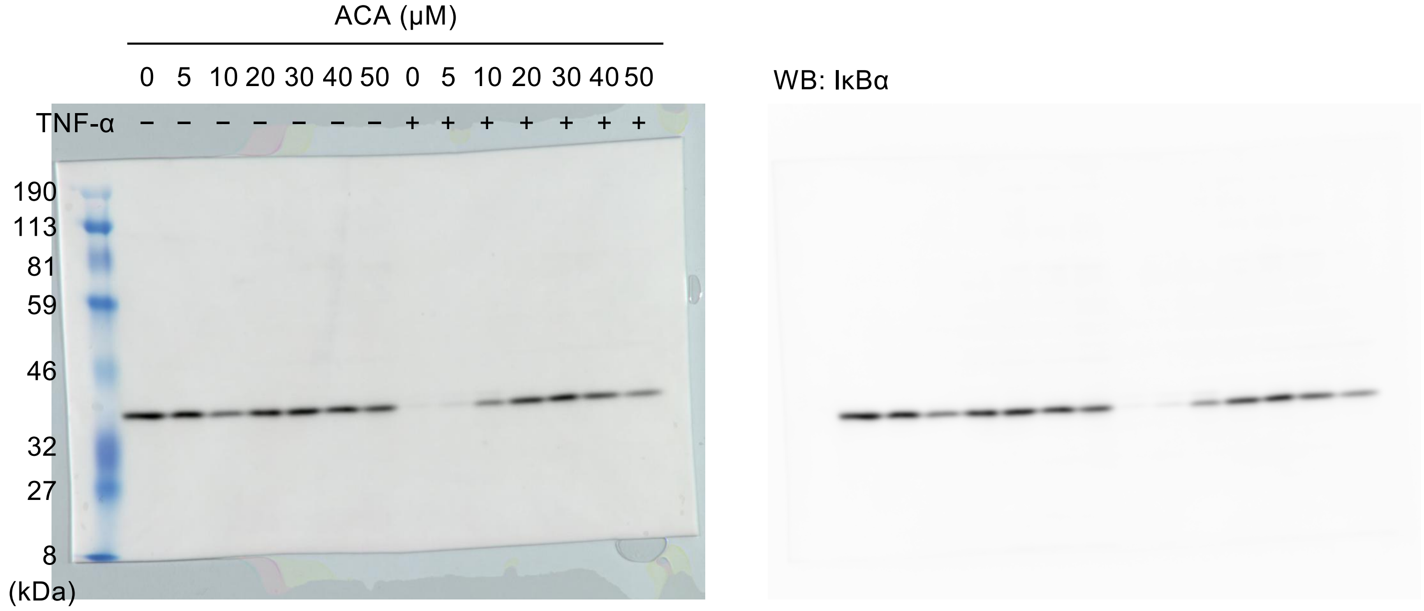

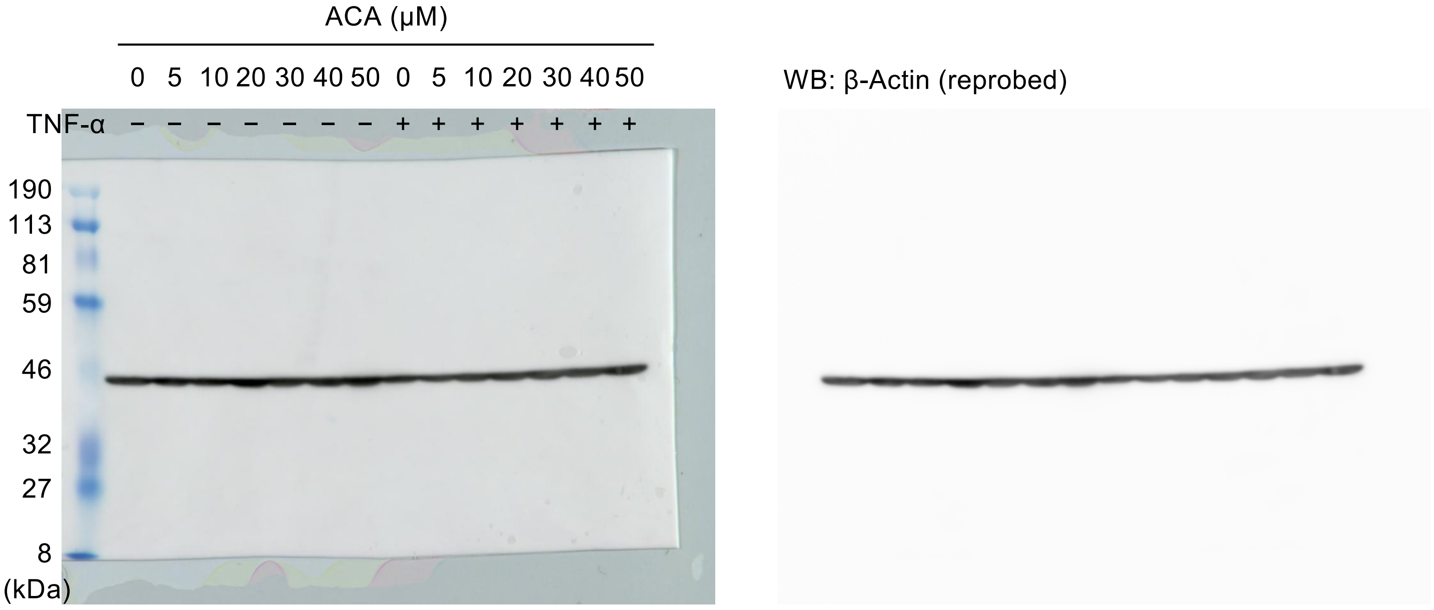

Figure S41: Original blots in Figure 10A

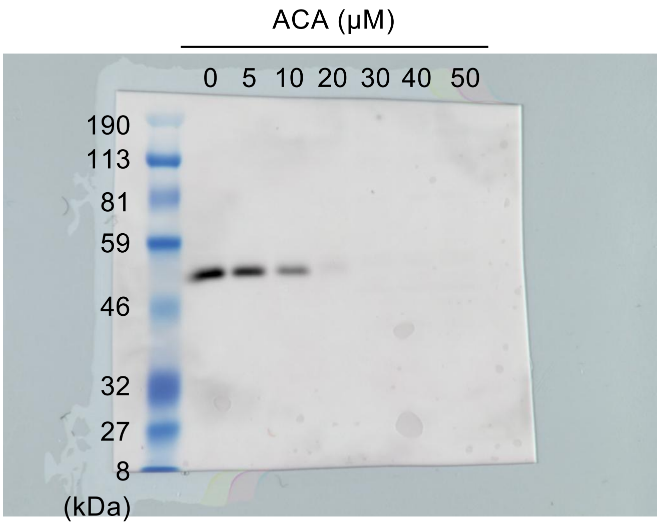

WB: TRAF2

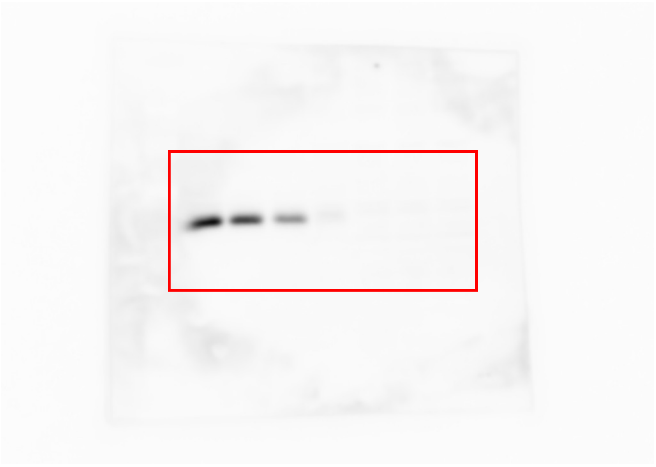

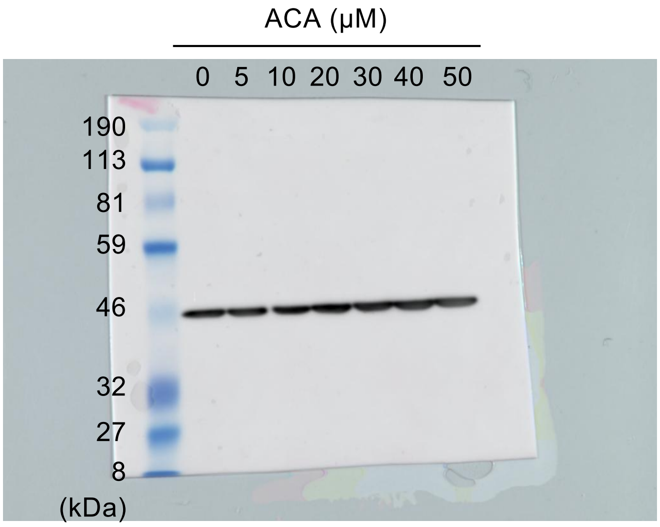

WB: β-Actin (reprobed)

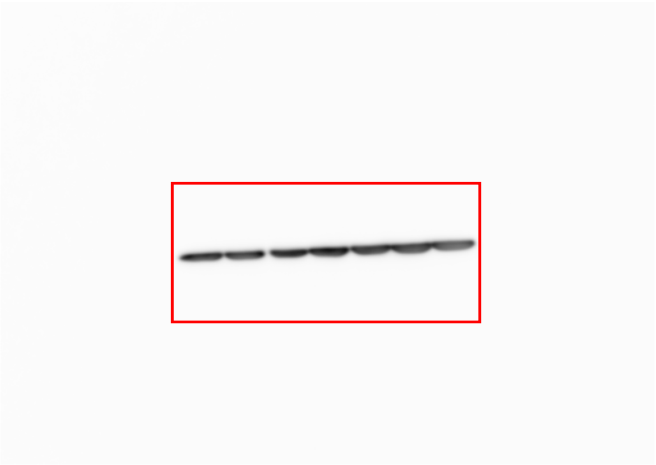

Figure S42: Original blots (1) in Figure 10B

ACA (μM)

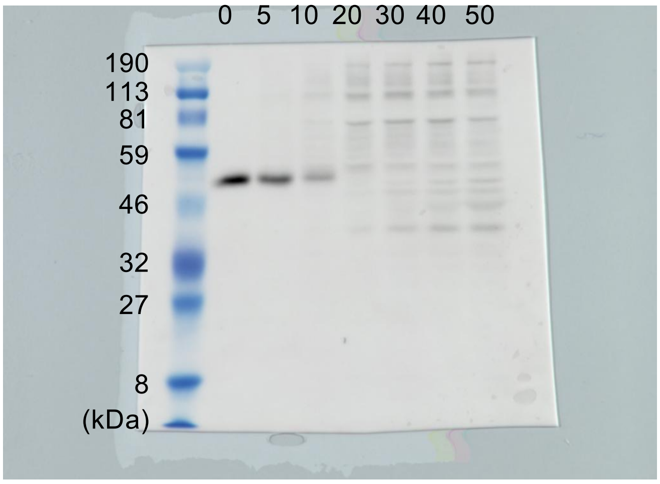

WB: TRAF2

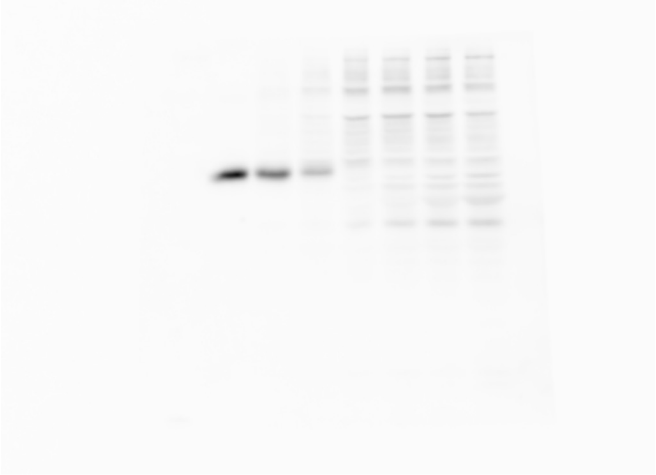

ACA (μM)

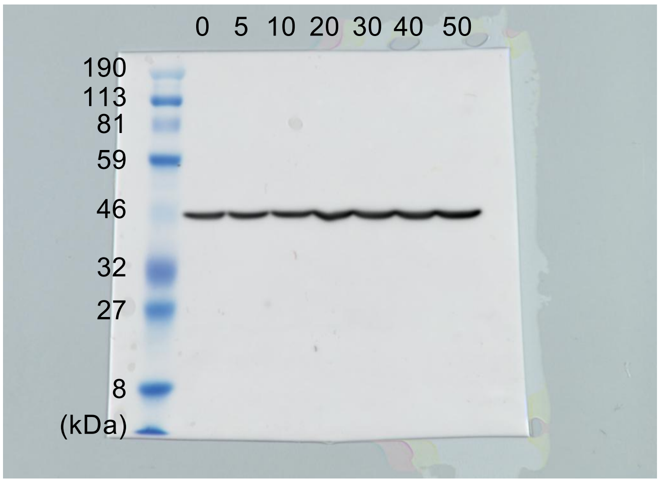

WB: β-Actin (reprobed)

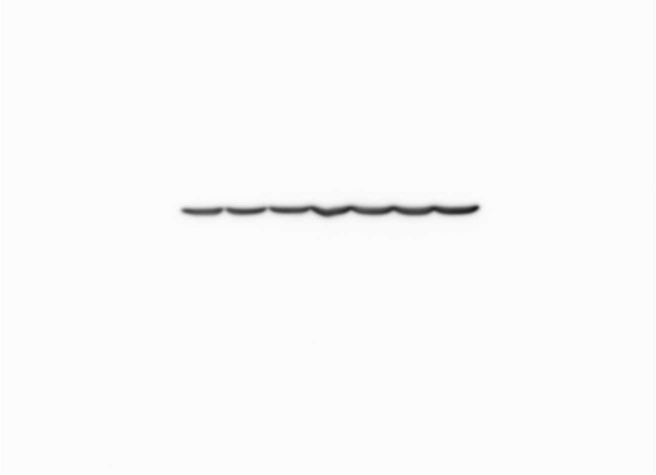

Figure S43: Original blots (2) in Figure 10B

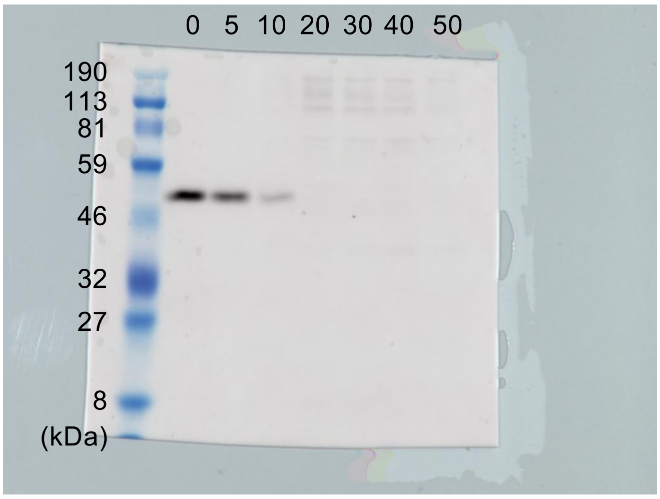

WB: TRAF2

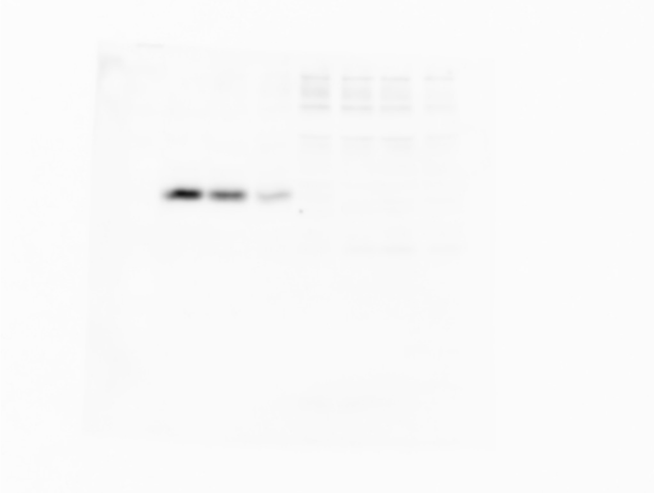

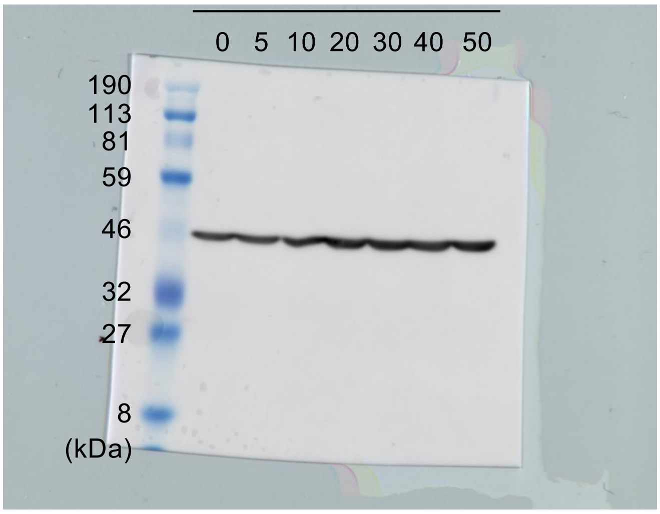

WB: β-Actin (reprobed)

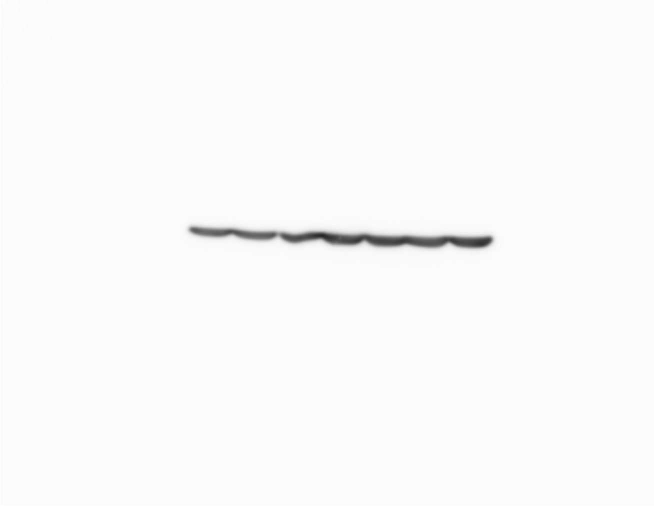

Figure S44: Original blots (3) in Figure 10B

ACA (μM)

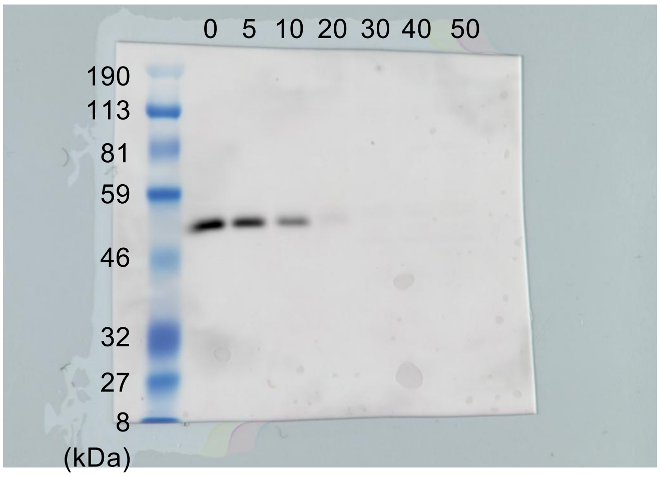

WB: TRAF2

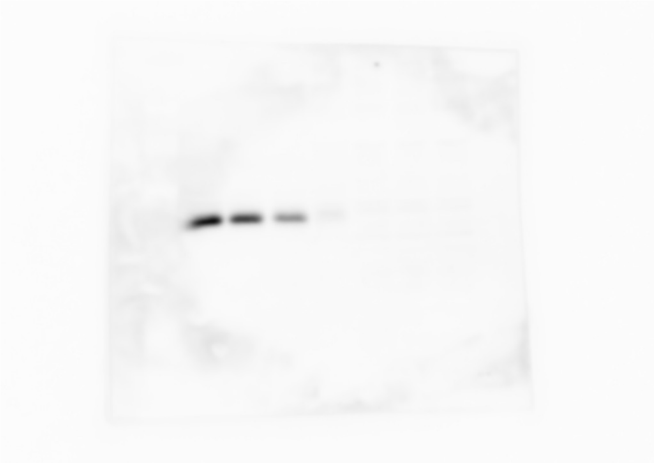

ACA (μM)

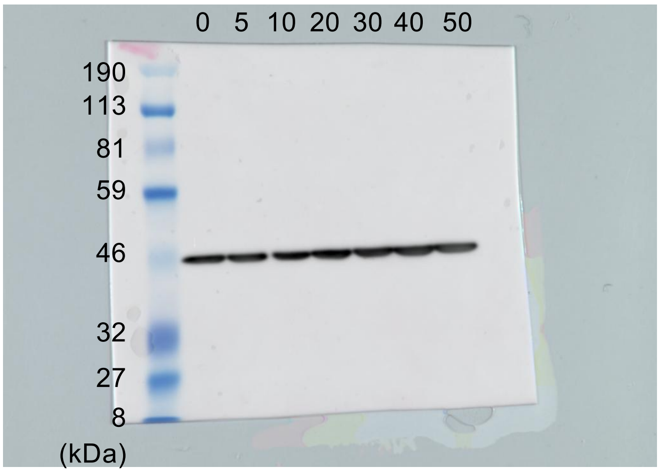

WB: β-Actin (reprobed)

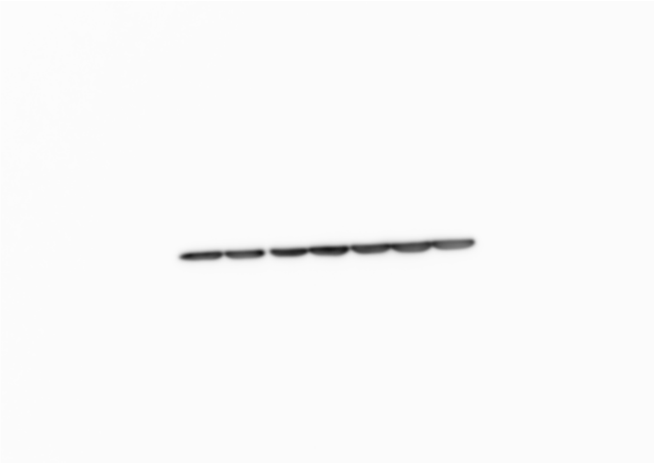

Supplement: Supplementary file 1 [file molecules-30-01243-s001.zip › molecules-3386497-supplementary.pdf]
